# First record of a basal mammaliamorph from the early Late Triassic Ischigualasto Formation of Argentina

**DOI:** 10.1371/journal.pone.0218791

**Published:** 2019-08-07

**Authors:** Rachel V. S. Wallace, Ricardo Martínez, Timothy Rowe

**Affiliations:** 1 Jackson School of Geosciences, The University of Texas at Austin, Austin, Texas, United States of America; 2 División Paleontologia de Vertebrados, Instituto y Museo de Ciencias Naturales, Universidad Nacional de San Juan, San Juan, Argentina; Royal Belgian Institute of Natural Sciences, BELGIUM

## Abstract

We describe a new probainognathian cynodont, *Pseudotherium argentinus*, from the early Late Triassic Ischigualasto Formation of Argentina. *Pseudotherium* adds to a growing assemblage of small Triassic cynodonts that offers new insight into events leading up to the origin of crown Mammalia and the successively more inclusive Mammaliaformes and Mammaliamorpha. Using high-resolution X-ray computed tomography, we illustrate and describe the holotype and only known specimen, which consists of a well-preserved isolated skull. It preserves apomorphic features of the orbit and braincase. Prefrontal and vestigial postorbital bones are present, despite the absence of an ossified postorbital bar. As in *Brasilitherium riograndensis*, thin turbinal-like bones are present in the nasopharyngeal passage, and we discuss impediments to establishing their identity and function. Compared to more basal cynodonts, the cochlea is elongated but uncoiled and in this and other features it resembles basal mammaliamorphs. Our analysis found weak support for *Pseudotherium* as the sister taxon of Tritylodontidae. However, a broader assessment of its relationships in light of additional character data from the literature and unpublished computed tomography data suggest that it may be more realistic to view the relationships of *Pseudotherium* as an unresolved polytomy with tritylodontids, and the taxa referred to as tritheledontids and brasilodontids (groups of variable membership and questionable monophyly). Thus, *Pseudotherium* may lie just inside or just outside of Mammaliamorpha, and there is also weak character support for its sister taxon relationship with *Brasilitherium*. Our results amplify previous conclusions that phylogenetic relationships in this adaptive radiation of small cynodonts will remain somewhat uncertain until more complete specimens are recovered, and until high-resolution CT scans of existing specimens become available to the larger community. Toward that goal, we make the CT dataset for the holotype of *Pseudotherium argentinus* publically available under a Creative Commons license at www.DigiMorph.org.

## Introduction

One of the major transformations in vertebrate evolution occurred in a series of events leading up to the origin of crown Mammalia [1]. The transformation, which occurred by or before the Middle Jurassic, took place as several pulses of expansion of the relative size of the brain (encephalization) and the emergence of the uniquely mammalian neocortex [2]. This was probably driven in part by a ten-fold duplication in olfactory receptor genes that induced hypertrophy of the olfactory bulbs and olfactory (pyriform) cortex, as well as the cerebellum and brainstem. In their epigenetic responsiveness to the neurosensory system, the skull, craniovertebral joint and neck were profoundly modified [3–7]. The shift in position and function of mammalian auditory ossicles were also part this transition. From their plesiomorphic position at the jaw joint and dual function in feeding and audition, the auditory ossicles became detached from the jaw and decoupled from feeding to take their characteristic mammalian position suspended beneath the otic capsule and functioned solely in hearing. Controversy surrounds whether the incorporation of these jaw elements into the otic capsule occurred once [3,4,7], or multiple times via different mechanisms (e.g., [8,9]). Also involved in the mammalian transition was the evolution of endothermy, lactation, parental care, and prolonged activity [10,11]. A suite of other skeletal modifications include: formation and lengthening of the secondary palate, an occlusal dentition with roots deeply implanted by a periodontal ligament, ossification of the alisphenoid, and the double occipital condyle. These and other features may be related to the integration of orthonasal olfaction, retronasal olfaction, taste, and somatosensation from the teeth and tongue, into a larger sensory system that has been termed ‘ortho-retronasal olfaction,’ which may have played a role in mammalian cortical evolution [5,7].

While the fossil record provides a fairly dense taxonomic sample of stem-mammals, another well-known feature of this record is that the closest extinct relatives of Mammalia among Cynodontia were small, and many taxa are known only from isolated teeth [11,12]. Most of the Late Triassic and Jurassic non-mammalian cynodonts occupy the two smallest orders of vertebrate size magnitude, and it was not until the Cenozoic that the independent evolution of large body size began to characterize various mammalian clades [10,13].

One of the key nodes on the mammalian stem is Mammaliaformes, the clade originating in the last common ancestor shared by (crown) Mammalia and the Late Triassic *Morganucodon oehleri* [1,14,15]. Most of the osteological features that currently diagnose Mammaliaformes are correlated with increasing brain size, and the evidence now available suggests that at least a small neocortex had differentiated in the dorsal cortex of the telencephalon [2,5]. Integumentary evidence from the remarkably preserved Early Jurassic Chinese fossil *Castorocauda* [16] suggests that a pelt of modern aspect, with guard hairs and velus underfur, was present at or very shortly after the origin of Mammaliaformes, but prior to the origin of Mammalia itself. Interdependencies discovered in the ontogeny of living mammals demonstrate that the development of innervated hair follicles induces somatosensory maps on the neocortex. The presence of guard hairs in an early mammaliaforms suggests that at least a small neocortex had differentiated, and within it a primary somatosensory field [2,5]. The origin of Mammaliaformes thus involved a marked pulse in encephalization in which the brain and skull began to look and function more like those of living mammals than to more primitive cynodonts of the Early and Middle Triassic, most of which retained a narrow, tubular forbrain and relatively small olfactory bulbs.

Mammaliamorpha [1] is a more inclusive clade which stems from the last common ancestor shared by Mammalia and the extinct clade Tritylodontidae. Mammaliamorpha is diagnosed by many features of the skull and postcranial skeleton [1,7,14,15]. However, the many characters which offer strength to the diagnosis may simply mark the long expanse of missing fossil record that pre-dates the origin of Mammaliamorpha. In other words, with new discoveries of more-basal Triassic fossils we should expect that at least some of the diagnostic features of mammaliamorphs will prove to have wider, more inclusive distributions along the mammaliamorph stem. A number of features of the new taxon described here demonstrate this point.

The origin of Mammaliamorpha is coming into sharper focus with recent discoveries of new Late Triassic and Early Jurassic cynodont fossils that clearly possess complex assemblages of derived features that were passed on to crown mammals. These fossils suggest a global diversification of small cynodonts whose members lie just within Mammaliamorpha, or just outside on its stem. Untangling these relationships is critical to fully understanding the evolutionary sequence of transformations involved in mammalian origins. As described below, subtle skeletal features suggest that the origin of Mammaliamorpha involved neurosensory modifications that occurred earlier than previously known, setting a stage for the more profound neurosensory transformations seen in early members of Mammaliaformes, and later in the origin of Mammalia [2].

The comparative framework for evaluating the new taxon involves a number of Triassic and Jurassic fossils that, according to current hypotheses, are within or just ancestral to the clade Mammaliamorpha. We informally refer to them as the ‘taxa of interest’ because they have the most direct bearing on understanding the phylogenetic position of *Pseudotherium*. These cynodonts occupied the two smallest orders of vertebrate size magnitudes, and they are known mostly from partial skulls and partial post-cranial skeletons. Owing to their small size, however, these fossils have proven difficult to prepare and to study in detail using conventional methods. This material is also scattered across widely separated museum collections and few researchers have had the opportunity to study all the relevant material first-hand. The literature is variable in its depth of description and in its quality of illustration. As a result, a measure of uncertainty surrounds anatomical interpretations of the specimens as well as their phylogenetic relationships. Uncertainty also attends the precise sequence of events that culminated in the origin of Mammalia, and this in turn has fueled controversy over which character transformations over pan-mammalian history were affected by homoplasy.

The ‘taxa of interest’ include the well-known Late Triassic to Middle Jurassic Tritylodontidae, which is the sister taxon to all other mammaliamorphs [1,15]. Also of interest are the taxa referred to Brasilodontidae. This is a problematic taxon of questionable monophyly, based on several small, incomplete specimens. They include *Brasilodon quadrangularis* [17,18], *Brasilitherium riograndensis* [17,18], *Minicynodon maieri* [19], and *Protheriodon estudianti* [20], from the Middle and Late Triassic of Brazil and Argentina; and *Panchetocynodon damodarensi* from the Early Triassic of India [21,22]. Bonaparte [21] considered Brasilodontidae to be monophyletic and to be the sister taxon to Mammaliaformes (his Mammalia), but his taxon sampling omitted Tritylodontidae, leaving his results equivocal with respect to membership in Mammaliamorpha. Analyses by Luo [8], scoring only *Brasilitherium*, and Liu and Olson [23], scoring *Brasilodon*, and considering *Brasilitherium* as its junior synonym (see [24–27] for discussion of brasilodontid taxonomy), found them to lie within Mammaliamorpha, but outside of Mammaliaformes. Abdala [28] found the group to be paraphyletic, with *Brasilitherium* as a sister taxon to Mammaliaformes, and *Brasilodon* as the sister taxon to Mammaliamorpha + *Pachygenelus* (representing Tritheledontidae, below).

Additional taxa of interest are the Late Triassic to Early Jurassic species referred to Tritheledontidae (*=* Ictidosauria [29,30]; *=* Diarthrognathidae, *sensu* [31]), another group of uncertain monophyly. From the Late Triassic of Argentina and Brazil, the best known include *Riograndia guaibensis* [32,33] and *Chaliminia musteloides* [34,35], and possibly also *Irajatherium hernandezi* [36,37]. Also included are *Elliotherium kersteni* [38], from the Late Triassic of South Africa, and *Pachygenelus monus* [39] and *Tritheledon riconoi* [40] from the Early Jurassic of South Africa. Fragmentary specimens from other parts of the world have been referred to Tritheledontidae (see reviews by [35,36,38]).

Recent reviews of Tritheledontidae have all found weak support for its monophyly [24,35,36,38]. All of the known specimens are small and incomplete, and the few known postcranial elements are largely undescribed. The name has been used for different sets of taxa by different authors, and the phylogenetic results of different studies are difficult to compare owing to different taxon sampling. With that caveat, some authors infer Tritheledontidae to lie within Mammaliamorpha (e.g., [8,17,41–44]), while others have placed it just outside (e.g., [15,24,28,45]). Lucas and Luo [46] obtained both results, with Tritheledontidae just inside or just outside of Mammaliamorpha. Sidor and Hancox [38] and Martinelli and Rougier [35] addressed relationships among tritheledontids but neither study included Tritylodontidae, hence their results are uninformative with respect to its inclusion in Mammaliamorpha. Liu and Olsen [23] inferred tritheledontids to be paraphyletic, with *Pachygenelus monus* and *Riograndia guaibensis* as successive outgroups to Mammaliamorpha.

Here, we describe a new fossil cynodont from the early Late Triassic Ischigualasto Formation of Argentina using micro-computed tomography (μCT) of an isolated skull. Computed tomography (CT) and μCT have advanced steadily in versatility and resolution over the last three decades [47–51] and they have now been used to scan a few of the more important fossils from the mammalian stem, as well as many extinct and extant crown mammals (e.g., [2,52–54]). The specimen scanned well, showing marked X-ray contrast between matrix and bone that permitted digital preparation of features that would not have been possible using conventional mechanical preparation. As a result, the scans show details of both external and internal anatomy that were not readily observable through visual inspection of the specimen itself. In addition, the scans enabled careful inspection of sutural relationships between bones that resulted in a number of unexpected findings, for example that *Pseudotherium* retains a large prefrontal bone and a vestigial postorbital bone. From the scans we also generated a number of animations of serial sections in all three orthogonal planes and enlarged 3D printouts that augmented our ability to understand the anatomy of the new taxon.

We also approximated the phylogenetic relationship of this new fossil using recently published data matrices designed to resolve relationships among non-mammalian cynodonts [23–25]. We note that little of the new anatomical detail revealed by the CT scans is reflected in this matrix, and that a broader comparative sample of CT scan data of relevant fossils needs to be made available before it will be useful or informative to build a more comprehensive matrix that reflects this new source of information on evolutionary variation in stem-mammals. For example, the scans showed large open spaces in the diploё around the braincase of the new taxon. We provisionally consider these large spaces autapomorphic of *Pseudotherium*, but without CT scans the condition in the other taxa of interest cannot be determined.

## Geological and paleontological settings

**Fig 1:**
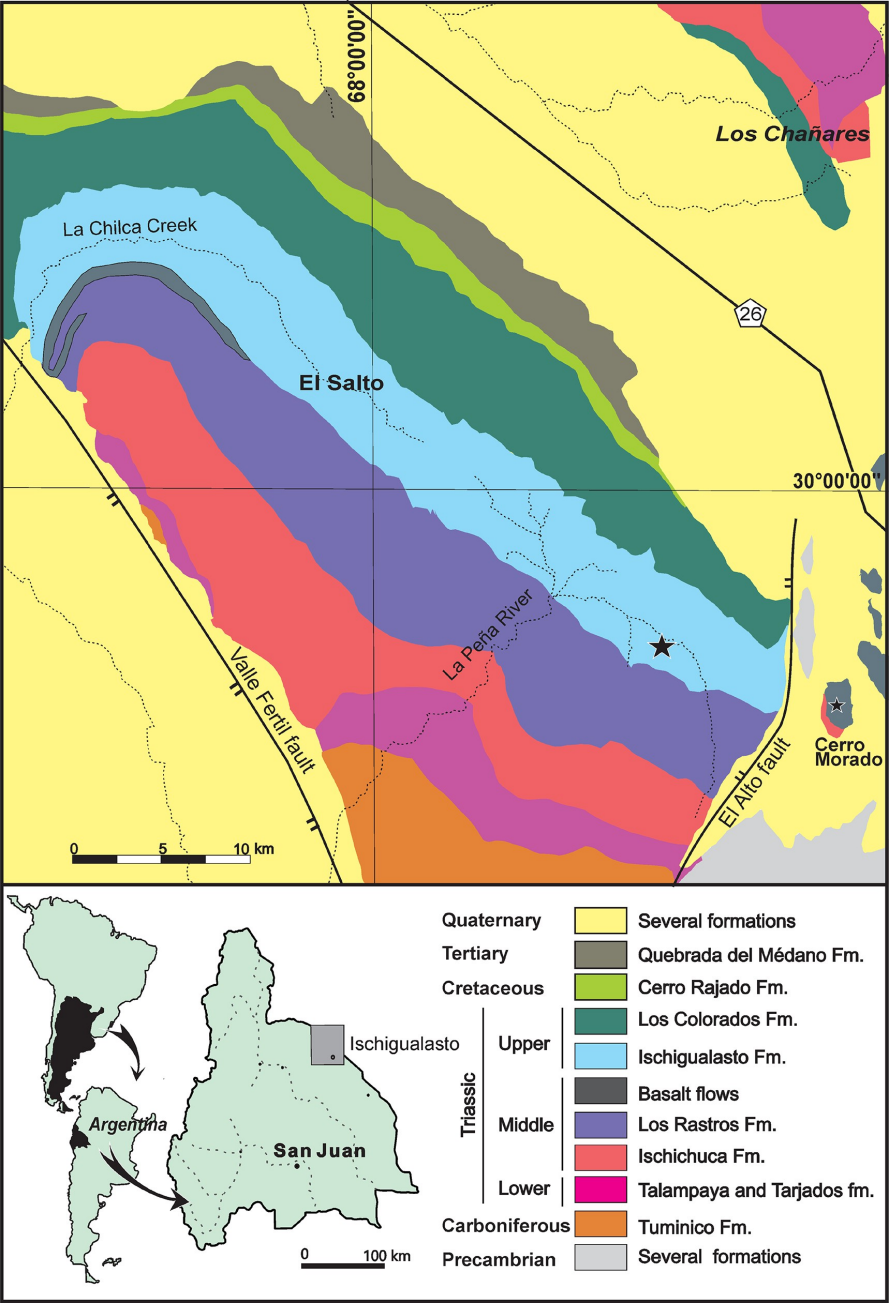
Geographic and geologic maps of the southern portion of the Ischigualasto-Villa Unión Basin (modified from [46] Martínez et al., 2012 [57]).

The holotype of the new taxon (PVSJ 882) was found in 2006 by RNM during a field trip to the Ischigualasto Formation carried out by the Instituto y Museo de Ciencias Naturales of the Universidad Nacional de San Juan. This nonmarine unit crops out in northwestern Argentina and forms part of the Ischigualasto-Villa Unión Basin (Fig 1). The Ischigualasto Formation comprises a sequence of fluvial channel sandstones with well-drained floodplain sandstones and mudstones. Interlayered volcanic ash layers above the base and below the top of the formation provide chronostratigraphic control and yielded ages of 231.4 ± 0.3 Ma and 225.9 ± 0.9 Ma [55,56].

The Ischigualasto Formation is divided into four members [58]: the La Peña (from the base to 40 m), the Cancha de Bochas (40 to 180 m), the Valle de la Luna (180 to 650 m) and the Quebrada de la Sal (650 to 700 m) members (Fig 1). The La Peña Member consists of multi-story channel sandstones and conglomerates covered by poorly-drained floodplain mudstones. The Cancha de Bochas Member is composed of thick, well-drained floodplain mudstones interbedded with high-sinuosity channel sandstones. The Valle de la Luna Member is mostly characterized by amalgamated high-sinuosity channels, abandoned channels and marsh deposits. Finally, the Quebrada de la Sal Member consists of tabular fluvial deposits.

The Ischigualasto Formation is divided from base to top into three abundance-based biozones [56]: the *Scaphonyx*-*Exaeretodon*-*Herrerasaurus* biozone; the *Exaeretodon* biozone; and the *Jachaleria* biozone. The *Scaphonyx*-*Exaeretodon*-*Herrerasaurus* biozone is characterized by a predominance of the rhynchosaur *Scaphonyx*, the cynodont *Exaeretodon*, and the dinosaur *Herrerasaurus*, but also includes the majority of known fossils and the highest taxonomic diversity. The *Exaeretodon* biozone is characterized by low diversity and high relative abundance of the cynodont *Exaeretodon*. The *Jachaleria* biozone is almost devoid of vertebrate fossils except for scarce specimens of the dicynodont *Jachaleria*.

The new specimen was found at the Valle Pintado locality, which is located in the upper levels of the La Peña Member and in the lower portion of the *Scaphonyx-Exaeretodon-Herrerasaurus* biozone. The material was found in a fossiliferous layer 40 m above the base of the Formation. To date, this is one of the most fossiliferous horizons known in the Ischigualasto Formation. Diverse and abundant fauna were recovered from the same level, including several specimens of the theropod dinosaur *Herrerasaurus*, the type specimen of the basal sauropodomorph dinosaur *Panphagia*, the only known specimen of lagerpetid dinosauromorph [57], plus various carnivorous and herbivorous cynodonts, rhynchosaurs, and pseudosuchian archosaurs.

## Materials and methods

### Computed tomography

Much of the superficial surface of the skull was exposed through manual preparation. Anatomical investigation of the interior utilize micro-computed tomography (μCT) [48–50,59], and from these scans 3D printouts of enlarged models of the specimen were made to augment and extend observation of its surficial anatomy. This approach complements earlier analyses using μCT to study various regions of the closely related brasilodontid *Brasilitherium riograndensis* [53,54,60], and the tritheledontid *Riograndia guaibensis* [61]. The resulting CT images of *Pseudotherium* are remarkably clear and interpretable thanks to a marked X-ray contrast between fossil bone and matrix.

The holotype of *Pseudotherium* (PVSJ 882) was scanned by Dr. Jessie Maisano at the University of Texas High-resolution X-ray Computed Tomography Facility (http://www.ctlab.geo.utexas.edu) on November 11, 2013, using its Xradia microXCT 400 Scanner. Owing to the length of the specimen (69 mm), it was scanned in two parts, each of which consisted of a single rotation using cone-beam data acquisition, and the two halves were stitched together using an Xradia software plugin application. The entire dataset was reconstructed as a total of 1733 coronal CT slices exported as 16bit TIFF files that measure 1024 x 1008 pixels. Voxels are cubic and measure 44.21 microns along each orthogonal axis.

Scanning parameters are as follows: Xradia 0.7X objective, 110kV, 10W, 2s acquisition time, detector 50.5 mm, source -96.8 mm, XYZ [-2096, 39633, -111], camera bin 1, angles ±180, 1081 views, 1 mm CaF_2_ filter, dithering. End reference (60 frames, 1.5s each). Reconstructed with center shift -6.5, beam hardening 0.1, theta 0, byte scaling [-20, 500], binning 1, recon filter smooth (kernel size = 0.5).

**Fig 2:**
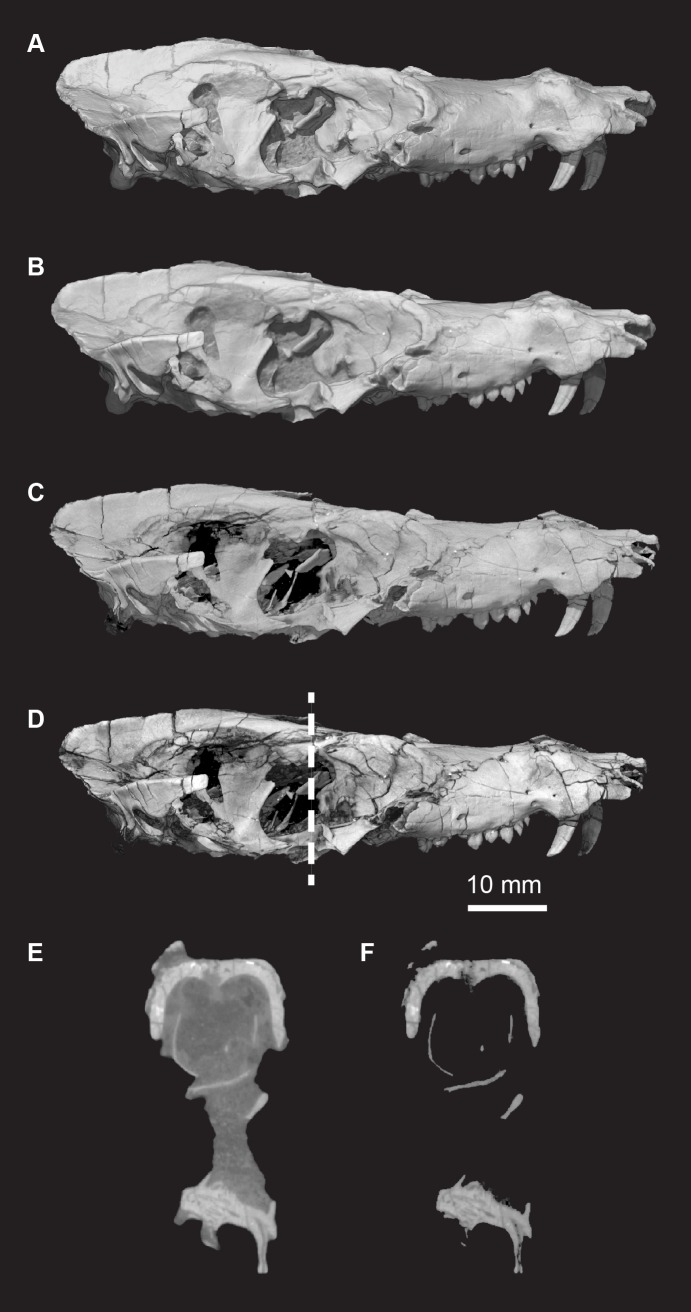
*Pseudotherium argentinus*, a comparison of image processing methods based on μCT scans. (A) Isosurface rendering; (B) volume rendering, scattering algorithm, no digital matrix removal; (C) volume rendering, HQ scattering algorithm, digital matrix removal; (D) volume rendering, Phong algorithm, digital matrix removal; (E) cross section, no digital matrix removal; (F) cross section, digital matrix removal. Dashed line indicates position of cross sections (E) and (F).

The CT data were processed using VGStudio MAX version 2.1 software to generate 3D volumetric renderings to produce supplemental animations of the skull rotating about each orthogonal axis, and movies through slice stacks. Volumetric models were generated using the HQ Scatter algorithm unless otherwise noted. Isosurface renderings are more ubiquitous in the literature, but they only present a thresholded surface of a scanned item which reduced their anatomical informativeness compared to volumetric reconstructions [(49)]. Because the fossil bone in the new taxon is significantly more attenuating to X-rays than the surrounding matrix, a histogram adjustment was applied to digitally render matrix voxels as transparent in some of the illustrations below (e.g., Fig 2), enabling visualization of bones completely encased by matrix. Still images were exported from VGStudio, cropped in Adobe Photoshop, and labeled in Adobe Illustrator. Fig 2 illustrates the difference in information conveyed between a volume rendering of the new taxon that was digitally filtered (prepared), and an isosurface rendering. Although the isosurface rendering appears sharper and less cloudy, the high-quality scatter volume rendering reveals more fractures and sutures. Fractures and sutures are even clearer with Phong volume rendering, though they may be a distraction from other anatomical features. Grayscale histogram-based digital preparation was able to expose deep elements, such as the orbitosphenoid, and although some elements, such as the occipital condyles (Fig 2C and 2D), appear to have been lost after digital preparation, examination of the specimen reveals that the cortical bone of the occipital condyles was eroded away, leaving only remains of spongy bone. Cross sectional slices (Fig 2E and 2F) illustrate how little fossil bone is lost in the digital preparation threshold selected for these CT images of *Pseudotherium* and even thin, wispy elements can be seen in the nasopharyngeal and endocranial areas.

### Institutional abbreviations

**PVSJ**, Instituto y Museo de Ciencias Naturales, San Juan, Argentina; **UFRGS-PV**, Laboratório do Setor de Paleovertebrados, Instituto de Geosciencias, Universidade Federal do Rio Grande do Sul, Porto Alegre, Brazil.

### Nomenclature

We follow the nomenclatural recommendations based on the Phylocode that are discussed at length elsewhere [1,62–66] and employ taxonomic names that are detailed in *Phylonyms*, *the Companion Volume to the Phylocode* (Cantino et al., *in press*). The name Mammalia is used in reference to the crown clade [1] (Rowe *in press* A). The name Mammaliaformes [1] is used in reference to the clade containing the last common ancestor shared by Mammalia and *Morganucodon oehleri* (Rowe, *in press* B) and all of its descendants. And Mammaliamorpha [1] is used in reference to a slightly more inclusive clade containing the last common ancestor shared by Mammalia and *Tritylodon longaevus* and all of that ancestor’s descendants [1] (Rowe *in press* C). Herein we use the name Probainognathia in reference to the clade containing the last common ancestor of Mammalia and *Probainognathus jenseni* and all of its descendants [67].

### Nomenclatural Acts

The electronic edition of this article conforms to the requirements of the amended International Code of Zoological Nomenclature, and hence the new names contained herein are available under that Code from the electronic edition of this article. This published work and the nomenclatural acts it contains have been registered in ZooBank, the online registration system for the ICZN. The ZooBank LSIDs (Life Science Identifiers) can be resolved and the associated information viewed through any standard web browser by appending the LSID to the prefix "http://zoobank.org/". The LSID for this publication is: urn:lsid:zoobank.org:pub:7BFFD2FA-6E1A-4630-BEAE-56AA2D7B794B. The electronic edition of this work was published in a journal with an ISSN, and has been archived and is available from the following digital repositories: PubMed Central, LOCKSS.

### Supplemental data

Supplemental data will be made available on www.DigiMorph.org. They include the original CT dataset, serial section movies, 3D movies in X- Y- and Z- axis rotation; serial section movies in coronal, sagittal, and horizontal slice planes; and an STL file of the holotype.

## Results

### Systematic paleontology

Therapsida Broom, 1905

    Cyndontia Owen, 1861

        Probainognathia Hopson, 1990

***Pseudotherium*** gen. nov. [urn:lsid:zoobank.org:act:1FC665C8-53D4-4325-9E4E-2C9574924913]

**Etymology:** *Pseudo* (L.) for false, plus Greek *therios* (G.) for wild beast, a mammal

**Type and only known species:** *Pseudotherium argentinus* sp. nov.

**Diagnosis:** As for the species.

P. *argentinus*, sp. nov.

**Etymology:** Species name, *argentinus*, in reference to its provenance.

*Pseudotherium argentinus* sp. nov. [urn:lsid:zoobank.org:act:7F78A17F-7122-43BF-AF92-FC4D64E20B4A]

**Holotype**: Instituto y Museo de Ciencias Naturales, San Juan, Argentina, PVSJ 882 (Figs 2–30), an isolated skull that is missing the mandibles, most of the premaxillae, zygomatic arches, and quadrates (Figs 3 and 4). One incomplete stapes and one quadratojugal are preserved.

**Fig 3:**
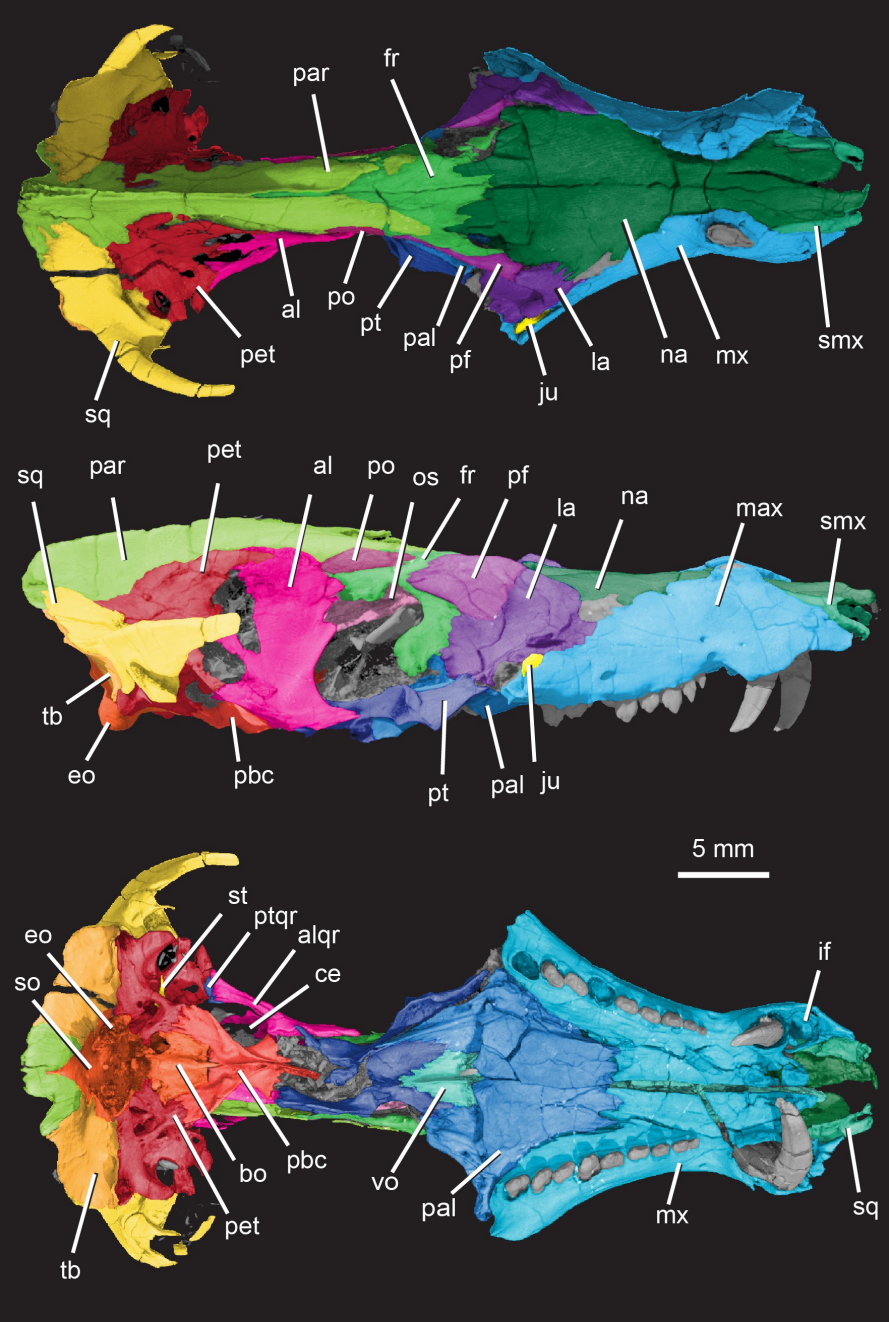
*Pseudotherium argentinus*, digitally colored 3D volume renderings of the holotype. Skull in dorsal (top), right lateral (middle), and ventral (bottom) views. Abbreviations: al, alisphenoid; alqr, quadrate ramus of alisphenoid; bo, basioccipital; ce, cavum epiptericum; eo, exoccipital; fr, frontal; if, incisive fossa; ju, jugal; la, lacrimal; mx, maxilla; na, nasal; os, orbitosphenoid; pal, palatine; par, parietal pbc, parabasisphenoid complex; pet, petrosal (= periotic); pf, prefrontal; po, postorbital; pt, pterygoid; ptqr, quadrate ramus of pterygoid; smx, septomaxilla; so, supraoccipital; sq, squamosal; st, stapes; tb, tabular; vo, vomer.

**Fig 4:**
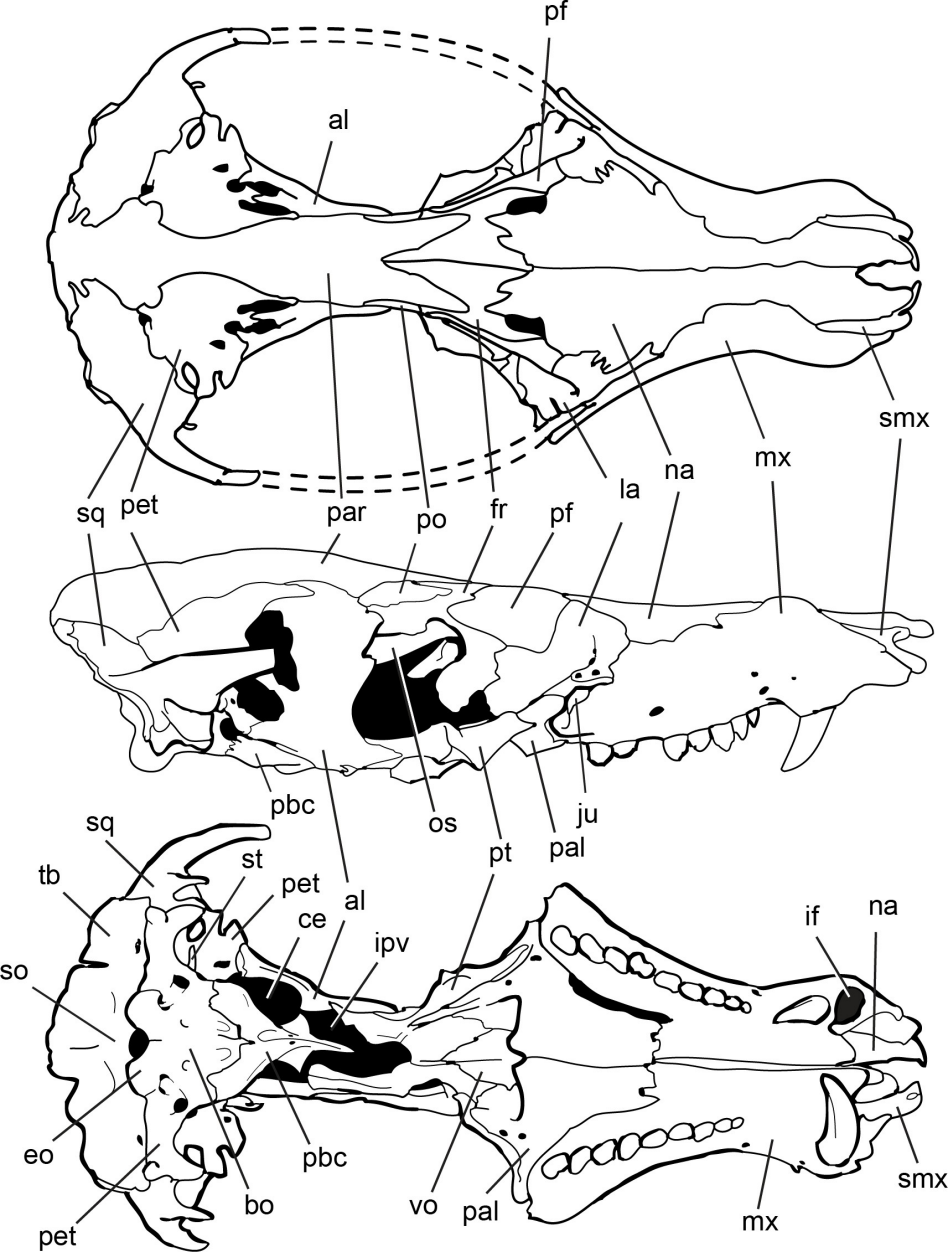
Line drawings of *Pseudotherium argentinus* holotype. (Top) Reconstructive drawing of fossil in dorsal view. Zygomatic arches depicted with dashed lines. Major cracks in the fossil specimen were avoided in the drawing. The more complete right half of the fossil was mirrored to reconstruct the less complete left half. Drawings of fossil in its original, preserved condition, depicted in right lateral (middle) and ventral (bottom) views. Abbreviations: al, alisphenoid; alqr, quadrate ramus of alisphenoid; bo, basioccipital; ce, cavum epiptericum; eo, exoccipital; fr, frontal; if, incisive fossa; ipv: interpterygoid vacuity; ju, jugal; la, lacrimal; mx, maxilla; na, nasal; os, orbitosphenoid; pal, palatine; par, parietal pbc, parabasisphenoid complex; pet, petrosal (= periotic); pf, prefrontal; po, postorbital; pt, pterygoid; ptqr, quadrate ramus of pterygoid; smx, septomaxilla; so, supraoccipital; sq, squamosal; st, stapes; tb, tabular; vo, vomer.

**Fig 5:**
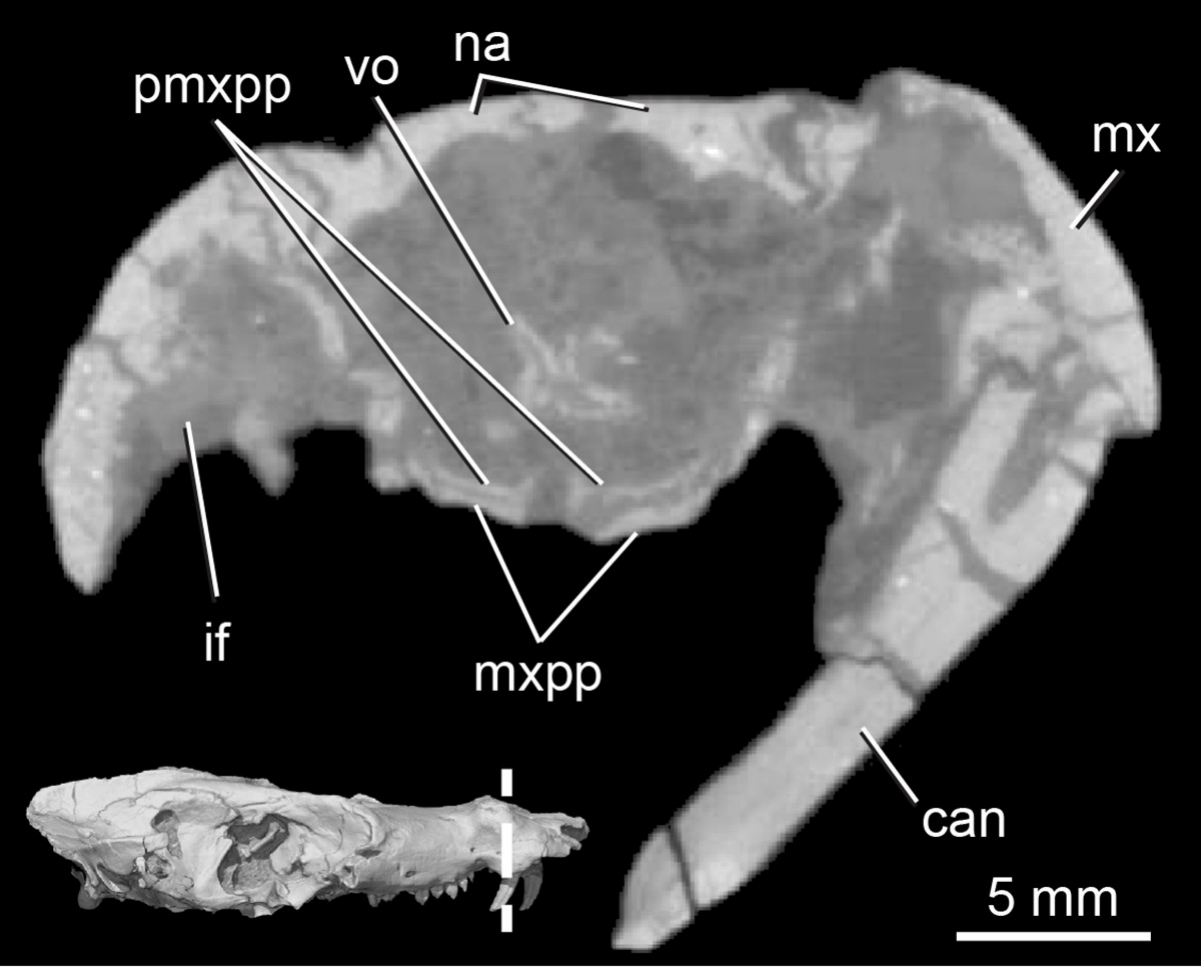
*Pseudotherium argentinus*, cross-section through the snout, showing the palatine processes of the premaxilla. Abbreviations: can, canine; if, incisive fossa for lower canine; mx, maxilla; mxpp, palatine process of maxilla; na, nasal, pmxpp, palatine process premaxilla; vo, vomer.

**Fig 6:**
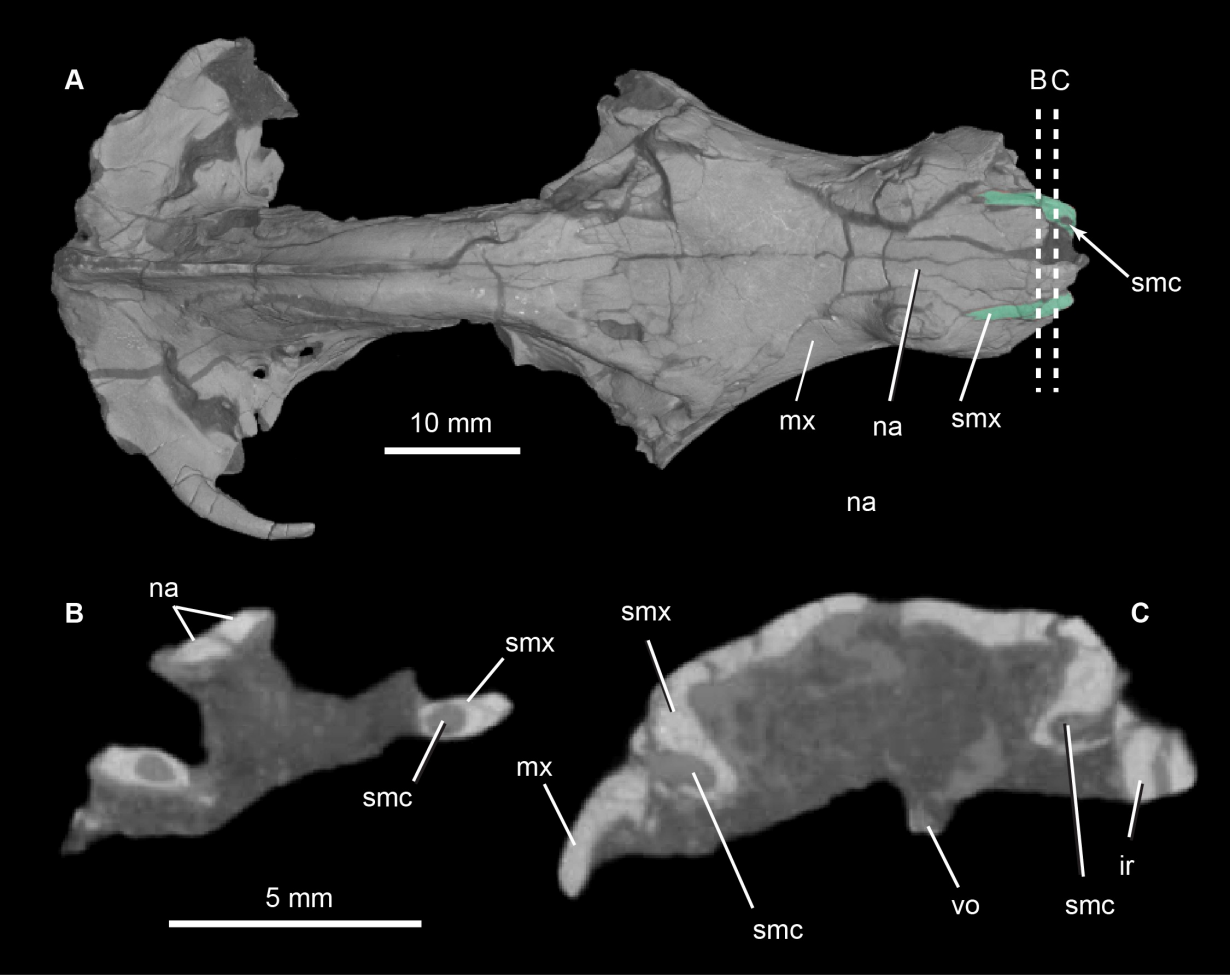
*Pseudotherium argentinus*, the septomaxillary canal. (A) 3D volumetric rendering of dorsal view of skull showing septomaxillae in aqua tint (A), and dashed lines that indicate positions of cross sectional CT image slices (B) and (C). Abbreviations: ir, broken incisor root; mx, maxilla; na, nasal; smc, septomaxillary canal; smx, septomaxilla, vo, vomer.

**Fig 7:**
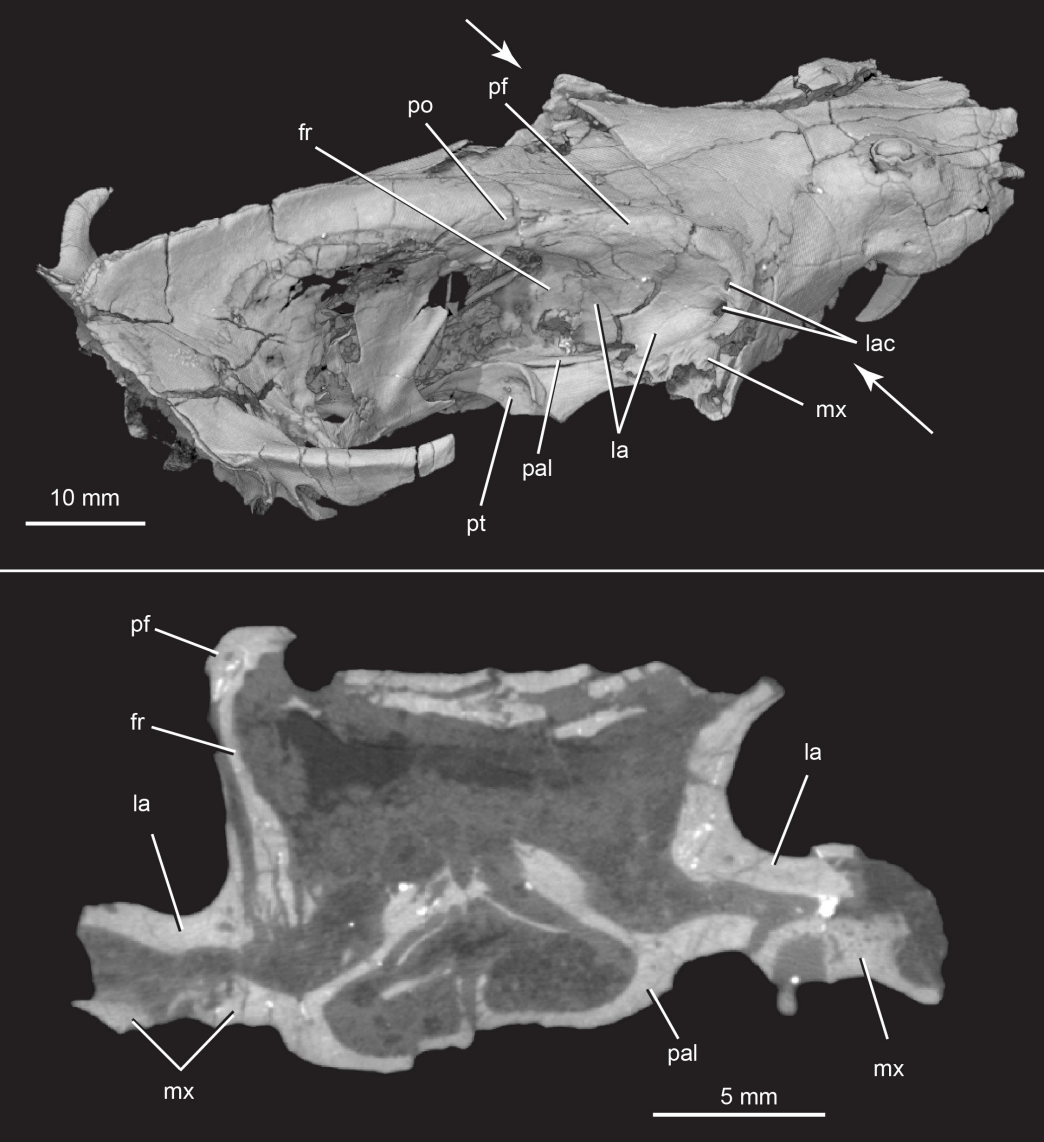
*Pseudotherium argentinus*, wall and floor of the orbit. (A) Oblique posteriodorsal view. White arrows indicate plane of coronal slice (B). Abbreviations: fr, frontal; la, lacrimal; lac, lacrimal canal; mx, maxilla; pal, palatine; pf, prefrontal; po, postorbital; pt, pterygoid.

**Fig 8:**
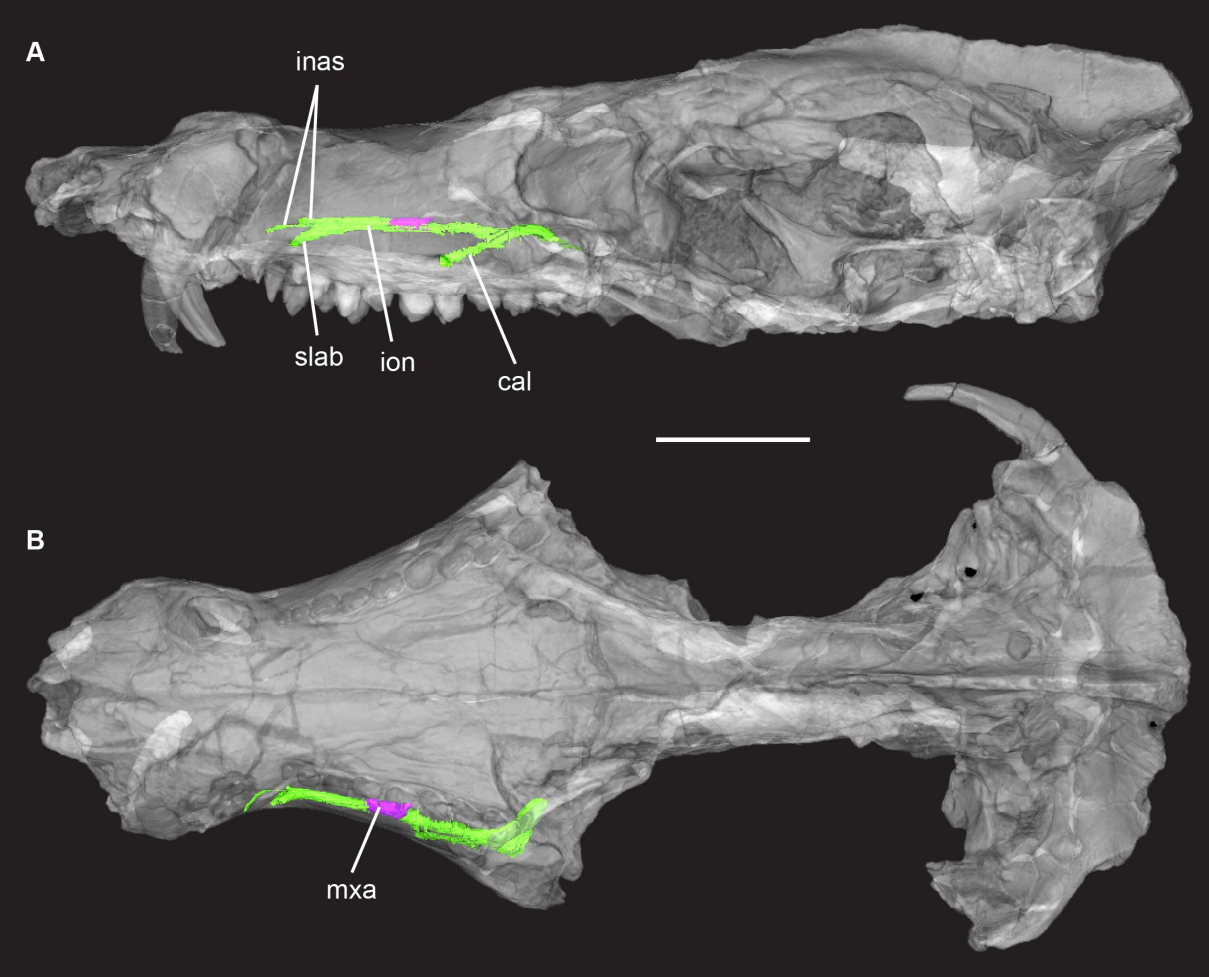
*Pseudotherium argentinus*, isosurface rendering of maxillary nerve. (A) skull in left lateral and (B) dorsal views. Maxillary nerve = green. Maxillary antrum = purple. Skull rendered semitransparent to view canal and antrum in situ. Abbreviations: cal, caudal alveolar ramus; inas, internal nasal rami of infraorbital nerve; ion, infraorbital nerve; mxa, maxillary antrum; slab, supralabial ramus of infraorbital nerve.

**Fig 9:**
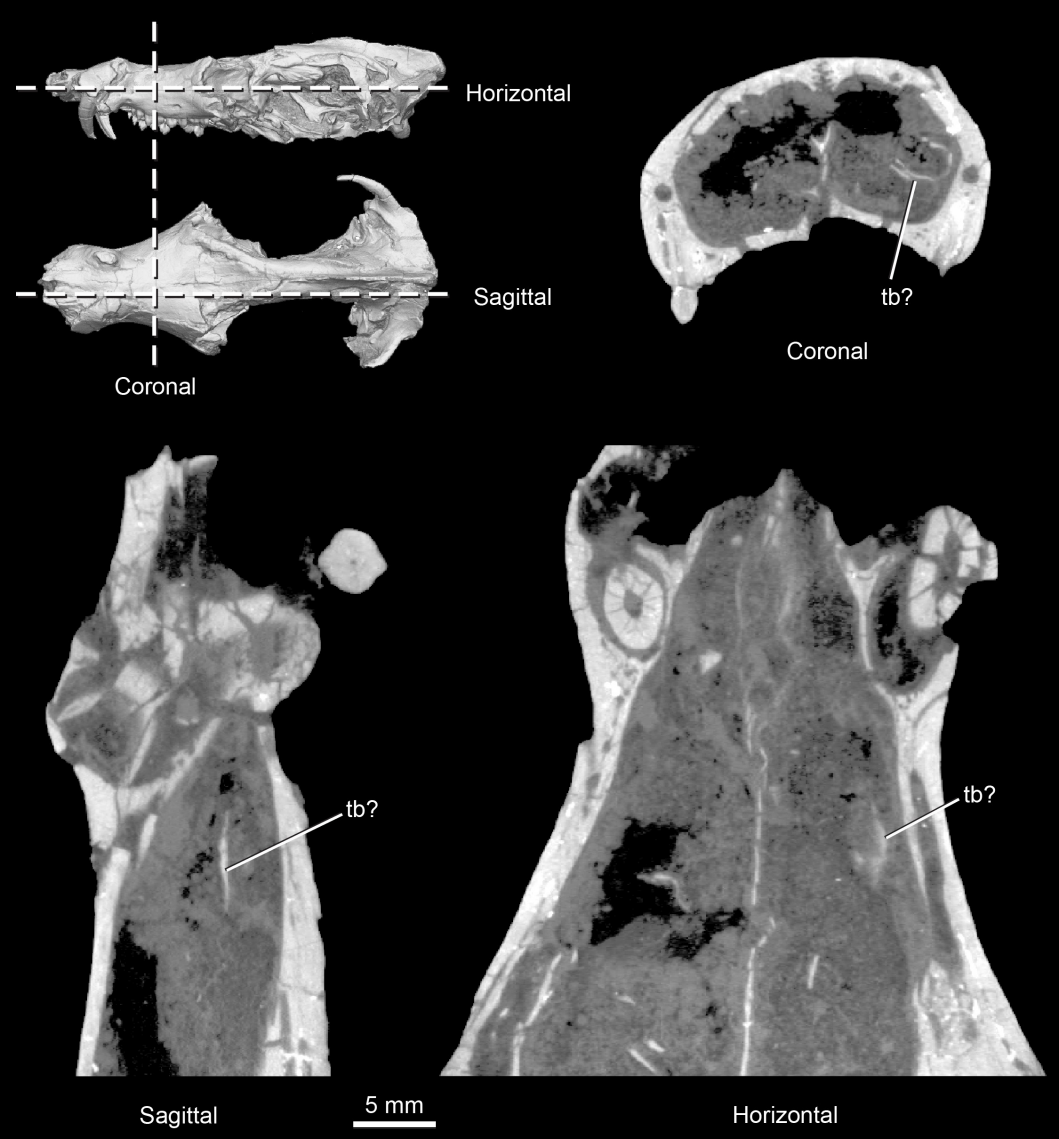
*Pseudotherium argentinus*, thin bone fragments of the nasal capsule. One may represent a turbinal (tb?), but others are probably exfoliated fragments of the nasopharyngeal wall.

**Fig 10:**
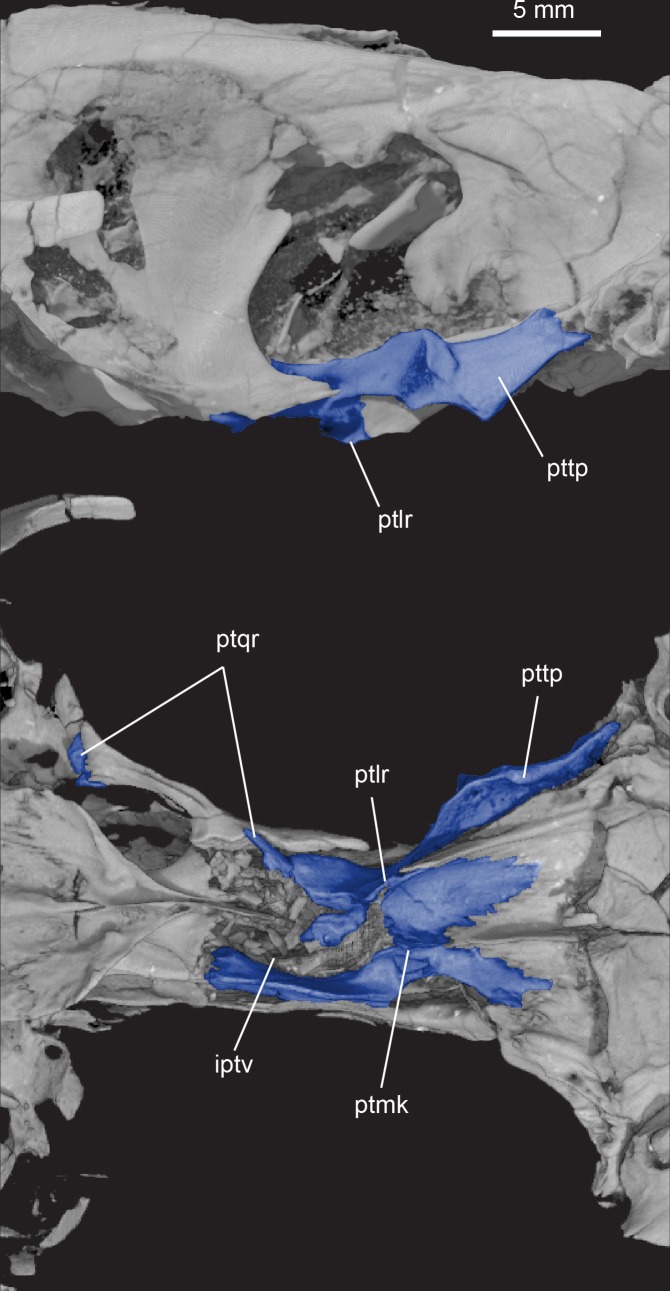
***Pseudotherium argentinus*, pterygoid (blue) in right lateral (top) and ventral (bottom) views.** Abbreviations: iptv, interpterygoid vacuity; ptlr, pterygoid lateral ridge; ptmk, pterygoid median keel; ptqr, quadrate ramus of pterygoid; pttp, pterygoid transverse process.

**Fig 11:**
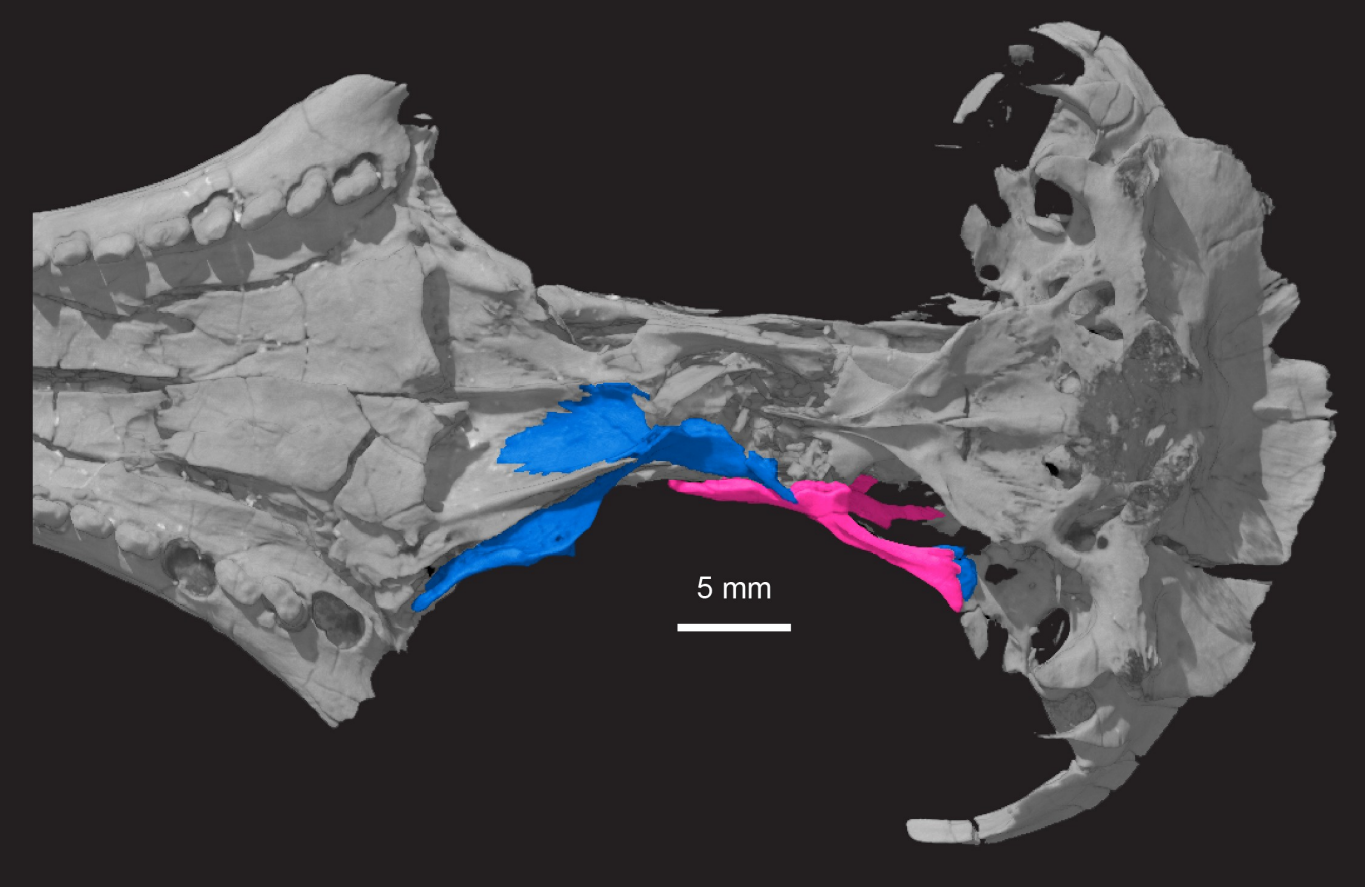
***Pseudotherium argentinus*, ventral view of skull showing quadrate processes of right alisphenoid (pink) and right pterygoid (blue).** The quadrate process of the pterygoid is broken at its base but its terminal end is preserved at the distal end of the quadrate process of the alisphenoid.

**Fig 12:**
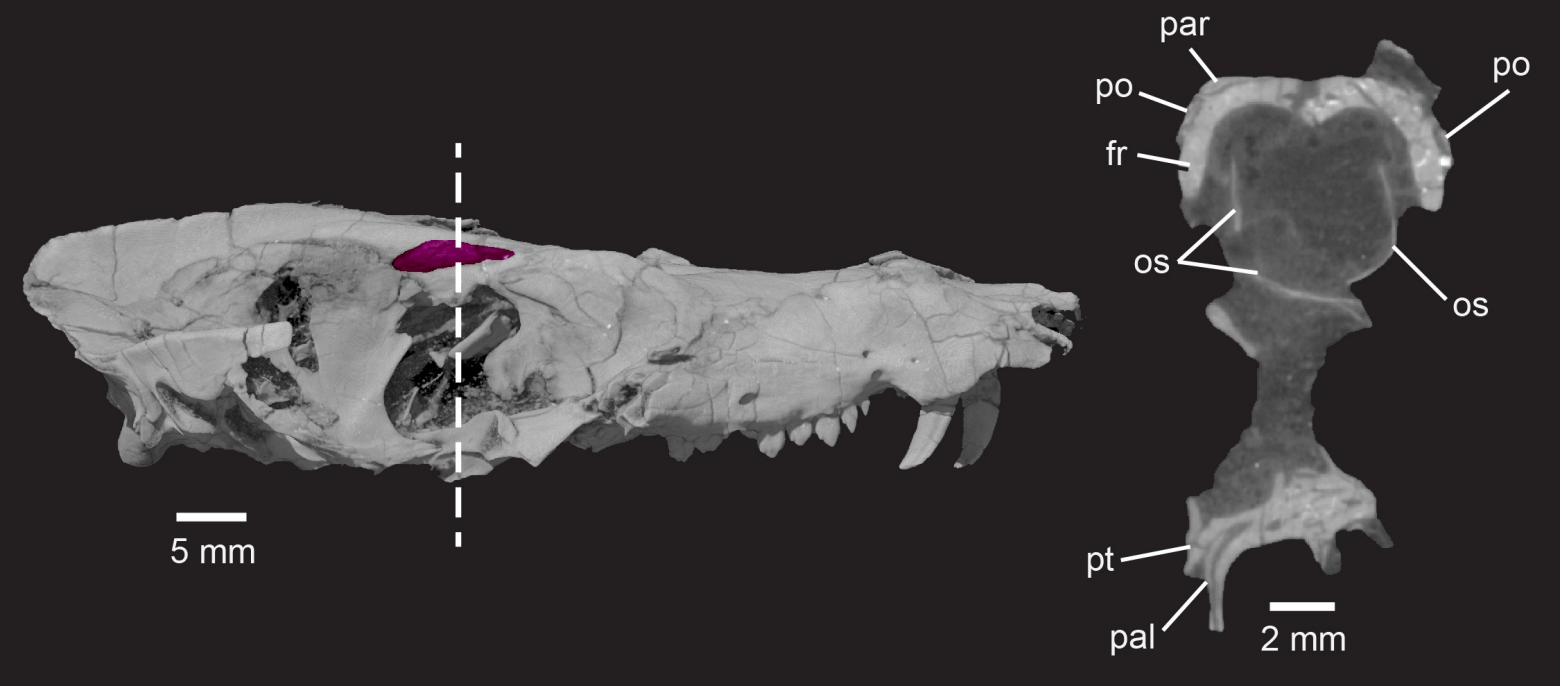
*Pseudotherium argentinus*, right lateral view of skull showing the postorbital in *Pseudotherium*. (Left) Right postorbital highlighted in magenta. (Right) Cross section through orbital region illustrating how a sliver of frontal is wedged between dorsal parietal sand ventral postorbitals. Dotted line indicates position of cross sectional slice. Abbreviations: fr, frontal; par, parietal; po, postorbital.

**Fig 13:**
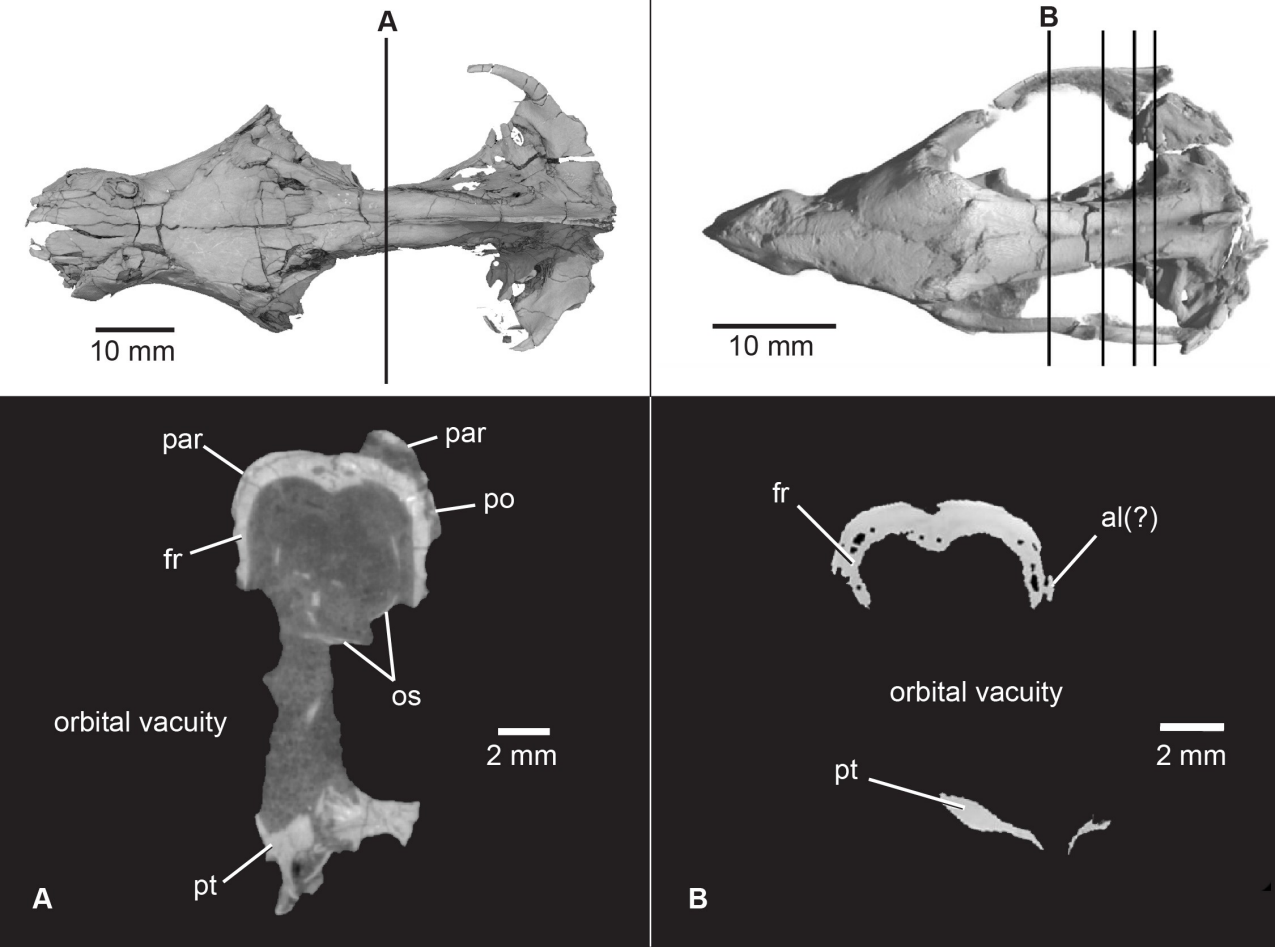
Comparison of orbital vacuities in *Pseudotherium* and *Brasilitherium riograndensis*. The ventral limit of the endocranium in *Pseudotherium* (A) is dorsal to the orbital vacuity and lined by ossified orbitosphenoids. The orbitosphenoids are missing in *Brasilitherium* (B) but were likely present, suggesting a shallower endocast than previously hypothesized. *Brasilitherium riograndensis* CT images from Rodrigues et al., ([60]: Fig 3). Abbreviations: al, alisphenoid; fr, frontal; os, orbitosphenoid; par, parietal; po, postorbital; pt, pterygoid.

**Fig 14:**
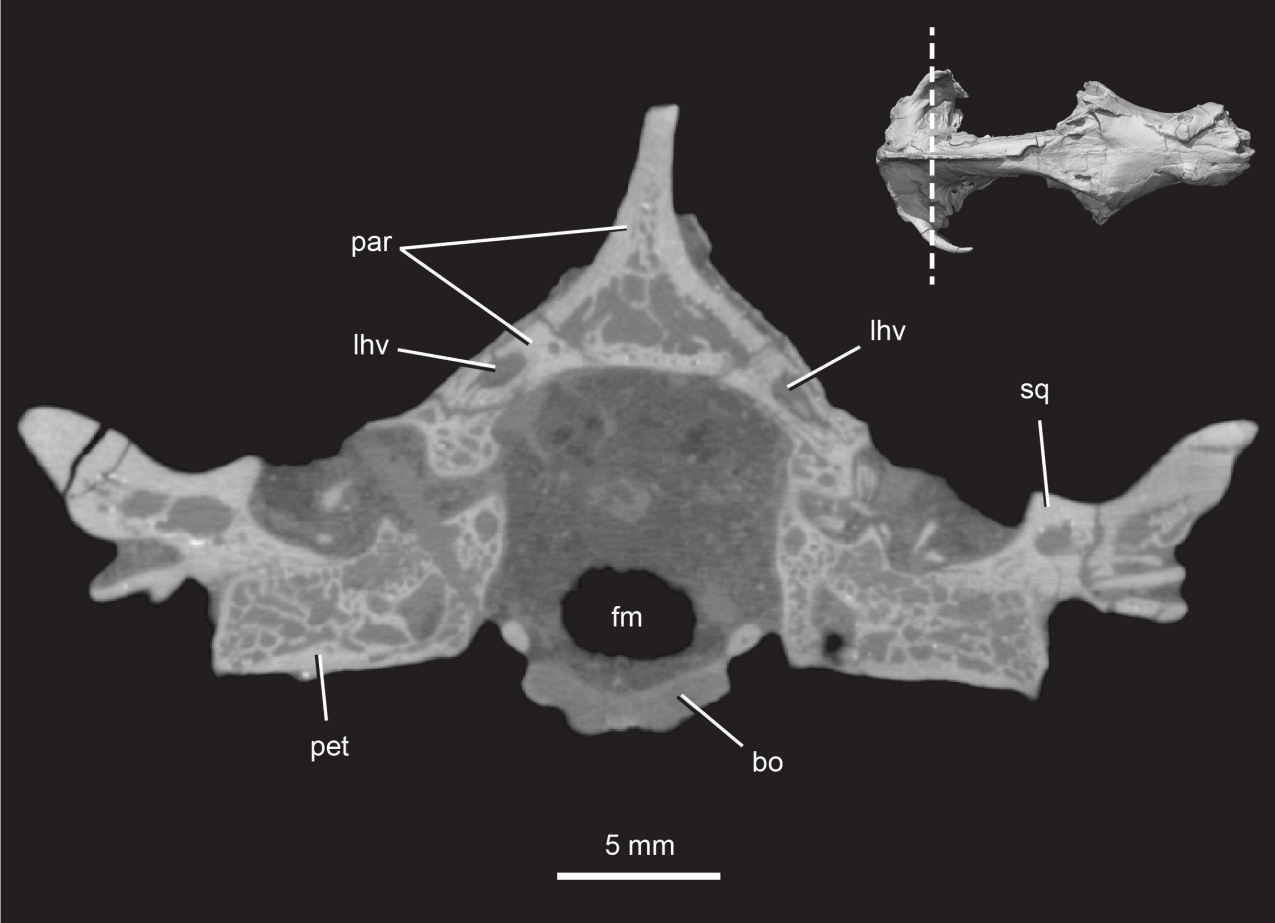
*Pseudotherium argentinus*, CT cross section through the back of the skull of the skull. Note the extensive hollow spaces in the parietal, petrosal, and squamosal. Abbreviations: bo, basioccipital; fm, foramen magnum; lhv, lateral head vein; par, parietal; pet, petrosal; sq, squamosal.

**Fig 15:**
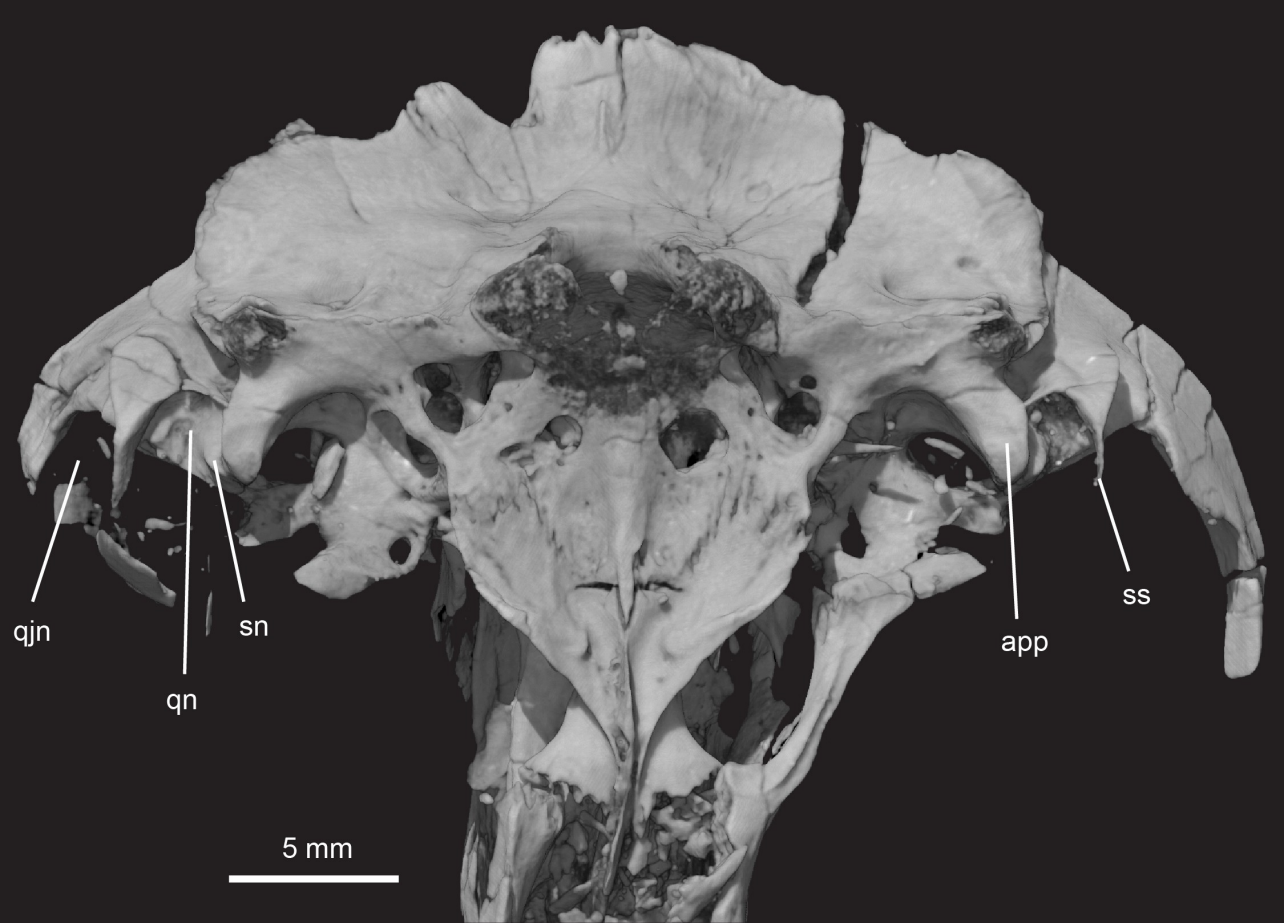
*Pseudotherium argentinus*, ventral view showing notches in temporal process of squamosal. The quadrate notch (medial) and the quadratojugal notch (lateral) are divided by a hook-shaped squamosal septum. A squamosal notch is present within the quadratojugal notch and abuts the anterior process of the bifurcated paroccipital process. The squamosal notch does not completely cover the lateral face of the anterior paroccipital process. Abbreviations: app, anterior paroccipital process; qn, quadratojugal notch; qjn, quadratojugal notch; sn, squamosal notch; ss, squamosal septum.

**Fig 16:**
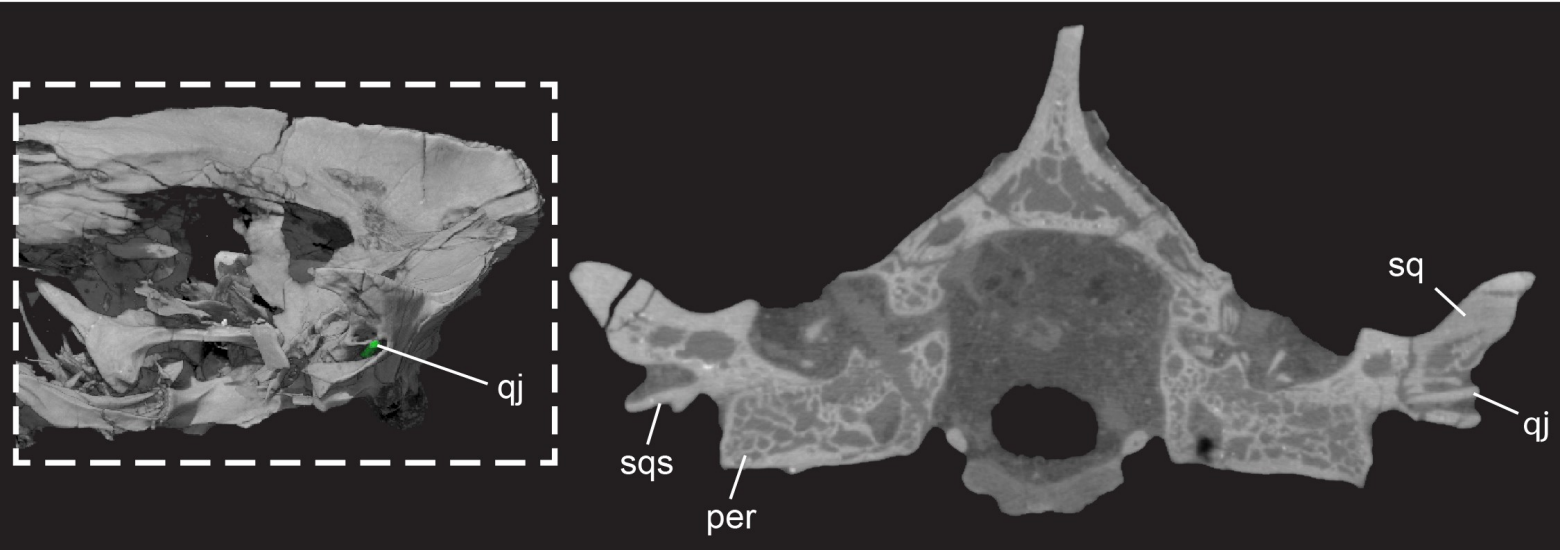
*Pseudotherium argentinus*, the quadratojugal. (Left) Fragment of right quadratojugal (qj), green, in quadratojugal notch. (Right) Cross section through back of skull illustrating the quadratojugal inserted into quadratojugal notch in the squamosal flange. Abbreviations: qj, quadratojugal; qjn, quadratojugal notch; sq, squamosal; sqs, squamosal septum.

**Fig 17:**
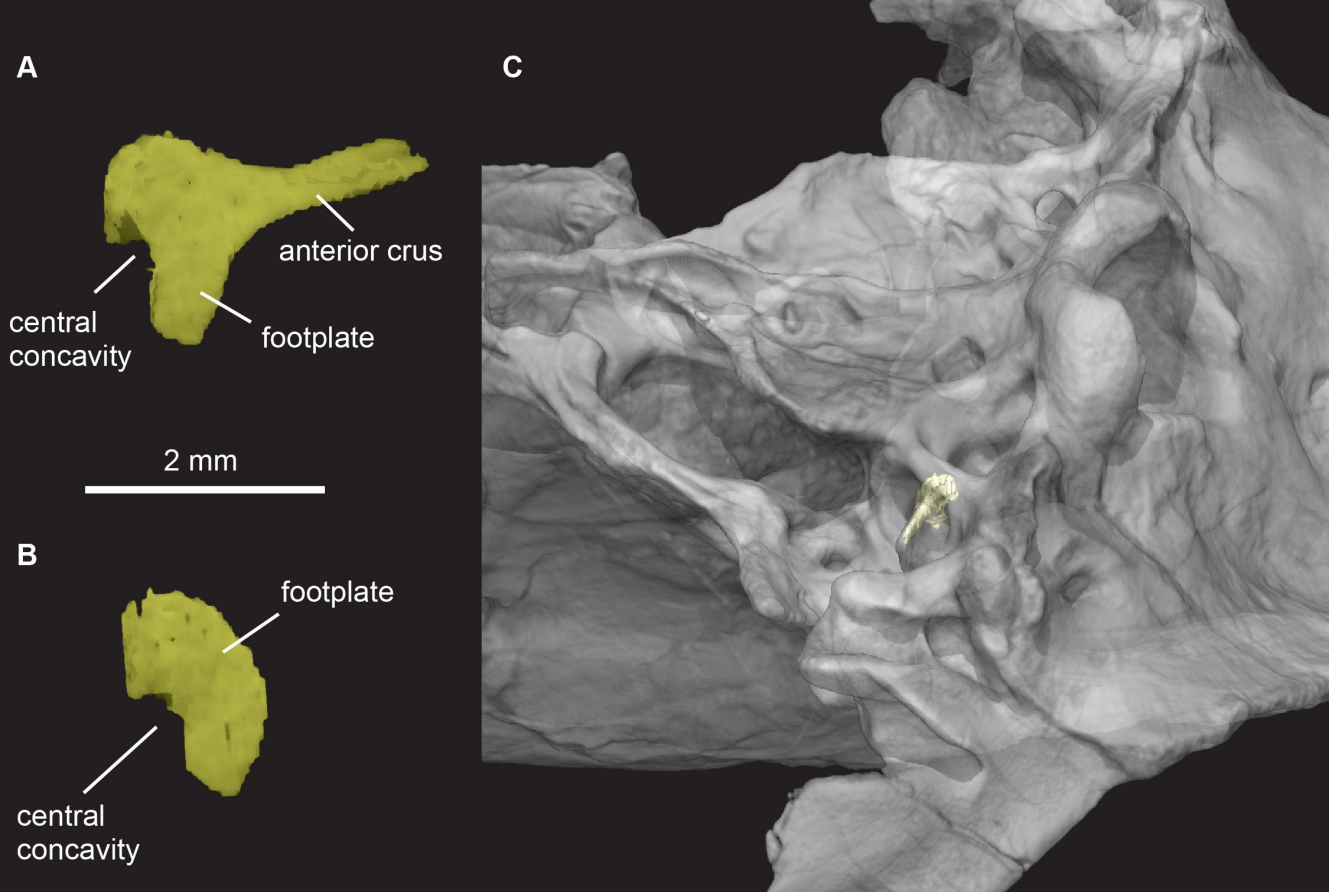
*Pseudotherium argentinus*, incomplete right stapes of *Pseudotherium*. (A) Anteromedial view of stapes, (B) medial view of footplate of stapes, and (C) semitransparent isosurface render of stapes in situ and skull in oblique-ventral view.

**Fig 18:**
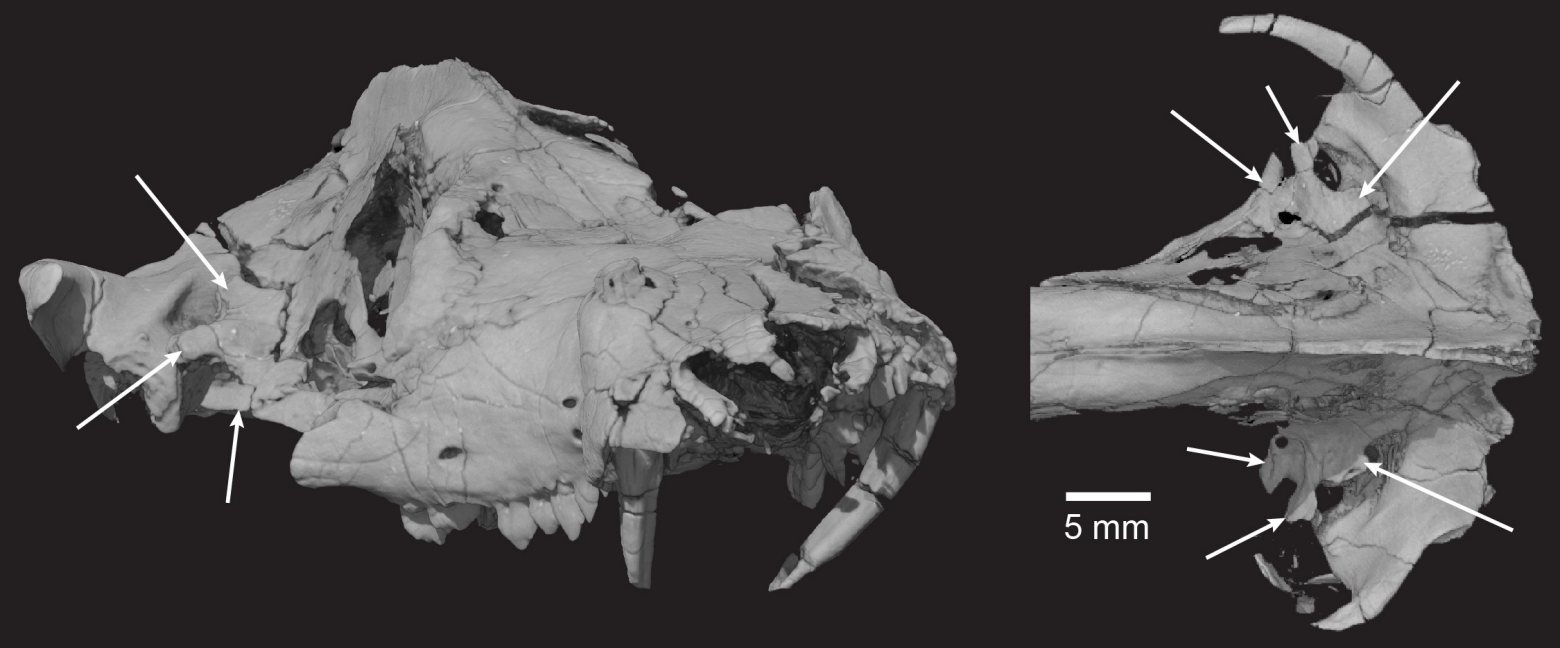
***Pseudotherium argentinus*, lateral flange of the petrosal right anterolateral (left) and dorsal (right) views.** The lateral flange (indicated with arrows) is broad and has a slightly vertical slant. The lateral margin of the lateral flange bears a notch which may be apomorphic of *Pseudotherium argentinus*.

**Fig 19:**
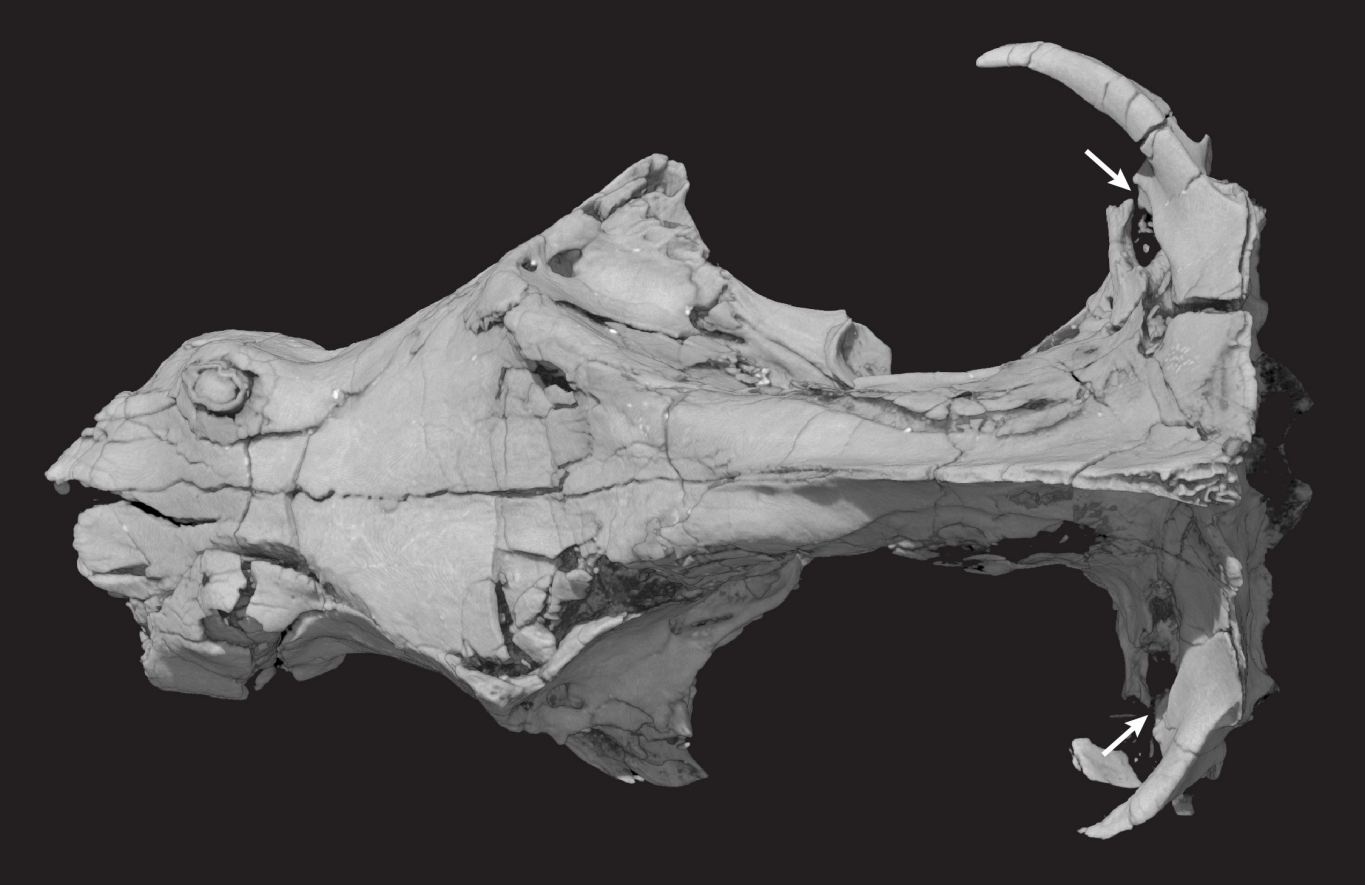
*Pseudotherium argentinus* skull in posterodorsal view illustrating open pterygoparoccipital foramina (indicated by arrows). Each pterygoparoccipital foramen is almost entirely enclosed by the lateral flange of the petrosal (periotic) anteriorly and the squamosal posteriorly. The lateral flange and the squamosal do not contact, so that pterygoparoccipital foramen is laterally open. Because each foramen is open to a similar extent, this is not likely to be an artifact of post-mortem deformation.

**Fig 20:**
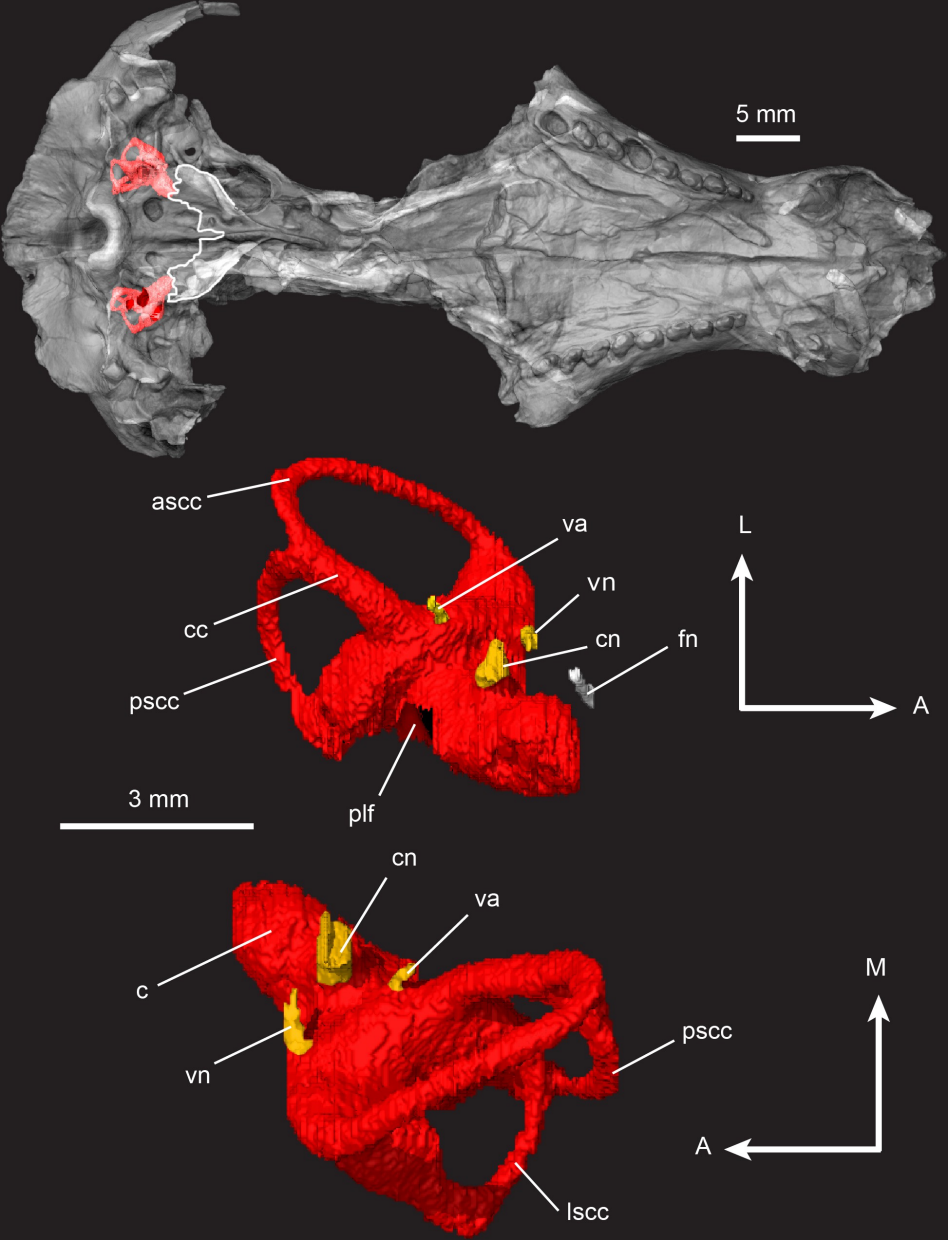
*Pseudotherium argentinus*, inner ear volume. (Top) Ventral view of inner ear endocranial space in situ with a semitransparent isosurface model of skull. White tracings outline the parasphenoid alae and posterior border of basisphenoid. (Middle) Left inner ear volume in dorsal view. (Bottom) Left inner ear volume in ventral view. Abbreviations: ascc, anterior semicircular canal; c, cochlea; cc, common crus; cn, cochlear nerve (VIII); fn, facial nerve (VII); lscc, lateral semicircular canal; plf, perilymphatic foramen; pscc, posterior semicircular canal; va, vestibular aqueduct; vn, vestibular nerve (VIII). Arrow legend key: A = anterior, L = lateral, M = medial.

**Fig 21:**
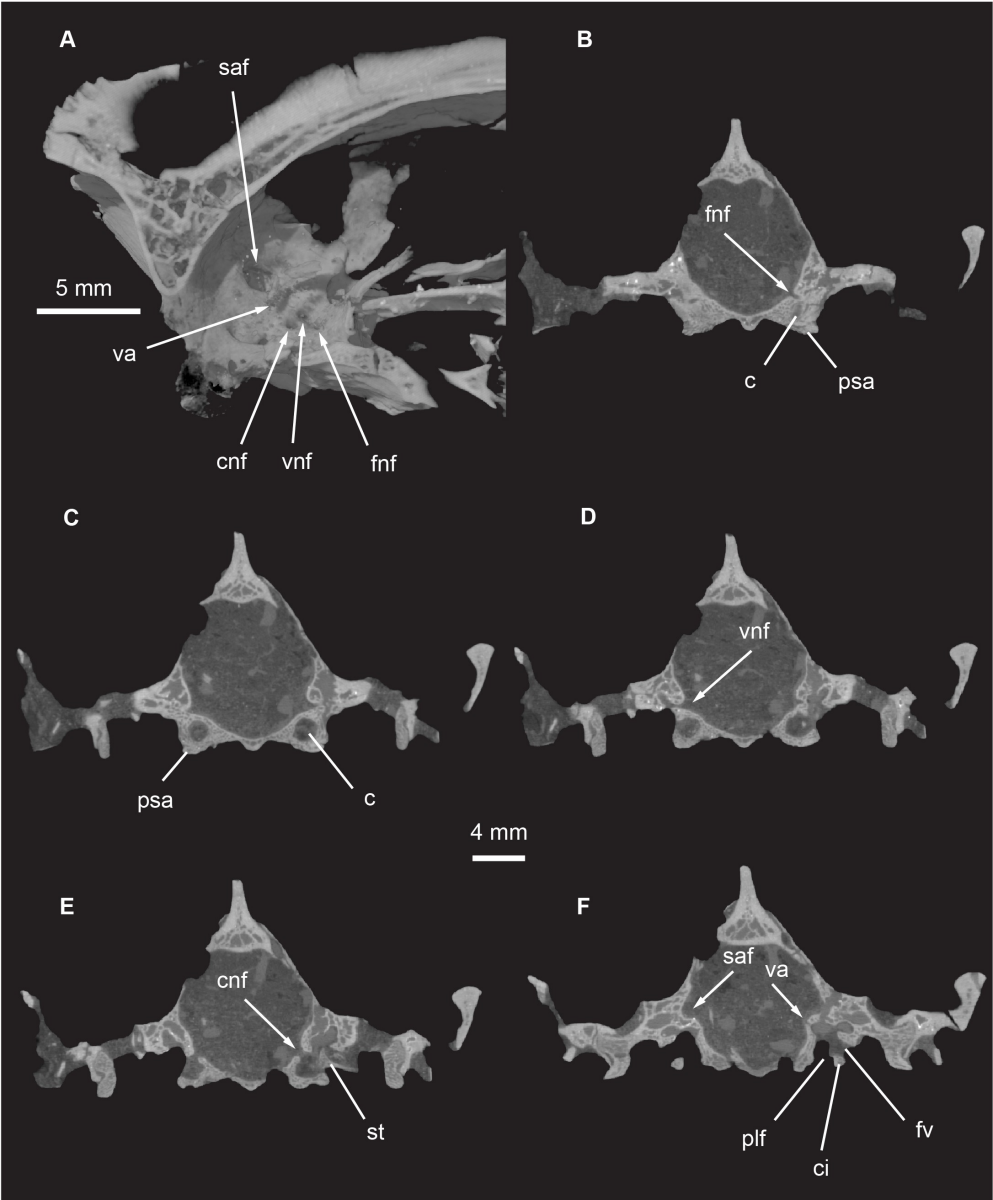
*Pseudotherium argentinus*, petrosal (periotic) in medial and cross sectional views. (A) Dynamic cutaway illustrating medial aspect of petrosal and its associated foramina and fossa. (B) Cross section through facial nerve foramen. (C) Cross section through vestibular nerve foramen. (D) Cross section through cochlear nerve entrance. (E) Cross section through vestibular aqueduct and subarcuate fossa. Abbreviations: c, cochlea; ci, crista interfenestralis; cnf, foramen for cochlear nerve; fnf, foramen for facial nerve; fv, foramen vestibuli; plf, parilymphatic foramen; psa, parasphenoid ala; saf, subarcuate fossa; st, stapes; va, vestibular aqueduct; vnf, foramen for vestibular nerve.

**Fig 22:**
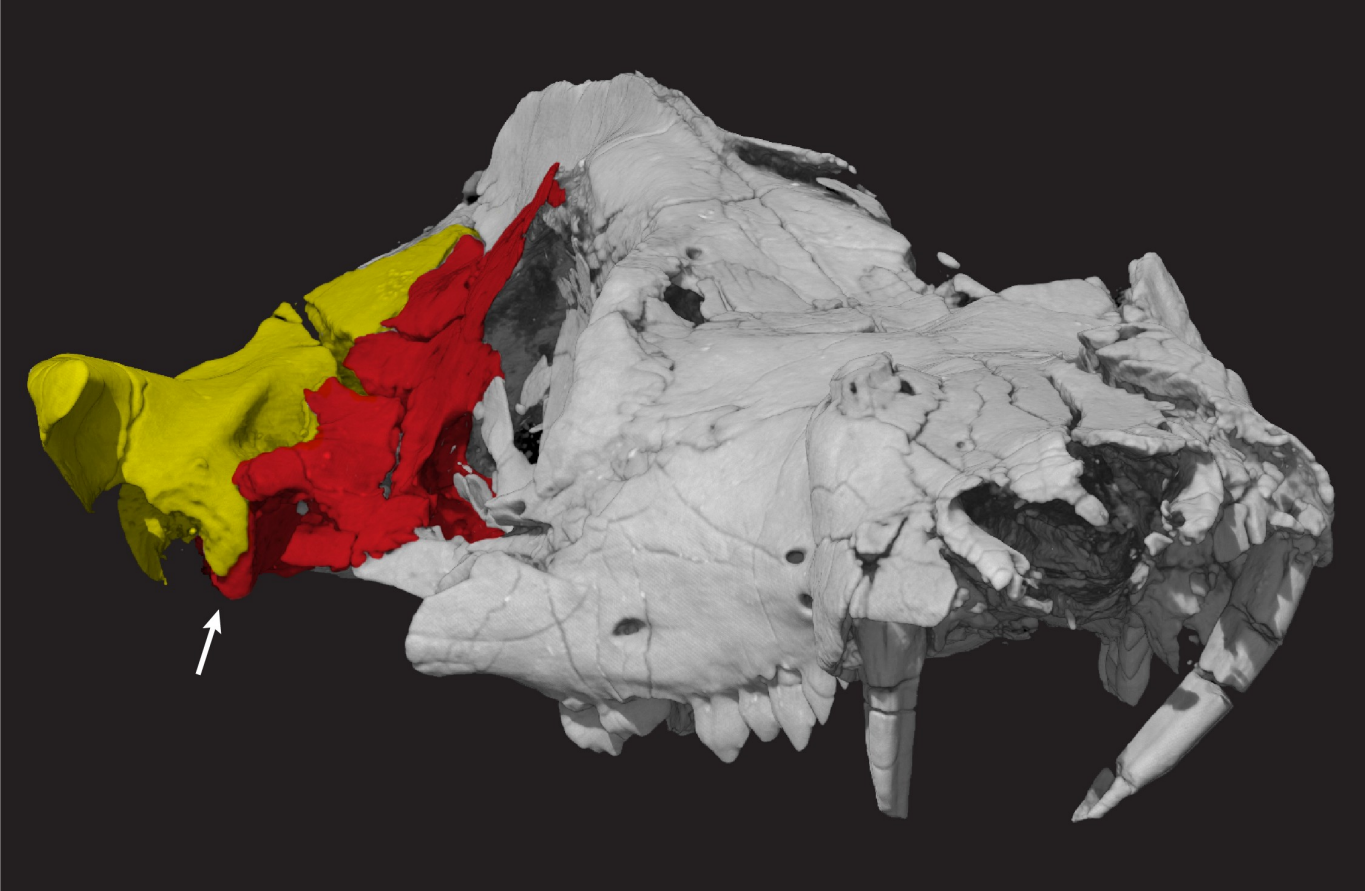
*Pseudotherium argentinus* in anterolateral view illustrating dorsal recession of squamosal to expose the lateral surface of the anterior paroccipital process (indicated by arrow). The quadrate notch is lateral to the anterior paroccipital process and would have housed the quadrate. Petrosal is colored red. Squamosal is colored yellow.

**Fig 23:**
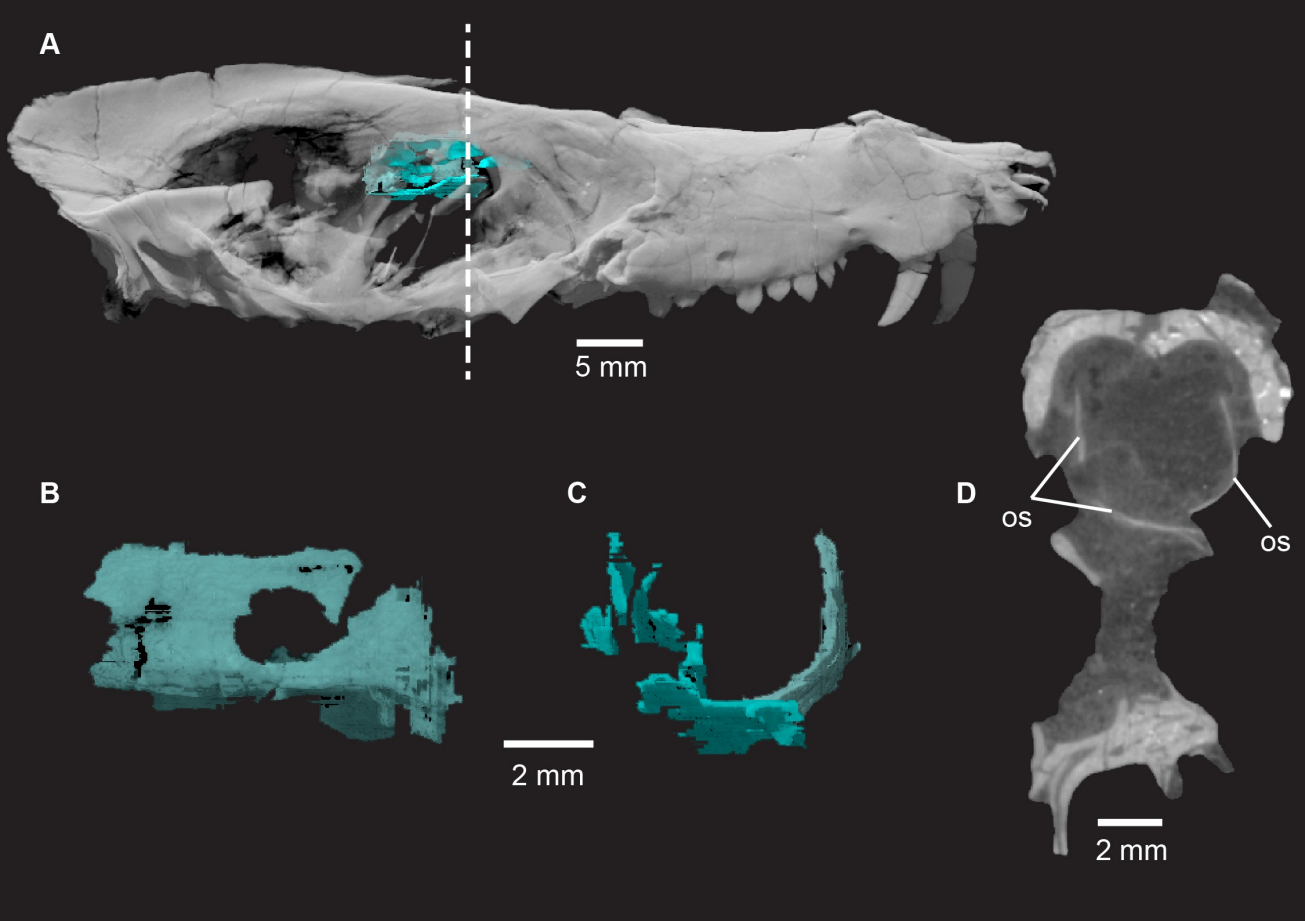
***Pseudotherium argentinus*, orbitosphenoid (os) in situ (A), left lateral view (B), anterior view (C), and cross section (D).** Left and right orbitosphenoids contact ventrally at midline. Left orbitosphenoid is less fractured than right orbitosphoid and shows distinct optic foramen. Cranium rendered semitransparent to illustrate relationship of orbitosphenoid to sphenorbital fissure and surrounding bony elements. Cross section illustrates the relatively dorsal position of the brain within the cranium.

**Fig 24:**
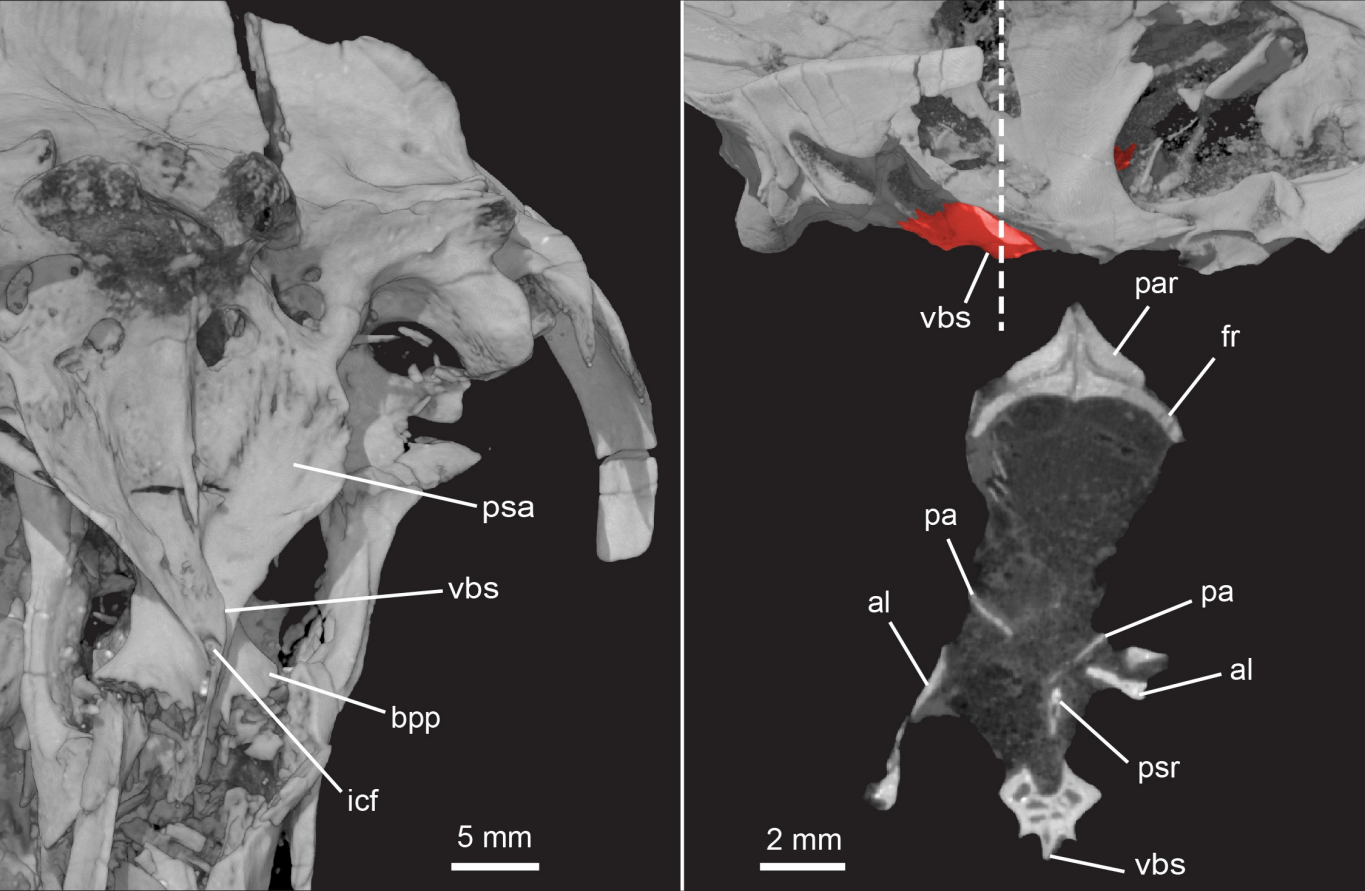
*Pseudotherium argentinus*, ventromedial crest of basisphenoid. **(Left) Ventromedial crest of basisphenoid in left ventrolateral view.** (Top right) Parabasisphenoid complex in left lateral view, colored red-orange, and (bottom right) ventral crest of basisphenoid (vbs) in cross section. Dashed line indicates position of cross section. Abbreviations: al, alisphenoid; bpp, basipterygoid process; fr, frontal; icf, internal carotid foramen; os, orbitosphenoid; pa, pila antotica; par, parietal; psa, parasphenoid ala; psr, parasphenoid rostrum; vbs, ventral process of basisphenoid.

**Fig 25:**
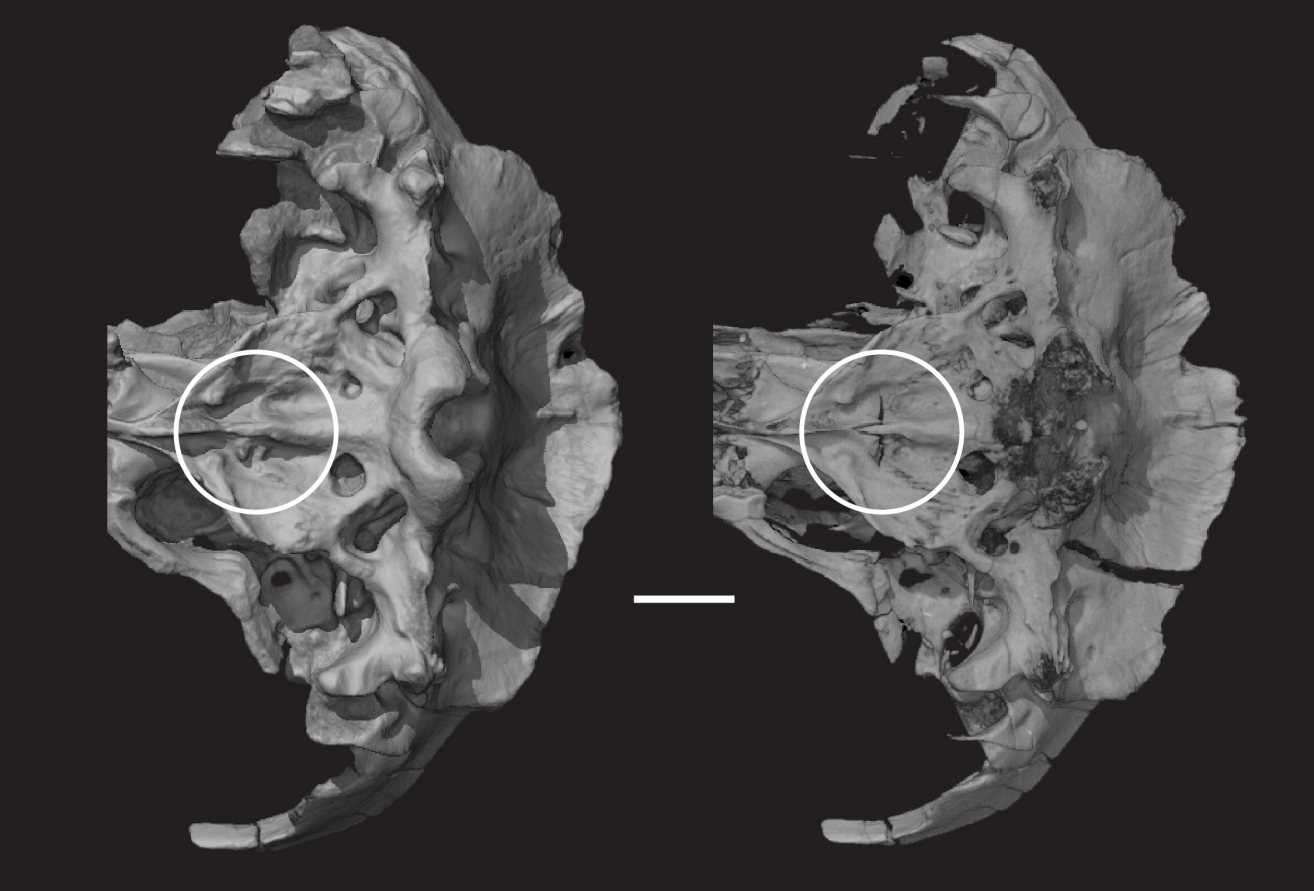
*Pseudotherium argentinus*, suture between basioccipital and basisphenoid. Suture between basioccipital and basisphenoid is indicated by the circle. The suture is distinct, marked by a wide gap and flaring articulating surfaces on both elements. An anterior process of the basioccipital overlaps the basisphenoid posteroventrally and medially. Note how different the suture looks in an isosurface model with matrix included in the rendering (left) and a digitally prepared volume render (right).

**Fig 26:**
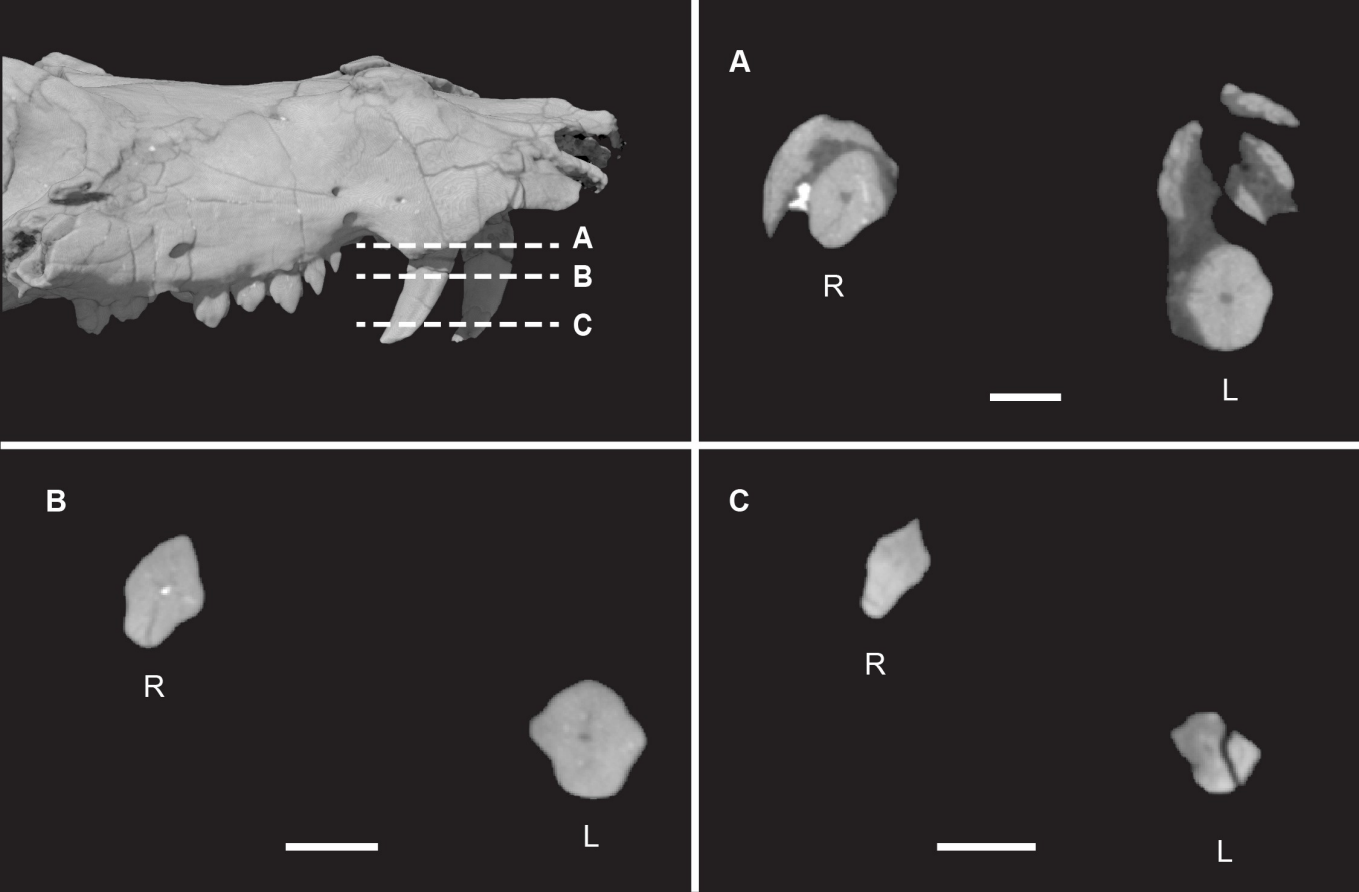
*Pseudotherium argentinus*, cross sections through canines. (A) right side of snout showing location of cross sections (B), (C), and (D). R = right, L = left. Arrows indicate labial and lingual ridges, an autapomorphy of *Pseudotherium*.

**Fig 27:**
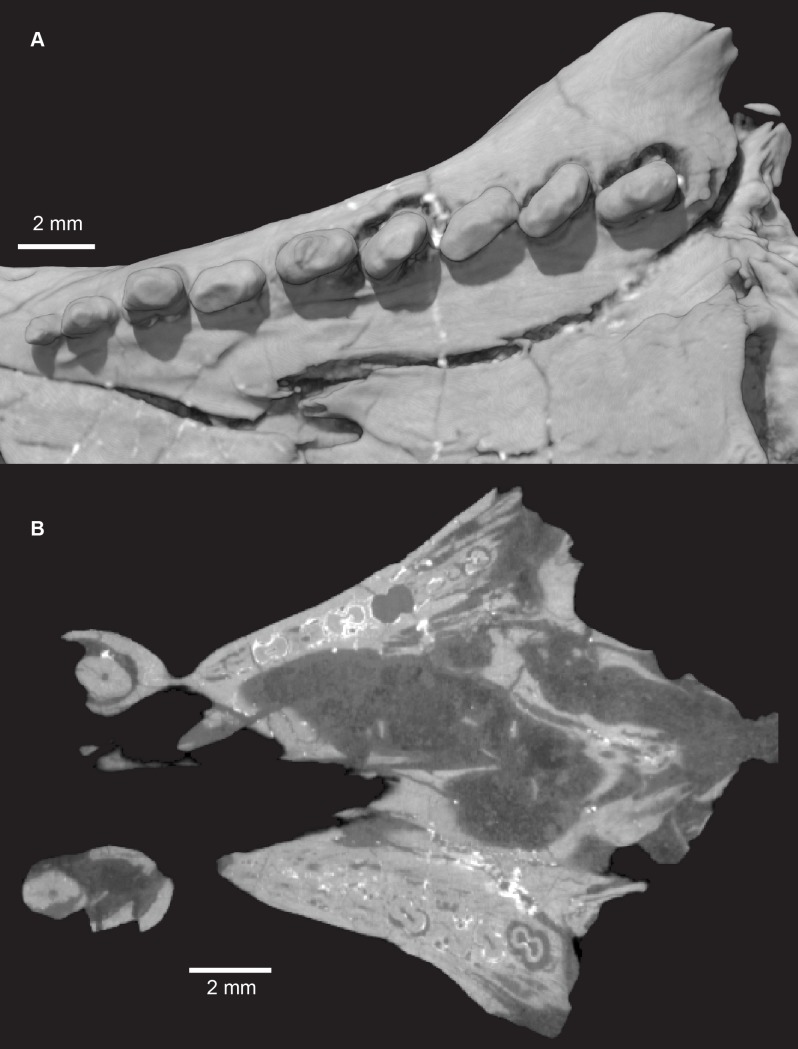
*Pseudotherium argentinus*, left postcanine tooth row. (A) Occlusal view of left row in volume render. There are nine postcanines which increase in complexity from anterior to posterior, with the first postcanine being a single cusp and the penultimate postcanine having the most distinct cusps. Despite this trend, the postcanine cusps are small and blunt relative to postcanine cusps seen in other cynodonts. More posterior crowns (PC6-9) are mesiolingually in-turned. (B) Left maxillary tooth row in disto-occlusal view. Small accessory cusps are visible distobuccally on PC7 and PC8. (C) Horizontal section through the snout of PVSJ 882 illustrating constricted roots with a figure-eight cross section. The pulp cavity is compressed but never completely divided between root lobes. Nutrient canals run through each lobe of the root.

**Fig 28:**
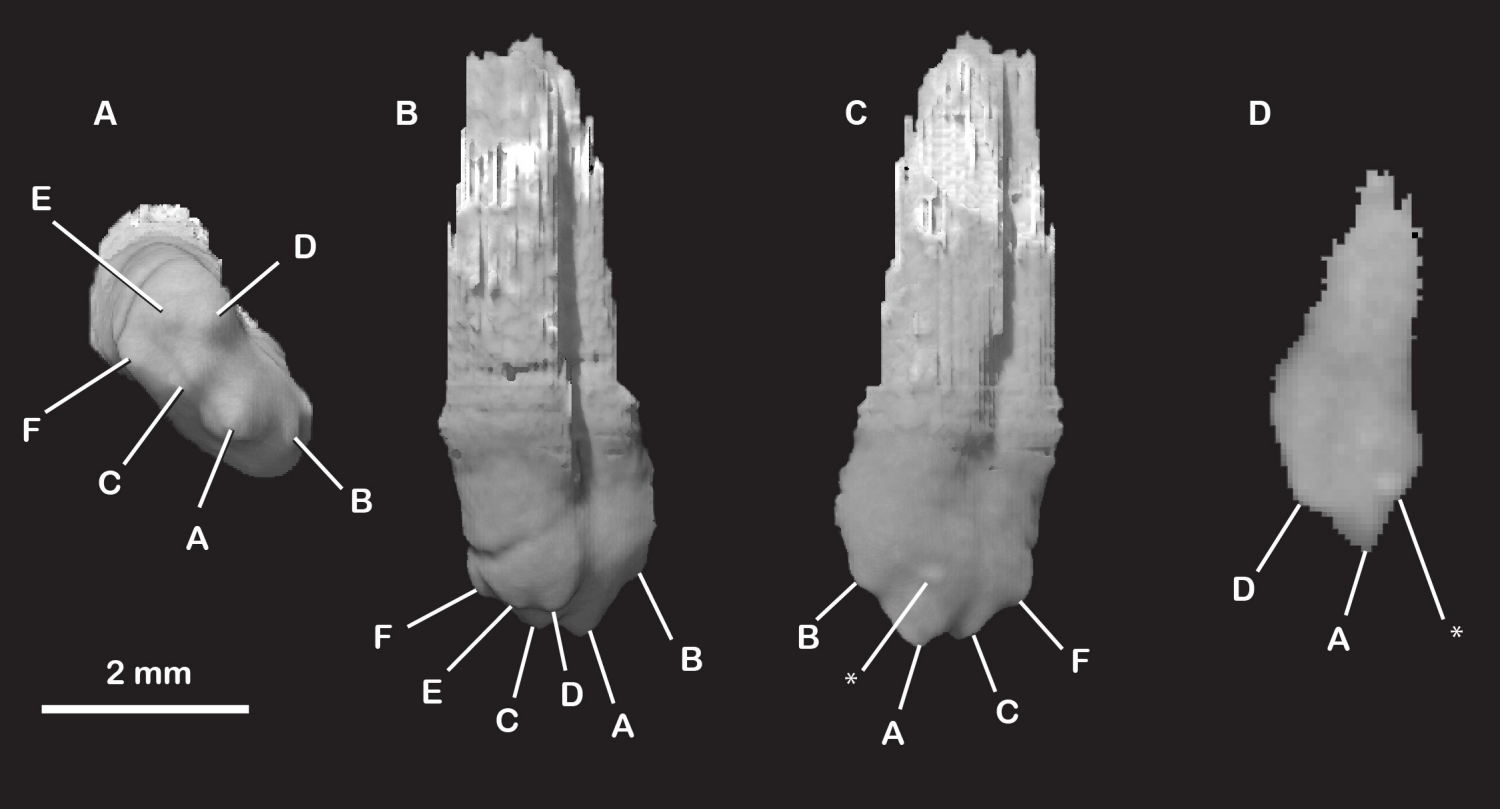
*Pseudotherium argentinus*, isolated right postcanine, PC8. (A) Occlusal, (B) buccal, (C) lingual, and (D) cross sectional views. The eighth postcanine on the right side of the skull best illustrates the cusp pattern of the distal postcanines. Three main cusps are in alignment with the longitudinal axis of the crown (A, B, C). Three accessory cusps surround the most-distal main cusp buccally (D), distally (E), and lingually (F). A small bump appears on the surface of the crown in the volume rendering (*). Cross sections confirm that it is not a cusp, but an artifact from denser material.

**Fig 29:**
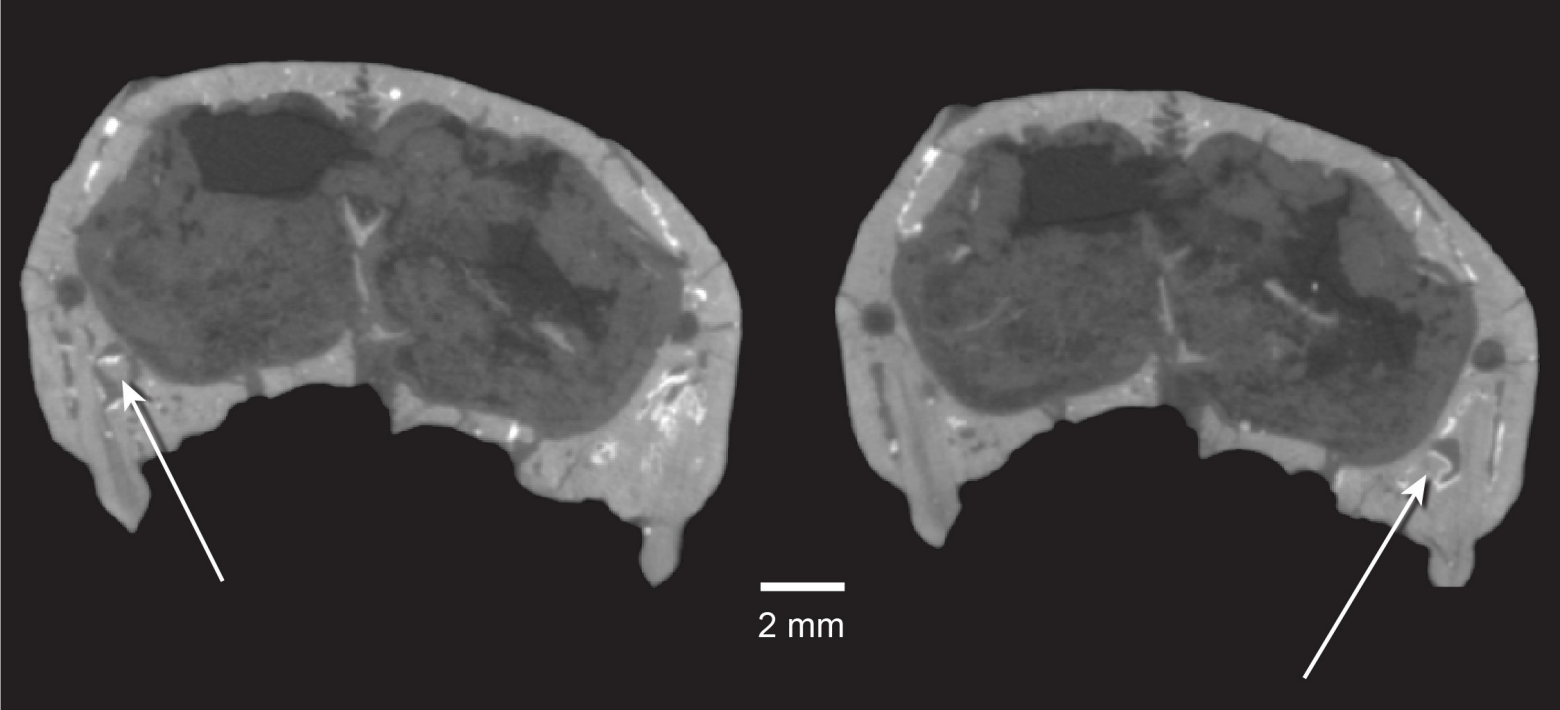
*Pseudotherium argentinus*, tooth replacement. Cross sections through left (left) and right (right) upper fourth postcanines (PC4) illustrating possible replacement teeth, indicated by arrows. Apparent replacement crowns are positioned near the distal margin and apex of the P4 roots.

**Fig 30:**
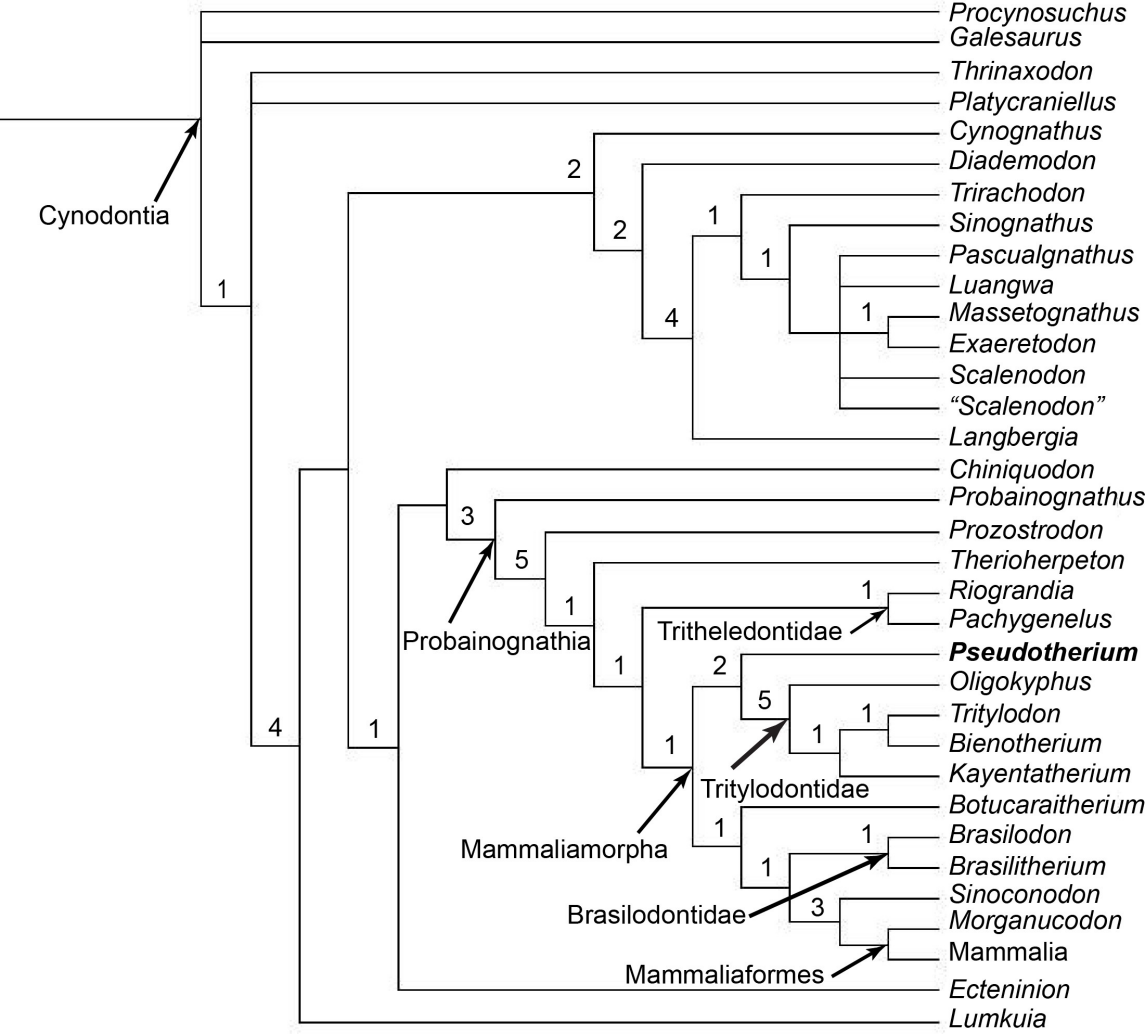
Strict consensus tree. Strict consensus tree of all eight most parsimonious trees (tree length = 443, CI = 0.4695, RI = 0.7814) obtained using PAUP*4.0b10. Characters are unordered. The number to the left of the node is the decay index of that clade.

#### Type locality

Valle Pintado in Ischigualasto Provincial Park, San Juan Province, Argentina (S 30° 08' 14", W 67° 52' 39"). The single known specimen was discovered 40 m above the base of the Ischigualasto Formation, in the upper portion of the La Peña Member (sensu [58]) and lower portion of the *Scaphonyx-Exaeretodon-Herrerasaurus* biozone. The holotype (PVSJ 882) was found intermixed with the holotype (PVSJ 874) of the basal sauropodomorph *Panphagia protos* [68] and an unnamed lagerpetid dinosauromorph (PVSJ 883) [57].

#### Age

Late Carnian on the basis of a radioisotopic date near the base of the Ischigualasto Formation in the vicinity of the type locality [55]. This date was recently recalibrated to 231.4 ± 0.3 Ma [56].

**Diagnosis**: *Pseudotherium argentinus* is a probainognathian cynodont possessing the following combination of features: the lacrimal contributes extensively to the floor of the orbit; the frontal has a long orbital process that contacts a short orbital process of the palatine near the floor of the orbit; the orbital process of the palatine is low and contributes little to the orbital wall; the prefrontal is superficially large and extends anteriorly, medial to the lacrimal, contributing to the lateral wall of the nasopharyngeal passage; a vestige of the postorbital bone is preserved behind the orbit, but lacks an ossified postorbital bar; the interpterygoid vacuities remained open throughout life; laterally flaring parasphenoid alae intersect at an obtuse angle between their contacts with the petrosal promontorium; there is a longitudinal ventral process on the basisphenoid; the lambdoidal crest strongly overlaps the occipital plate; CT scans show large, open spaces within the spongy bone of the parietal, petrosal, squamosal, basioccipital, basisphenoid, supraoccipital, and exoccipital surrounding the braincase; the vertical margin of the petrosal (prootic) lateral flange is notched; the upper canines are long, laterally compressed and non-serrated with a ridge on both their labial and lingual surfaces; there are nine upper postcanine teeth with the first postcanine consisting of a single cusp, while blunt, indistinct cusps form the crowns of the remaining postcanines. *Pseudotherium argentinus* is larger than most of the other ‘taxa of interest’ mentioned above, measuring 69 mm minimum length, not including the missing premaxillae.

**Maturity at time of death:** A number of features suggest that the holotype was approaching full skeletal maturity at time of death. The sagittal and lamdoidal crests are well developed; the orbit is relatively small compared to other skull proportions; the prootic and opisthotic are fused to form the petrosal; and extensive fusion has occurred between the basioccipital and exoccipitals, and between the tabular, supraoccipital, and interparietal. Additionally, a short diastema between the canine and the first postcanine suggests that a tooth had been shed and not replaced. There are also irregular wear facets on all of the postcanine tooth crowns. The only suggestions of immaturity include the presence of a pair of small un-erupted replacement postcanine crowns situated at the base of the right and left fouth postcanine roots, visible in the CT scans, and possibly the presence of an interpterygoid vacuity.

### Description of the skull

The specimen is relatively three-dimensional, although there is evidence that the facial portion is slightly dorsoventrally crushed. The skull is long and narrow, with distinct sagittal and lambdoidal crests that overhang the slanting occiput. The left side of the skull is more distorted than the right, with the apical end of the canine crown leaning medially and some of the orbital and braincase elements displaced or missing. The snout is constricted both dorsoventrally and mediolaterally behind the long canines. It has nine postcanines that are relatively small with simple blunt, rounded cusps, and the secondary palate extends slightly posterior to the maxillary tooth row.

#### Premaxilla

The premaxillae are almost entirely missing in the holotype of *Pseudotherium*. All that remains are short fragments of the posterior extremities of the right and left medial palatine (palatal) processes, which are visible in CT sections between the incisive fossae (paracanine fossae) for the lower canines. As in *Brasilitherium* [54] the medial margin of each has a dorsally directed process suggesting that the premaxillae abutted but did not fuse on the midline. As preserved, they are slightly separated in both *Pseudotherium* and *Brasilitherium*, but in life the premaxillae probably met on the midline behind the sphenopalatine fissure. Compared to *Brasilitherium* the medial palatine process in *Pseudotherium* is thin and delicately built. Underlying the premaxillary palatine process is the medial palatine (palatal) process of the maxilla (Fig 5). The snout is too incomplete to determine whether the posterior border of the sphenopalatine fissure was formed by the premaxillae, maxillae, or both.

#### Septomaxilla

The facial process of the septomaxilla forms the ventrolateral border of the external naris, and the transverse shelf or footplate forms its floor. The facial process extends from the external naris posteriorly to wedge between the nasal and the maxilla, tapering to a point immediately anterior to the canine root. A septomaxillary canal traverses the length of the footplate (Fig 6). The posterior opening of the canal is roughly level with the anterior end of the vomer in coronal section. The canal opens anterolaterally and, posteriorly, its lateral enclosure is completed by the facial process of the maxilla. The canal probably conveyed the nasolacrimal duct forward to its anterior terminus on the floor of the narial cupola, near the presumed position of the aperture of the vomeronasal organ [52,54]. These anatomical relations represent the ancestral condition for probainognathian cynodonts.

#### Maxilla

The maxilla comprises much of the lateral surface of the snout, forming the lateral wall of the nasopharyngeal passage and the anterior half of the secondary palate. It contacts the septomaxilla and premaxilla anteriorly, the nasal dorsomedially, and the palatine, prefrontal, and lacrimal posteriorly. It supports the vomer for a short distance, but internal damage to the snout complicates interpretation of the relations of these two bones. The facial process of the maxilla extends anterior to the canine, where it contributes to a precanine diastema, to the level of the orbit posteriorly. It reaches its greatest height above the upper canine root, where the nasals only narrowly separate the right and left maxillae on the dorsal midline. Dorsoventral postmortem compression of the rostrum has caused the canine roots to rupture and weather through the maxilla.

The maxillary palatal process extends anterior to the upper canine and encircles much of the incisive (paracanine) fossa, which accommodated the lower canine tip when the jaws were closed (Figs 3–5). The secondary palate is long and ends 3–4 mm posterior to the distal-most tooth. The maxilla forms the anterior half of the secondary palate, its contribution ending posteriorly at the level of the fifth postcanine tooth where it contacts the palatal process of the palatine. Behind this point, the maxilla forms the root of the zygomatic arch and contacts the lacrimal along the anterior rim of the orbit. The maxilla also extends beneath the orbit as a bony shelf. In a highly unusual (apomorphic) relationship, however, the lacrimal expands over the orbital floor, covering the maxilla and largely excluding its participation in the orbital floor or in the border of the subtemporal fenestra (Fig 7).

The maxillary canal courses the entire length of the facial process and, in life, transmitted the maxillary branch of the trigeminal nerve, innervating the upper dentition, and conveying cutaneous branches from the surface of the snout (Fig 8). The canal opens through the anterior floor of the orbit, and then runs above the postcanine tooth roots. It exits externally via three foramina on either side of the face that are bilaterally symmetrical in size and position (Fig 8). The smallest and most anterior foramen is positioned behind the canine root. Two other foramina are aligned dorsoventrally and are positioned over the canine-postcanine diastema. The foramina positions and the branching pattern of their associated canals are similar to the pattern reconstructed for *Ecteninion* [69]. If that reconstruction is accurate, the dorsal foramen and the small anterior foramen in *Pseudotherium* contained the internal nasal nerve, while the ventral foramen contained the superior labial nerve. The largest foramen on the lateral facial process is positioned anterior to the root of the zygomatic arch above the roots of postcanine teeth 5 and 6. It is the exit to a canal that has an independent origin from the maxillary canal. According to Benoit et al. [69], it contained the caudal alveolar ramus of the maxillary nerve. This arrangement of maxillary foramina is similar to *Brasilodon* [17] and *Brasilitherium* [54], *Irajatherium* [37], and at least some tritylodontids [70]. It reflects a reduction in number from the multiple cutaneous foramina present in cynodonts plesiomorphically [47,71], and a stabilization of their numbers and position on the face.

#### Jugal

Little of the jugal is preserved in the holotype of *Pseudotherium*, save for a sliver of bone wedged between the maxilla and the lacrimal at the root of the zygomatic arch (Figs 3 and 4). The root of the zygomatic arch is largely formed by the maxilla. However, the jugal probably contributed to its dorsal portion as suggested by a deep groove between the maxilla and the lacrimal just posteromedial to the jugal fragment.

#### Nasal

The nasal forms the dorsal margin of the naris and the roof of the nasopharyngeal passageway. It contacts the facial process of the septomaxilla, and it passes along the length of the maxillary facial process where the two bones share a beveled scarf contact that lacks complex interdigitation. The nasal has a short contact with the prefrontal, and meets the lacrimal just anterior to the orbital rim. The nasal is constricted on the snout by the maxilla between the roots of the canine, and it then expands posteriorly where it achieves its greatest width at the front of the orbit where the maxilla, perfrontal, and lacrimal are in contact (Figs 3 and 4). The internasal sutural boundary has shallow interdigitations along their sutural boundary. The nasal tapers to a thin plate at its rear extremity, where it overlaps the frontal. A nutrient canal runs through the nasal roof at its posterior end.

#### Vomer and possible ethmoid ossifications

CT scans of *Pseudotherium* show thin, delicate elements in the nasal cavity that may reflect the early evolutionary onset of ossification of the nasal capsule (Fig 9). The fragile elements are comparable to those illustrated for *Brasilitherium* [54], but the interpretation of these elements in both *Pseudotherium* and *Brasilitherium* is complicated by fragmentation of other bony elements into the lumen of the nasopharyngeal passageway, a lack of bilateral symmetry, and mottling of the matrix that fills the passageway. In *Brasilitherium*, these elements were interpreted to be ossified nasal turbinates [54], though comparison with the tritheledontid *Elliotherium* suggests present evidence is insufficient [72]. In *Pseudotherium*, the vomer is broken and detached from the maxilla, and these fragmentary bony structures may simply be exfoliated from the inner surface of the maxilla. Extensive ossification of the nasal capsule is apomorphic of crown Mammalia [1,52], and no unequivocal intermediate ossifications have been reported in any other fossils. Because the phylogenetic positions of *Pseudotherium* and *Brasilitherium* are not far outside of crown Mammalia, these peculiar structures warrant further discussion (see Discussion section below).

The vomer in *Pseudotherium* has drifted from its rostral articulation atop the palatal processes of the maxillae and palatines. The posterior end of the vomer is visible in ventral view where it forms a triangular plate in the roof of the choana. It tapers posteriorly to a median point, bordered by the pterygoid. The vomer forms the anterior end of a ventral midline ridge on the primary palate that begins at the choana and extends onto the pterygoids, and it may have been continuous with a keel on the basisphenoid that extends onto the basioccipital. The midline keel may have been the site of attachment for the median raphe of the pharyngeal constrictors [70] and the posterior pterygoid muscle [73]. The vomer apparently diminishes in height anteriorly, and it becomes C-shaped on either side where it wrapped around the vomeronasal organ (Fig 5).

#### Lacrimal

The lacrimal contributes broadly to the orbital wall and floor and forms the anterior orbital rim (Figs 3 and 7). Its long facial process is exposed for a short distance on the lateral side of the snout in front of the orbit, but its rostral extremity continues anteriorly, concealed laterally beneath the facial process of the maxilla. In this region, the lacrimal forms part of the wall of the nasopharyngeal cavity. The lacrimal is pierced by two lacrimal foramina, probably for the ducts of the lacrimal and Harderian glands [52], that open along the anterior rim of the orbit. Passing forward they become confluent and merge into a single canal that is enclosed for a short distance by the lacrimal facial process, before emptying into the nasopharyngeal cavity.

The structure of the lacrimal in the orbital wall and floor is quite unusual. The lacrimal contacts the nasal and prefrontal anteromedially above the orbit. The orbital plate of the lacrimal is a thick bone that overlies and conceals a broad ventral sheet-like expansion of the prefrontal. The orbital process of the lacrimal forms almost the entirety of the anteromedial wall of the orbit, meeting a very short process of the palatine near the floor of the orbit, and a long process of the frontal that completes the posterior margin of the orbital wall. The lacrimal forms most of the floor of the orbit, covering most of the maxilla and largely excluding it from participation in the orbit.

#### Palatine

The palatine forms the posterior half of the secondary palate, the rear walls of the nasopharyngeal passage, the anterolateral end of the primary palate, and makes a minor contribution to the ventral wall of the orbit (Figs 3 and 7). Its palatal process contacts the maxilla anteriorly, while its dorsal plate contacts the vomer medially, and the pterygoid posteriorly. The lateral margin of the dorsal plate extends far posteriorly, forming a ridge that outlines the lateral margin of the primary palate. This lateral ridge was illustrated in *Brasilitherium* [21] and is similar to the palatal ridges in *Morganucodon* [74] and tritylodontids [70].

The orbital process of the palatine (also referred to as the dorsal, or ascending, process of the palatine) is short, rising just above the lateral flange of the pterygoid when the specimen is viewed laterally. The short orbital process is wedged between the lacrimal anteriorly and the frontal posteriorly, and it forms the ventral-most portion of the orbital wall. The orbital process of *Pseudotherium* is shorter than in other probainognathian cynodonts. The orbital process of other taxa of interest is taller (dorsoventrally) than it is long (anteroposteriorly), and it forms the posteroventral border of the orbital wall in *Ecteninion* [45], *Prozostrodon* [75], *Riograndia* [32], *Brasilitherium* [22], and *Morganucodon* [74].

The orbital process of the palatine in *Kayentatherium* is illustrated as a low structure, as in *Pseudotherium*. However, unlike *Pseudotherium*, the orbital process of *Kyentatherium* is also long, extending to the alisphenoid (eptipterygoid, [70]). In all of the taxa mentioned above, the orbital process of the palatine contacts the frontal dorsally, the lacrimal anteriorly, and the orbitosphenoid posteriorly. However, the orbital region is reportedly damaged in many specimens as a result of mechanical preparation, and therefore difficult to interpret with certainty. Owing to the high, medial position of the orbitosphenoid in *Pseudotherium*, it can be stated with confidence that the palatine did not contact the orbitosphenoid. The orbital process of the palatine in *Pseudotherium* is shorter dorsoventrally than in any other non-mammalian cynodont.

Two or three foramina are associated with each palatine; the greater palatine foramen at its anterior border with the palatal process of the maxilla, and one to two foramina for the lesser palatine nerve on the lateral margin of the palatine where the palatal process and the dorsal plate connect.

#### Pterygoid

Although broken and with displaced parts, the pterygoid is fairly complete preserving the three processes found in other non-mammalian cynodonts: the anterior (palatal) process, the transverse process (lateral flange), and the quadrate process. Its pattern of troughs and ridges (Figs 10 and 11) resembles the pterygoid of other probainognathians including *Morganucodon* [74], *Kayentatherium* [70], *Sinoconodon* [76,77], *Brasilitherium* [22], and *Riograndia* [32]. This pattern may also be present in *Pachygenelus* [22] but as yet it is unknown in *Brasilodon*.

The horizontally oriented anterior process of the pterygoid forms the rear end of the primary palate. The lateral edges of the primary palate are defined by a ridge formed by the palatine and pterygoid. This lateral ridge deepens posteriorly and it likely formed the ventromedial process of the pterygoid as in *Brasilitherium* [21]. Barghusen [73] called these ‘choanal crests’ and maintained that they indicate the presence of a soft palate that was a direct continuation of the osseous secondary palate. He summarized older literature [78–80] in arguing that the ridges themselves suggest the attachment of soft tissue, that a deep channel for the air passage is positioned dorsal to the crests, and that the choanal crests are continuous with the bony secondary palate.

Where the left and right pterygoids meet in the roof of the choana is a well-developed median keel that passes forward onto the vomer and ends near the front of the choana. This keel is unlike anything known in extant mammals. The keel does not extend into the nasopharyngeal passage as a bony structure, as in *Kayentatherium* [70] and *Morganucodon* [74], but it may have continued anteriorly as a cartilaginous nasal septum dividing the nasopharyngeal passage [72].

At the posterior end of the lateral ridge of the pterygoid is the root of the transverse process (lateral flange). The root of the transverse process projects anteriorly and contacts the posterolateral-most corner of the palatine. In lateral view, the lateral flange deepens posteriorly and is deepest is where the so-called ‘pterygoid wing’ [21] would have projected ventrally had it not broken off postmortem. The pterygoid wing borders a posteriorly facing concavity on the transverse process that may have served as the origin for the posterior division of the pterygoid musculature [22]. Similar concavities are described in *Brasilitherium* [22], the tritylodontid *Kayentatherium* [70], and the mammaliaform *Megazostrodon* [81]. The severely reduced pterygoid transverse processes in crown mammals complicates their interpretation with respect to muscle attachments in non-mammalian cynodonts.

Where the transverse process meets the lateral ridge, the pterygoid continues posteriorly where it outlines open interpterygoid vacuities. The interpterygoid vacuities are generally thought to be open in early ontogeny of basal cynodonts, and to close at maturity [35]. Closure of the interpterygoid vacuities is associated with maturity in *Thrinaxodon*, galesaurids, *Diademodon*, basal probainognathians, and basal mammaliaforms, and they remain open in juveniles of some of those taxa [35]. Interpterygoid vacuities have been described among juvenile tritylodontids as well as “randomly in some [seemingly] adult specimens” [35]. Open interpterygoid vacuities are described in arguably mature specimens of *Brasilodon*, *Brasilitherium*, and in tritheledontids where the mesocranial region is preserved [17,35]. Considering the many features suggesting that the holotype of *Pseudotherium argentinus* was approaching full skeletal maturation at time of death (above), it apparently shares with these taxa the condition of interpterygoid vacuities that remain open throughout life.

The pterygoid is overlapped by the alisphenoid dorsolaterally (Fig 11). The pterygoid ends medially where it sutures to the robust basipterygoid process of the basisphenoid. The quadrate ramus (process) of the pterygoid projects posterolaterally to where it is appressed to the ventromedial surface of the quadrate ramus of the alisphenoid. It ends where the quadrate ramus of the alisphenoid contacts the lateral flange of the petrosal.

#### Prefrontal

The prefrontal is reportedly absent in all of the various specimens referred to as brasilodontids and tritheledontids (e.g., [17,18,25,28,72]) and in *Therioherpeton* [75]. However in *Prozostrodon brasiliensis*, although the postorbital arch is absent, remnants of the prefrontal and postorbital bones persist in their plesiomorphic position in the orbital margin (Figs 3 and 12). The contact between prefrontal and postorbital that is plesiomorphic for Cynodontia [47] is interrupted in *Prozostrodon* by a lateral process of the frontal, which also participates in the orbital rim [75].

*Pseudotherium* preserves a condition similar to *Prozostrodon* in which the postorbital arch was not simply broken away but was probably absent as an ossified bar in life, and the prefrontal and postorbital persist in the orbital margin, along with a short process of the frontal. Externally the prefrontal is positioned in its primitive position on the anterodorsal corner of the orbit, lying between the lacrimal anteriorly, the frontal posteriorly, the nasal anteromedially, and the parietal posteriorly. CT data reveal that the prefrontal is far more extensive than can be observed on the surface of the skull and that it forms an unusually broad flat plate that extends into the inner surface of the snout, internal to the lacrimal. Its anterior extremity is almost at the level of the lacrimal.

The prefrontal was also tentatively identified in *Brasilitherium*, occupying the same position on the dorsolateral edge of the orbital rim as in *Pseudotherium* [54]. Its external sutural relationships are difficult to observe in the published 3D surface rendering of *Brasilitherium*; a volume rendering would likely show the sutures more clearly. The prefrontal is unequivocally absent in tritylodonts and other members of Mammaliamorpha [1,15,62]. Although we follow published accounts in our scored matrix, it is clear that CT scans are needed to confirm its absence in titheledonts and *Therioherpeton*.

#### Postorbital

Both zygoma are broken in *Pseudotherium*, but the skull roof is sufficiently well-preserved to show the persistence of a vestigial postorbital bone. The preserved zygoma of *Brasilitherium* indicates that the postorbital arch was not ossified [54], as is likely the case in *Pseudotherium*. The postorbital bone is seen as a small flat plate appressed against the lateral surface of the frontal and posterior to the orbit. Cross sections show a sliver of frontal wedged between the putative postorbital and the parietal, confirming that the element is distinct (Fig 12). The juxtaposition of the reduced postorbital and large prefrontal bones suggests that evolutionary modification of the orbital boundary occurred in at least two steps. First came the loss of an ossified postorbital arch, and only later did the postorbital and prefrontal bones to fail to ossify entirely, in a condition diagnostic of Mammaliamorpha, or perhaps Mammaliamorpha + Tritheledontidae [1,15].

#### Frontal

The frontal forms the dorsal margin of the orbit. A hooked, fingerlike orbital process projects ventrally and forms the posterior margin of the orbital wall. Posterior to it is a large orbital fissure. The orbital process of the frontal contacts the orbital process of the palatine deep in the ventromedial part of the orbit. In lateral view, the frontal is overlapped anteriorly in the orbit by the lacrimal, and anterodorsally by the prefrontal. Posteriorly, a small portion of the alisphenoid overlaps the frontal. In dorsal view the frontal is overlapped anteriorly by the nasals, and posteriorly by the parietals. A supraorbital foramen is positioned between the frontal, tapering nasal processes, and the prefrontal. This foramen has also been identified in *Brasilitherium* [22] and in *Riograndia* [32,82] and may have transmitted a cutaneous branch of the opthalmic nerve.

A study of *Brasilitherium* based on μCT [60] suggested that the frontal enclosed an enlarged olfactory bulb compared to more basal cynodonts, and on this basis speculated that *Brasilitherium* represented an increase in olfactory performance compared to non-mammaliaform cynodonts. However, coronal CT scans through the frontal of *Pseudotherium* show a comparable curvature of the frontal over the top of the olfactory bulb, and its superior preservation shows that the plesiomorphic orbitosphenoid also occupied part of this space beneath the frontal (Fig 13). The geometry of the space enclosed between the alisphenoids in *Brasilitherium* and *Pseudotherium* reveals a very minor apparent increase in relative size of the olfactory (piriform) cortex compared to more basal cynodonts. Ontogenetic interdependencies observed in the development of living mammals indicate that increases in olfactory bulb volume induce enlargement of the olfactory cortex [5,7]. This correlated expansion suggests only minor improvement in olfactory capabilities in these taxa. There is no clear evidence that the mature telencephalon had yet differentiated into neocortex and olfactory cortex in either *Brasilitherium* or *Pseudotherium*, emphasizing their general plesiomorphic organization compared to members of Mammaliaformes [2,5,7].

#### Parietal

The parietals are largely confined to the intertemporal region, forming the roof over a narrow endocranial cavity, and presenting an extremely long attachment area for the temporalis musculature. There is no pineal foramen, nor is there any hint of an impression on the undersurface of the parietals indicating the persistence of a pineal eye beneath the skull roof. The parietal’s anterior extent is notable, overlapping the frontal dorsolaterally and terminating as a thin process that almost reaches the nasal. The anterior process of the parietal overlaps the rear part of the prefrontal. The left and right parietals meet on the midline and fuse posteriorly above the alisphenoid and prootic, where they form a tall sagittal crest that protrudes posteriorly beyond the level of the occipital condyles. The sagittal crest is posterolaterally continuous with the lambdoidal crest and the supraoccipital. Where the base of the fused parietals forms the roof of the endocranial cavity, CT scans indicate that they enclose an extensive network of hollow cavities, larger than what is typically seen of diploё (Fig 14). Much like the condition in *Brasilitherium* [21] and mammaliamorphs [1,15] the lateral flange of the prootic is tall and has extensive lateral overlap onto the lateral face of the parietal. The posterior edge of the alisphenoid also overlaps the lateral surface of the parietal.

#### Squamosal

The squamosal extends from its medial contact with the parietal at the base of the sagittal crest laterally over the supraoccipital and petrosal to form the face of the prominent lambdoidal crest. The temporal processes are incomplete in *Pseudotherium*, more so for the left than the right side. A shallow V-shaped notch separates the temporal process of the squamosal from the lambdoidal crest. Below its parietal contact, the squamosal contacts the anterior lamina of the petrosal in the medial wall of the temporal fenestra. Descending from the root of the temporal process is a deep flange (Fig 15). Cutting into the flange are two deep notches, separated by a hook-shaped squamosal septum. The lateral notch held the quadratojugal, while the dorsal plate of the quadrate was wedged into the medial notch [83], however neither the quadrate nor quadratojugal is preserved in articulation. Medial to the quadrate, the squamosal abuts against the paroccipital process. It does not completely cover the paroccipital process and it is likely that this failure of complete coverage permitted the paroccipital process to contact the quadrate. This organization of the squamosal flange is similar to *Brasilitherium* [22] and many basal mammaliamorphs [1,15].

#### Quadratojugal

The quadratojugal is the smallest bone in the skull. The left quadratojugal is preserved embedded in the base of the squamosal flange, where it occupies the quadratojugal notch (Fig 16). This position of a tiny splint-like quadratojugal bone embedded in its own notch in the ventrum of the descending flange of the squamosal is the plesiomorphic condition as reported in *Kayentatherium* and other cynodonts [70,83].

The presence of a quadratojugal in its own notch distinct from the quadrate notch is apomorphic to eutheriodonts (Therocephalia + Cynodontia) [1,81]. The quadratojugal is unknown in tritheledonts owing to non-preservation. Although unknown in *Morganucodon*, its presence is indicated by a clearly defined quadratojugal facet in its primitive position on the front of the lateral flange of the quadrate [74,81].

Luo [84] argued that the quadratojugal was lost in tritheledontids and Mammaliaformes, and that it re-evolved in *Brasilitherium*, based in part on the phylogenetic hypothesis that tritylodontids represent a basal radiation of herbivorous cynodonts [84]. Luo also reconstructed the middle ear of *Brasilitherium* with the quadrate and quadratojugal wedged together into a single notch [8,53,84]. In *Pseudotherium*, two depressions adjacent to the paroccipital process can be seen within the quadrate notch, but there is no doubt that it retains the plesiomorphic condition of a quadratojugal having its own notch separate from the quadrate. The craniomandibular joint and middle ear regions in *Brasilitherium* may be more similar to *Pseudotherium* than suggested by prior reconstructions of *Brasilitherium*. In any event, the phylogeny recovered in this analysis (below) supports the interpretation that little variation affected the shape and attachment of the quadratojugal in cynodont evolution, until the quadratojugal was ultimately lost in the last common ancestor of Mammalia [1,15,81].

#### Quadrate

The quadrate is not preserved in *Pseudotherium*, but the notch for the insertion of its dorsal plate is incised into the ventral edge of the squamosal. The quadrate of *Brasilitherium* has been described as being very similar to *Morganucodon* in structure [22], having a stapedial process that overlaps the anterior process of the paroccipital process [22,83]. Because the squamosal flange closely resembles *Brasilitherium*, the quadrate of *Pseudotherium* may have been similar to *Brasilitherium*.

#### Stapes

An incompletely preserved right stapes (Fig 17) was broadly perforated, with separate anterior and posterior crua. Part of its footplate is wedged deeply into the fenestra ovalis, and its anterior crus projects about half the distance between the fenestra ovalis and the paroccipital process. Missing are the distal end of the anterior crus, most of the posterior crus, and the ‘head’ of the stapes that articulated with the quadrate. The footplate is “C”-shaped as preserved. It is likely that half of the footplate is missing and the “C” shape is the result of breakage along a central concavity such as described for *Morganucodon* [74] and *Brasilitherium* [53].

#### Petrosal

The prootic and opisthotic are fused to form the petrosal (periotic). A line resembling a suture between the crista interfenestralis and the paroccipital process is the clearest superficial indication that the prootic and opisthotic were at one time distinct. However, CT cross sections of that region reveal no trace of a suture and show that the prootic and opisthotic are coossified. For descriptive purposes, the prootic portion of the petrosal will be referred to simply as the prootic.

The prootic encloses the otic capsule and forms the posterolateral portion of the braincase. The membranously-derived anterior lamina projects forward to contact the rear edge of the alisphenoid. The anterior lamina is separated from the alisphenoid by two foramina for the maxillary (V_2_) and mandibular (V_3_) branches of the trigeminal nerve. The prootic flares laterally, forming a broad anterior lamina that slopes almost vertically at its lateral-most edge (Fig 18). It sits atop and abuts the quadrate ramus of the alisphenoid. The lateral flange is perforated by a vascular foramen, probably for the vena cava lateralis [85], located posterior to the V_3_ foramen. Posterior to the V_3_ foramen is a small nutrient foramen similar to *Kayentatherium* and *Morganucodon* [70,74]. Directly lateral to the vascular foramen is a unique notch into the vertical margin of the prootic lateral flange that is not directly comparable with other cynodonts that may have housed the lateral head vein.

Posterior to the notch is the pterygoparoccipital foramen. It is almost entirely enclosed by the lateral flange anteriorly and the paroccipital process posteriorly. The squamosal overlaps the paroccipital process dorsolaterally so that it may be considered to contribute to the partial enclosure of the foramen. In *Kayentatherium* [70] and *Morganucodon* [74] the pterygoparoccipital foramen is open laterally, a derived condition compared to the completely enclosed foramen present in more basal eucynodonts. Although the pterygoparoccipital foramen of *Pseudotherium* is nearly entirely enclosed by the prootic and squamosal, the lateral flange and squamosal do not actually contact one another (Fig 19).

An external ascending groove rises dorsally from each pterygoparoccipital foramen and cuts between the prootic and the squamosal. Within the groove, and posterodorsal to the pterygoparoccipital foramen, is the entrance to the posttemporal canal, through which passed the arteria diploëtica magna [85]. The superior ramus of the stapedial artery fit within the ascending groove and entered inside of skull through a dorsal foramen, identified as the diploёtic foramen in *Brasilitherium* [22].

The petrosal encapsulates the inner ear and is perforated by several foramina. It contacts the squamosal laterally, the exoccipital posteromedially, and the basioccipital anteromedially. The prootic canal is positioned posterior to the lateral flange vascular foramen, near the fenestra ovalis. The perilymphatic foramen, and the jugular and hypoglossal foramina share a common pit but are otherwise distinct. The jugular and hypoglossal foramina are posteromedial to the perilymphatic foramen and lie close together, but remain separate. The pars cochlearis is completely ossified within the petrosal and exposed ventrally, giving *Pseudotherium* a slightly convex promontorium (Fig 20). A promontorium is derived among eucynodonts. It is present in Mammaliaformes [86], and in the non-mammaliaform cynodont *Brasilitherium* [53].

The cochlea (Fig 21) is medially in-turned and is broadest posteriorly near the vestibule and narrows anteriorly towards its apex. Relative to the cochlea of more basal cynodonts including *Probainognathus* (see [87]), the cochlea of *Pseudotherium* is elongate, more comparable to *Brasilitherium* [53] and other basal mammaliamorphs [87,88]. The posterior vestibule bears a large anterior semicircular canal (SCC), a smaller posterior SCC, and a lateral SCC that is similar in size to the posterior. The anterior and posterior SCCs share a long common crus. Even the posterior and lateral SCCs appear to share a short common crus, which is swollen to accommodate the ampulla of the posterior SCC. The ampullae of the anterior and lateral SCCs meet laterally on the dorsum of the vestibule.

The internal auditory meatus is closed by a medial wall, which is pierced by four foramina to the inner ear (Fig 21A). Three are anteroventral to the subarcuate fossa. The anterior-most foramen is small and likely transmitted the facial nerve (VII). The facial nerve passed over the cochlea and exited the petrosal at the posterior end of the cavum epiptericum. The two more posterior foramina transmitted the cochlear and vestibular branches of the vestibulocochlear nerve (VIII). The wider of the two foramina opens at the neck of the cochlea, anterior to the vestibule, and transmitted the cochlear nerve. The smaller and more dorsal of the two foramina enters the vestibule just ventral to the ampulla for the anterior SCC and transmitted the vestibular nerve. Although the presence of a walled internal auditory meatus, with separated foramina for VII and VIII, is described as an apomorphy of Mammaliamorpha [1,14], it was more recently scored as present in *Pachygenelus monas* [24]. The fourth foramen is positioned ventral and slightly anterior to the subarcuate fossa. A digital endocast of the inner ear shows that this foramen marks the entrance to a short canal entering the vestibule immediately anterior to the base of the common crus, which is the position for the vestibular aqueduct in mammals [87].

The paroccipital process is bifurcated laterally into two distinct processes, as in other basal mammaliamorphs [1,15,81]. The posterior process protrudes ventrally. It is overlapped posteriorly at its base by a small process of the tabular. In ventral view, a small emargination between the posterior and anterior processes of the paroccipital process probably represents the homolog of the fossa for the stapedial muscle in therian mammals. The petrosal contacts the squamosal near quadrate notch. The larger anterior process of the paroccipital process is elongate, its lateral surface is partially in contact with the squamosal (Fig 22). The anterior process of the paroccipital process made contact with the quadrate [70], while the posterior process probably contacted the hyoid [1,81].

#### Orbitosphenoid

The μCT of the holotype are especially useful in visualizing the orbitosphenoid. In *Pseudotherium* the orbitosphenoid is ossified and positioned in its plesiomorphic position [89] just beneath the skull roof where it forms the primary walls of the endocranial cavity (Fig 23). This is similar to the configuration illustrated in *Probainognathus* [35,72]. In lateral view, it is just visible through the dorsum of the orbital fissure but is otherwise covered by the orbital process of the frontal. Although the orbitosphenoid is broken and shifted slightly out of anatomical position, its general shape is recognizable. It spans most of the length of the orbital fissure. In cross section, the orbitosphenoid is a rounded L-shape. The ventral legs of each “L” contact one another at the midline to form a U-shaped cross-section. Descending from this is a vertical median stem, identified in *Probainognathus* as the presphenoid by Crompton et al., [72]. The lateral surface of the orbitosphenoid is perforated by a large foramen, which may have enclosed the optic nerve. Unlike *Probainognathus*, in which the right and left optic foramina are confluent [72], they remain separate in *Pseudotherium* and penetrate the lateral orbitosphenoid wall near its center.

#### Alisphenoid

The alisphenoid forms the posterior margin of the orbital fissure. Dorsally, the tall alisphenoid overlaps the ventral edges of the frontal and parietal. The posterodorsal corner of the alisphenoid is overlapped by the anterior lamina of the prootic (petrosal). In basal cynodonts, the orbital fissure is a broad space between the orbital wall and alisphenoid. In tritylodontids and mammaliaforms, an ossified lateral wall that largely closes the orbital fissure is formed by contributions from the alisphenoid, orbitosphenoid, frontal, and in some taxa the palatine [32,70]. *Pseudotherium* is plesiomorphic in having a widely open orbital fissure.

A posteriorly directed process of the alisphenoid contacts the anterior margin of the prootic, separating the foramina for the maxillary and mandibular branches of the trigeminal nerve. The anterior margin of the alisphenoid is broken dorsally where it would have contacted the frontal. The pterygoid process of the alisphenoid overlies the lateral surface of the pterygoid adjacent to the border of the interpterygoid vacuity. Posteroventrally, the quadrate ramus of the alisphenoid makes contact with the lateral flange of the prootic. Its ventromedial surface is underlapped by the quadrate ramus of the pterygoid. Together with the quadrate ramus of the pterygoid, these two processes form the lateral boundary of the large cavum epiptericum (Fig 3).

#### Basisphenoid and parasphenoid

The endochondral basisphenoid and membranous (dermal) parasphenoid are fused into the parabasisphenoid complex, but the boundaries of the two components can be discerned. The basisphenod portion consists of a triangular, robust basipterygoid processe that contacts the pterygoid, a narrow central portion that is pierced by the carotid foramina and underlies the sella turcica and the pituitary fossa, and a broad posterior end that contacts the basioccipital posteromedially and the petrosal posterolaterally.

The parasphenoid consists of a long, anteriorly projecting rostrum that is “V”-shaped in cross section. In *Pseudotherium*, the short rostrum of the parasphenoid tilts dorsally towards the endocranial cavity where it passes above the interpterygoid vacuites. In life, it likely divided the interpterygoid vacuity and contacted the pterygoids where they form the primary palate, as in other derived cynodonts (see [35]). The ventral keel on the parasphenoid rostrum may have been continuous with the midventral keel on the primary palate and extended into the nasopharyngeal cavity in continuity with the cartilaginous nasal septum.

Behind the rostrum are the parasphenoid wings or alae, which flare laterally and underlap the ventral surface of the basisphenoid. The parasphenoid ala terminates at the anterior end of the pars cochlearis and does not participate in the border of fenestra vestibuli.

A median keel runs anteriorly from the basioccipital to the basisphenoid and along the ventral surface of the parasphenoid rostrum. Where the parasphenoid alae converge at the carotid foramina, the median keel deepens and forms a small crest (Fig 24). Although a similar crest was described as an autapomorphy of *Brasilitherium* [22], it may althernatively (in conjunction with several other poorly documented features–see Discussion), be a synapomorphy with *Pseudotherium*.

A well-developed parasphenoid ala is potential mammaliamorph synapomorphy that is lacking in *Probainognathus* but reportedly present and fused to the petrosal in tritylodontids such as *Yunnanodon* [87] and *Kayentatherium* [70]. In Mammaliaformes the wings are absent as discrete structures, and they are reduced or absent in the stem-mammaliaforms *Adelobasileus* [46] and *Sinoconodon* [90]. Reduction and loss of the parasphenoid alae is associated with elongation of the cochlea and its housing within an expanded bony promontorium [86]. *Pseudotherium* and tritylodontids differ in that the parasphenoid ala extensively covers the entire cochlear promontorium in tritylodontids while in *Pseudotherium* it terminates beneath the promontorium apex (Figs 3 and 21). *Pseudotherium* resembles *Brasilitherium* in that the promontorium is only partially covered by the parasphenoid ala (basisphenoid wing; [53]). The condition in *Pachygenelus monus* and *Riograndia guaibensis* are unknown.

#### Basioccipital

Although the basioccipital appears fused with the petrosal and exoccipitals, its borders are sufficiently distinct to warrant a separate discussion from the occiput as a whole. The basioccipital forms the posteroventral portion of the braincase and the ventral rim of the foramen magnum. A ventromedial keel arises anterior to the foramen magnum and spans the length of the basioccipital. Two deep pits occur on either side of a keel on the anterior end of the basioccipital. They may have served as the insertion for the anterior rectus capitis muscles [70]. Paired foramina are situated posteriorly and lateral to the perilymphatic foramina, as illustrated in *Morganucodon* [74]. Both foramina represent partitioning of the embryonic metotic fissure, which lies between the developing otic capsule and basioccipital, and transmitted the vagus nerve (cranial nerve X) and jugular vein [91]. In mammals, the metotic fissure closes and becomes partitioned in different ways and degrees, such that a single foramen may transmit both structures, or the fissure may be partly or completely divided [92] as it is in *Pseudotherium*.

The CT scans show the structure of the occipital condyles to be problematic. As in cynodonts ancestrally, the occipital condyle is paired [7], and in *Pseudotherium* the two condyles resemble the condition in *Thrinaxodon* [47] in which each is a small knob placed at the ventrolateral edge of the foramen magnum.

The basioccipital and basisphenoid are separated on the midline by a distinct gap. This feature is present in *Thrinaxodon* and other cynodonts and is referred to as an “unossified zone” presumably filled by cartilage [71]. In *Pseudotherium*, however, volume rendering of the skull reveals that the gap is partially bridged by a ventral median process of the basioccipital. This narrow finger-like process extends forward beneath the basisphenoid, crossing the unossified zone. This process seems unique to *Pseudotherium*. However it is only visible in the volume rendering (Fig 25); a surface rendering (Fig 25) fails to distinguish these structures and produces a basioccipital-basisphenoid contact that resembles drawings of *Brasilitherium*, where the unossified zone and median bridge may be covered by residual matrix.

#### Occiput

The occiput is broader than it is tall. Most of the individual bones in *Pseudotherium* are coossified and their boundaries indistinct, hence the occiput is described here as a single unit. It is partially roofed by the broad lambdoidal crests that rise dorsomedially and meet at a point with the overhanging sagittal crest. A short occipital crest runs vertically below the apex of the sagittal crest. The lateral margins of the supraoccipital are faintly visible, as well as the sutures between the basioccipital and the exoccipitals in the ventral margin of the foramen magnum. The supraoccipital, exoccipitals, and basioccipital comprise the dorsal, lateral, and ventral borders of the foramen magnum, respectively. In ventral view, the hypoglossal (XII) foramen pierces the exoccipital anterior to the condyle.

The tabular forms the lateral surface of the occiput and surrounds the posttemporal fenestra, which probably conveyed the lateral head vein [85]. Its medial boundaries are not superficially observable, but are evident in cross section. The tabular contacts the postparietal dorsally, the supraoccipital dorsomedially, the exoccipital ventromedially, the petrosal ventrally and anteriorly, and the squamosal laterally. The posttemporal fenestrae are low and centered between the foramen magnum and the lateral margins of the occiput. Ventral to each is a horizontal ledge that ends laterally in a process which overlaps the posterior process of the paroccipital process. There are four depressions on the occiput: two dorsomedial depressions on either side of the supraoccipital, and two ventrolateral depressions between the posttemporal fenestrae and the foramen magnum. These four depressions represent insertions of cervical musculature that elevates the head.

#### The dentition

Because the premaxillae and lower jaw of *Pseudotherium* are not preserved, the form and number of incisors are unknown. The upper canines are long and curved. The canines were displaced postmortem, and the fossil is distorted on its left side, further displacing the left canine. As a result, the long roots of the canines appear to erupt through the overlying maxilla where their roots are broken and eroded. The crown morphology of the canines is distinctive, being buccolingually compressed, and with ridges running nearly the length of the crown on both labial and lingual surfaces (Fig 26). These canine ridges are an autapomorphic feature of *Pseudotherium*. The relative size and shape of the canines *Pseudotherium* most closely resemble those of *Prozostrodon* which are also relatively long and bear a sulcus on their labial surface [75]. The canine crowns in *Brasilodon quadrangularis* and *Brasilitherium riograndensis* have not been described in detail. The canines of *Brasilodon* are described as transversely flat with a sulcus running dorsoventrally along the anterolateral surface, similar to *Prozostrodon* [18]. The canines of *Pseudotherium* and are longer and more curved than those of *Brasilodon* (see [17]). The complete canine crown is lacking in *Brasilitherium riograndensis*, precluding their comparison to *Pseudotherium*.

The palate is marked by deep paracanine fossae anteriomedial to the canines that would have received what must have been long lower canines when the jaws were closed. A small diastema separates the canines from the postcanines. If replacement in *Pseudotherium* was like that of other cynodonts, the presence of a diastema suggests that at least one postcanine tooth had been shed and went unreplaced [83].

The postcanines of *Pseudotherium* are sectorial and blunt. There are nine postcanines in the maxilla (Fig 27). The upper tooth row is complete on the left side of the skull, whereas postcanine (PC) 6 and PC9 are absent on the right. Crown complexity increases with each successively distal postcanine, with the exception of the ultimate postcanine, which is somewhat simpler in shape than the penultimate postcanine. All postcanines, except the first, have wear facets that mostly affect the main cusp (A) and are not similarly developed in all teeth. There is less wear in the smaller teeth (Fig 27A). The facets are flat with surfaces medioventrally oriented. PC1 is the simplest in form and its crown has a single cusp. The crown of PC2 comprises a large main cusp and one small distal cusp. Crowns of PC3 –PC6 comprise a relatively large central cusp A and mesial and distal cusps, B and C, respectively. The crowns of PC7 –PC8 bear accessory cusps. They are unusual in that the position of their accessory cusps subtly differs between the left and right side. The left PC7 has two small buccal accessory cusps on the mesial and distal sides of cusp C. The more mesial buccal accessory cusp is small enough that it is hardly observable. The right PC7 also has two accessory cusps near cusp C, however, they are more distally positioned, with the more mesial accessory cusp positioned distobuccal to cusp C, and the second accessory cusp positioned immediately distal to cusp C so it is in alignment with the three main cusps. Being the penultimate postcanine, PC8 (Fig 28) has the most cusps, comprising three buccal accessory cusps distally. Of the distal accessory cusps for PC8, two are buccal to cusp C (cusps D and E) while the third is immediately distal to cusp C (cusp F). Only the left PC9 is preserved. Three main cusps are distinguishable, and though the shape of the crown resembles PC7 in occlusal view, no distal accessory cusps are visible. It is difficult to determine if PC9 lacks accessory cusps or if the accessory cusps were worn away.

The crown morphology of the upper postcanines resembles that of other derived probainognathian cynodonts. The postcanines of *Pseudotherium* differ from tritheledontids, whose postcanines have a transversely broad central cusp with smaller offset mesial and distal cusps. The upper postcanine morphology is more comparable to that seen in *Botucaraitherium* and the brasilodontids, which similarly bear three mesiodistally symmestrical cusps. In *Botucaraitherium*, the crown is bulbous with two mesio-buccal accessory cusps and one distal buccal accessory cusp [26]. In *Brasilodon* and *Brasilitherium*, there is one mesial and one distal accessory cusp on the buccal surface [18,21]. *Pseudotherium* differs from these three taxa in that the majority of its postcanines lack accessory cusps, except for PC7 and PC8. The two postcanines that do have accessory cusps have them positioned distobuccally around cusp C, as opposed to near both B and C. In addition to cusp number and orientation, their reduced and blunt shape set the postcanine crown morphology of *Pseduotherium* apart from other cynodonts.

The last four postcanines (PC6 –PC8) differ from the preceding postcanines in their orientation within the maxilla. While the first five postcanines are oriented mesiodistally along the length of the snout, the last four postcanines are oriented with their mesial ends directed lingually and their distal ends directed buccally.

The roots of PC3 –PC9 are incompletely divided and form a figure eight in cross section. The pulp cavity of the postcanines is buccolingually compressed but undivided throughout its length (Fig 27C). On the left, PC4, PC7, and PC9 are higher in the maxilla than the other postcanines in the tooth row. On the right, PC4 and PC7 have shorter crown height, and PC6 and PC9 are absent. There are no replacement teeth in the maxillae, except at the base of the roots of both PC4s (Fig 29).

#### Postcanine orientation and root constriction

The extreme reductions of upper postcanine size and crown complexity are autapomorphic in *Pseudotherium*, but they resemble the postcanines of other eucynodonts in both orientation and root structure. The more distal upper postcanines (PC6-PC9) are mesiolingually in-turned so that the distal end of one postcanine is actually buccal to the mesial end of the proceeding tooth. Mesiolingually oriented upper postcanine crowns, termed “imbricating” by previous authors (e.g., [15]), has been described and/or illustrated for *Lumkuia fuzzi* [30], *Ecteninion lunensis* [45], *Therioherpeton cargnini* [75,93], *Pachygenelus monus* [35], *Riograndia guaibensis* [32,33], *Chaliminia musteloides* [35], *Diarthrognathus broomi* [25], *Tritheledon ricoini* [25,86], *Brasilodon quadrangularis* [18]; and *Brasilitherium riograndensis* [18]. The lower postcanines of *Brasilitherium riograndensis* are also mesdiolingually inturned [18]. This extensive list suggests that the mesiolingual orientation of the postcanines is apomorphic of probainognathian cynodonts, or perhaps a more inclusive clade.

The incompletely divided roots in *Pseudotherium* are of particular interest since Mammaliamorpha is diagnosed by teeth with completely divided roots [1,7,15]. As with postcanine orientation, incompletely divided roots have been documented in numerous other probainognathian taxa. These include *Microconodon tenuirostris* [94], *Therioherpeton cargnini* [75,93], *Prozostrodon brasiliensis* [75], *Pachygenelus monus* [95], *Riograndia guaibensis* [32], *Irajatherium hernendezi* [36], *Botucaraitherium belarminoi* [26], *Brasilodon quadrangularis* [17,18], and *Brasilitherium riograndensis* [17,18,22]. Though the postcanine roots of *Pseudotherium* are constricted, the dental canal for the nerve roots and dental pulp is not divided. Unfortunately not all of the dental canals of eucynodonts with incipiently divided postcanine roots have been described and/or illustrated, an observation that generally requires CT scanning or broken cross-sections of the roots. The dental canals of *Brasilitherium* [17] and *Botucaraitherium* [26] were reported as divided (although the canals within each lobe of the root appear connected for some teeth, e.g., [26]), which differs from *Pseudotherium*.

The distribution of crown orientation and root character states among probainognathians are summarized in Table 1. The important point is that the two characters are independent from one another. It is possible to have mesiolingually oriented crowns with single, non-constricted roots; conversely, it is possible to have incompletely divided roots of postcanines that are not in-turned. Of the taxa where the postcanine orientation and root shape are both known, in-turned crowns and constricted roots occur in two thirds of those taxa.

**Table 1:** Postcanine morphology in derived probainognathians.

Taxon	Postcanine crowns oriented anterolingual-posterobuccal	Incompletely divided roots	Citations
*Lumkuia fuzzi*	yes	?	Hopson and Kitching, 2001
*Ectenion lunensis*	yes	yes	Martínez et al., 1996
*Microconodon tenuirostris*	?	yes	Sues, 2001
*Therioherpeton cargnini*	yes	yes	Bonaparte and Barberena, 1975, 2001
*Prozostrodon brasiliensis*	yes	yes	Bonaparte and Barberena, 2001
*Pachygenelus monus*	yes	yes	Shubin et al., 1991; Martinelli and Rougier, 2007
*Riograndia guaibensis*	yes	yes	Bonaparte et al., 2001; Soares et al., 2011
*Irajatherium hernandezi*	yes	yes	Martinelli et al., 2005; Oliveira et al., 2011
*Chaliminia musteloides*	yes	No–single, unconstricted root	Martinelli and Rougier, 2007
*Diarthrognathus broomi*	yes	?	Martinelli and Rougier, 2007
*Tritheledon ricoini*	yes	No–single, unconstricted root	Broom, 1912; Martinelli and Rougier, 2007
*Botucaraitherium belarminoi*	no	yes	Soares et al., 2014
*Brasilodon quadrangularis*	yes	yes	Bonaparte et al., 2005
*Brasilitherium riograndensis*	yes	yes	Bonaparte et al., 2005, 2013
*Pseudotherium argentinus*	yes	yes	Wallace et al., 2017

## Discussion

### Phylogenetic analysis

We conducted a phylogenetic analysis to estimate the relationship of *Pseudotherium* to other probainognathian cynodonts. We modified a morphological character matrix for cynodonts that was initially assembled by Liu and Olsen [23], with subsequent modifications to character definitions and character state assignments by Soares et al. [26] and Martinelli et al. [24,25]. The Martinelli et al. [24] matrix is the most current matrix for the taxa of interest here, and its use enables the most direct comparisons to recently published results. Our character list and modifications of previous versions of the matrix are presented in S1 Appendix.

Three taxa were added to the Martinelli et al. [24] matrix. *Pseudotherium* was added and scored based on observations from CT data, from 3D printouts, and from the specimen itself. *Brasilodon* and *Brasilitherium* were synonymized in the Liu and Olsen [23] matrix, but a subsequent detailed description of a relatively complete skull of *Brasilitherium* supports two distinct genera [22]. *Brasilodon* and *Brasilitherium* were scored as separate taxa in the analyses by Soares et al. [26] and by Martinelli et al. [24,25]. We accept this conclusion and include *Brasilitherium* and *Brasilodon* as separate taxa. Because the monophyly and membership of ‘Brasilodontidae’ is controversial, *Botucaraitherium* [26], a possible brasilodontid, is also included in the analysis.

The final matrix has 34 taxa and 145 morphological cranial, dental, and postcranial characters (S2 Appendix). Of these, 119 are cranial and 16 are postcranial. Taxa range from 14% to 99% complete (based on 145 scorable characters), with an average completeness score of 75% (S3 Appendix). The matrix was analyzed in PAUP* 4b10 [96] using the parsimony algorithm. A heuristic search was performed for 1000 replicates (random addition sequence) with TBR (tree bisection and reconnection) branch swapping. Multistate characters were unordered, and ‘inapplicable’ characters were treated as missing data. Character state distributions are reported below using the DELTRANS optimization (S4 Appendix). The parsimony analysis resulted in eight most parsimonious trees (MPTs) consisting of 443 steps, with a consistency index (CI) of 0.4695, and a retention index (RI) of 0.7814.

Bremer support values, also known as Decay Indices, were calculated by a series of manual PAUP converse constraint analyses. Whereas the expected average Decay Index (tree length divided by number of internodes; [97]) for internodes under this matrix and tree topology is 17, most of the internodes had decay indices of less than five, and the Consistency Index (CI) is 0.47, indicating a high degree of homoplasy with this tree topology. The low Consistency Index is partly a result of homoplasy distributed across the non-probainognathian part of the tree, but most of the individual characters supporting relationships among probainognathians also have CIs of less than 1.

A strict consensus of eight most parsimonious trees (Fig 30) consistently places *Pseudotherium* within Probainognathia. The analysis also reveals that the greatest phylogenetic ambiguity lies in the relationships among non-probainognathian cynodonts, but discussion of those issues is beyond the scope of the present analysis.

Within probainognathians, our analysis had several interesting results. First, there is weak support for a clade that includes *Pachygenelus* and *Riograndia*, and we provisionally restrict the name Tritheledontidae to that clade. It is diagnosed by reduction in numbers and size of the incisors and canine, and loss of the paracanine fossa (S4 Appendix). *Therioherpetron*, which is widely considered a tritheledontid [32] (but see [24] for discussion) falls outside that clade. The node containing *Therioherpeton* and mammaliamorphs is weakly supported, with a Decay Index of 1. *Therioherpeton* is only 35% complete, while *Riograndia* is 74% complete and *Pachygenelus* is 93% complete. The sister taxon to this clade (Mammaliamorpha + Tritheledontidae + *Therioherpetron*) is *Prozostrodon*, which is only 46% complete. The incompleteness of *Therioherpetron* and *Prozostrodon* lack postcranial remains, and their crania are not thoroughly described. *Therioherpetron* and *Prozostrodon* thus complicates the diagnosis of Mammaliamorpha. All of the taxa just mentioned are known from specimens that include cranial remains, however, and could potentially contribute far more information to the analysis if they were CT scanned.

Secondly, the analysis found no support for a monophyletic Brasilodontidae, even though character data that was not incorporated into the matrix offers support for a sister taxa relationships between *Pseudotherium* and *Brasilotherium* (below). It did recover a topology in which *Sinoconodon* followed by *Adelobasileus* were consecutive sister taxa to Mammaliaformes. This clade (Mammaliaformes + *Sinocondon* + *Adelobasileus*) was grouped in an unresolved polytomy with *Brasilodon* and *Brasilitherium*. Lying outside this polytomy is *Botucaraitherium*, in a weakly supported node with a Decay Index of 1.

Thirdly, the analysis consistently found a well-supported monophyletic Tritylodontidae (Decay Index ≥ 5). *Pseudotherium* was consistently resolved within Mammaliamorpha, as the sister taxon to Tritylodontidae. Four unambiguous synapomorphies support this phylogenetic position, but the Decay Index for this node is 2. Two of its supporting characters represent character state reversals, and the other two were found to be homoplastic within Mammaliamorpha.

The membership of Mammaliamorpha is well-supported, based on six unambiguous synapomorphies, none of which represent homoplasy or character state reversals. Whereas the membership of Mammaliamorpha was strongly supported by this and other analyses (e.g., [1,15,44,62]), the larger hierarchy of character distributions beyond the variation captured in the Martinelli et al. [24] data matrix leaves a measure of doubt about the overall strength of support for considering *Pseudotherium* as either the sister taxon to tritylodonts or as a member of Mammaliamorpha.

### Additional phylogenetic context

Owing to the low Decay Index and low Consistency Index found in our analysis, and because of the effects of character incompleteness, it is important to look beyond the data matrix to additional information in the literature on cynodont skeletal evolution, as well as unpublished CT scans of relevant taxa to assess the phylogenetic position of *Pseudotherium*. Once a broader comparative sample of CT scans is more fully evaluated and published, we expect that an entirely new and much more informative matrix can be developed that will offer more stable resolution and support for the placement of *Pseudotherium* and other probainognathian taxa.

We also note that *Pseudotherium* and the other taxa of interest display a special kind of transitional mammalian characters. These are features such as the complex pattern of pterygopalatine troughs and ridges around the choana or the bifurcation of the paroccipital process, that are seen in the earliest fossil members of crown Mammalia, but that are subsequently so entirely transformed that nothing quite like them is found in extant mammals. Indeed, some of the differences in conceptualization of characters and character states found among the different data matrices that have been compiled over the last three decades for the Late Triassic cynodonts is related to there being no clear modern anatomical analogs. A better anatomical interpretation of these features will undoubtedly clarify the precise sequence of events that led up to the origin of Mammalia.

Some of these characters summarized below suggest that *Pseudotherium* lies either just outside of Mammaliamorpha or just inside, in an unresolved position at the base of Mammaliamorpha (instead of being the sister taxon to tritylodontids). In addition, several characters suggest that *Pseudotherium* forms a clade with *Brasilitherium* that lies just inside or just outside of Mammaliamorpha, and provide weak support for a monophyletic, but less inclusive Brasilodontidae.

### Mammaliamorph characters that are lacking in *Pseudotherium*

Although our formal analysis found *Pseudotherium* to lie within Mammaliamorpha, we note that it lacks a number of features that have been considered diagnostic of Mammaliamorpha in other analyses (e.g., [1,8,11,14,15,44,62]). Such features include several diagnostic derived character states that are present in Tritylodontidae and basal Mammaliaformes, but which are lacking in *Pseudotherium*. For example, *Pseudotherium* retains vestigial prefrontal and postorbital bones, which are entirely absent within Mammaliamorpha [1,7,15]. In the palate, *Pseudotherium* lacks the anterior extension of the ventral pterygoid keel onto the vomer, as is seen in tritylodontids [70] and in *Morganucodon* [74] and other mammaliaforms. *Pseudotherium* lacks fully divided postcanine tooth roots, another condition generally considered diagnostic of Mammaliamorpha. Additionally, *Pseudotherium* has an ossified medial orbital wall (as in mammaliamorphs), but this wall fails to extend posteriorly to enclose the orbital fissure behind the orbit. The orbital fissure in basal Mammaliamorpha is almost completely closed by the orbitosphenoid and alisphenoid [1,72]. *Pseudotherium* also lacks a floor beneath the cavum epiptericum (which held the trigeminal ganglion), which is at least partially present in tritylodontids, and fully present in Mammaliaformes [1,15].

### Additional characters *Pseudotherium* shares with Mammaliamorpha

The above plesiomorphies notwithstanding, *Pseudotherium* shares a number of derived character states widely recognized as diagnostic of Mammaliamorpha (e.g., [1,8,11,15,44,62]). The presence of such features in *Pseudotherium* may indicate that these character states are more widely distributed than previously believed, that they may be homoplastic, or that their distribution is equivocal because of incompleteness of some of the other relevant taxa. In several cases, these features can only be identified with certainty from CT scans.

Probably the most significant resemblance *Pseudotherium* shares with mammaliamorphs is in its cranial endocast (Wallace et al., in prep), in which the cerebral hemisphers form tall, elongated domes separated by a deep interhemispheric sulcus. Comparable endocasts are now known in tritylodontids (Wallace et al., in prep) and *Brasilitherium* [60]. *Pseudotherium* shares with Mammaliamorpha ossification of the orbital wall (anterior portion of the orbital fissure), in which sheets of bone from the frontal and palatine join to provide a solid orbital wall (although it fails to fully close the orbital fissure behind the orbit). The taxa that are sometimes grouped together as tritheledontids (*Therioherpeton*, *Pachygenelus*, *Riograndia*) preserve a more plesiomorphic condition in which both the orbital wall and orbital fissure remain broadly open. *Pseudotherium* also shares with mammaliamorphs the loss of an intact postorbital arch that separates the orbit from the temporal fenestra (although *Pseudotherium* retains a vestigial postorbital bone).

As in mammaliamorphs, *Pseudotheriuim* has a secondary palate that extends to the back of the tooth row. The arrangement of bones surrounding the choana takes on a distinct configuration in which parabasisphenoid and pterygoid no longer form a single continuous ventral parasagittal ridge, and instead form parallel parasagittal ridges (pterygopalatine ridges) separated by a shallow trough which may mark the passage of the auditory (eustacean) tube from the nasopharynx to the middle ear [73]. Broad parasphenoid alae are also present in *Pseudotherium* and in basal mammaliamorphs. The condition of these characters in tritheledontids has not been reported, but should be observable in CT scans.

In the otic region, the internal auditory meatus is walled medially with separate foramina for the vestibular and cochlear nerves, and the cochlea is slightly elongated, much like the condition in tritylodontids and *Morganucodon* [11]. A walled internal auditory meatus was scored as present in *Pachygenelus monas* [24], but we have not been able to confirm its presence and the geometry of its cochlea has not been described. Adjacent to the otic capsule, the prootic, alisphenoid, and quadrate ramus of the pterygoid join to form a laterally directed flange near the rear edge of the trigeminal foramen, and the paroccipital process is directed laterally (instead of ventrolaterally) and is bifurcated distally, with one distal process forming a separate condyle for a kinetic articulation with the quadrate, and the other apparently articulating with the hyoid. The basicranium is also broadly expanded to widely separate the pterygoid transverse processes. As in *Kayentatherium* [70] and *Morganucodon* [74] the pterygoparoccipital foramen is open laterally, a derived condition compared to the completely enclosed foramen found in more basal cynodonts.

Yet another feature of the otic region *Pseudotherium* shares with other mammaliamorphs includes the fusion of the prootic and opisthotic elements into a single petrosal. The petrosal has been described in triytlodontids, and tritheledontids, as well as in *Brasilitherium* [1,41,53,70,86,98]. In CT slices of *Pseudotherium*, internal sutures between the prootic and opisthotic are not observable since the elements have fused. However, some surface features between the two elements on the crista interfenestralis suggest that fusion of the endochondral bones may have begun internally in early life, and progressed superficially until complete fusion of the petrosal was achieved. How the development of the petrosal compares across mammaliamorphs is undescribed, but the formation of the petrosal in *Pseudotherium* is unambiguous and may likely be a synapomorphy shared with other mammaliamorphs. If *Pseudotherium* is a stem mammaliamorph, the presence of a petrosal in *Pseudotherium* would be its oldest occurrence in probainognathian cynodonts presently known.

### Comparison to ‘brasilodontids’

*Pseudotherium* shares several derived similarities, most notably in the nasal cavity and ear, to *Brasilitherium*, and possibly also to *Brasilodon*. In an earlier CT study of *Brasilitherium*, thin, fragile bony elements within its nasal cavity were interpreted as fragments of a partially ossified nasoturbinal and first ethmoid turbinal [54]. The posterodorsal end of the nasal septum was also reported as partially ossified to form a mesethmoid, all of which support olfactory epithelium in mammals. In *Pseudotherium*, one of the ossified elements lies in the general location of the mammalian maxilloturbinal. It is most clearly visible on the left side as a C-shaped structure of thin bone, but it preserves no bony attachment to the maxilla, floating freely inside the matrix of the nose. A similar free-floating structure is illustrated in *Brasilitherium* in a similar position [54].

These structures are exceedingly small compared to even the smallest turbinals known in mammals, and it is instructive to examine them in light of the pattern in which turbinals develop in mammalian ontogeny, as they become the supporting skeleton of the respiratory and olfactory epithelium (reviewed in [5,52]). The olfactory epithelium begins its development on the inner walls of the cartilaginous embryonic nasal capsule, as olfactory receptor genes are expressed. In mammals the nasal capsule becomes extensively ossified and within it grows an elaborate labyrinth of thin bony struts known as “ethmoid turbinals” (or turbinates). Epithelial growth quickly exceeds the surface area of the nasal capsule walls, causing the epithelium to fold into the lumen of the capsule. Each epithelial fold is supported by a transient cartilage that grows apically into the fold from the nasal capsule wall, but at no time is there an extensive, stand-alone cartilaginous skeleton (as is the case in some birds). The growing cartilage is quickly replaced by rigid perichondral bone that forms the mature ethmoid turbinals. Growth of the olfactory epithelium and its turbinals begins adjacent to the main olfactory bulb and proceeds rostrally. As they grow, the turbinals widen rostrally, branching and interleaving in intricate patterns that eventually occupy a large volume of the nasal space. The mature olfactory epithelium is confined to the dorsal and caudal regions of the nasal chamber, where the turbinals form numerous recesses into which volatile odorant molecules are received and stimulate odorant receptors. The turbinals subdivide the nasal chamber, maintain spatial integrity of its epithelia, and the spatial zonation of olfactory receptors. The number of functional olfactory receptor genes correlates most strongly with mature olfactory epithelial surface area [99]. The ossification of ethmoid turbinals in the ancestral mammal supported the expansion of the surface area of its olfactory epithelium by an order of magnitude over nasal chambers lacking such structures [52].

**Fig 31:**
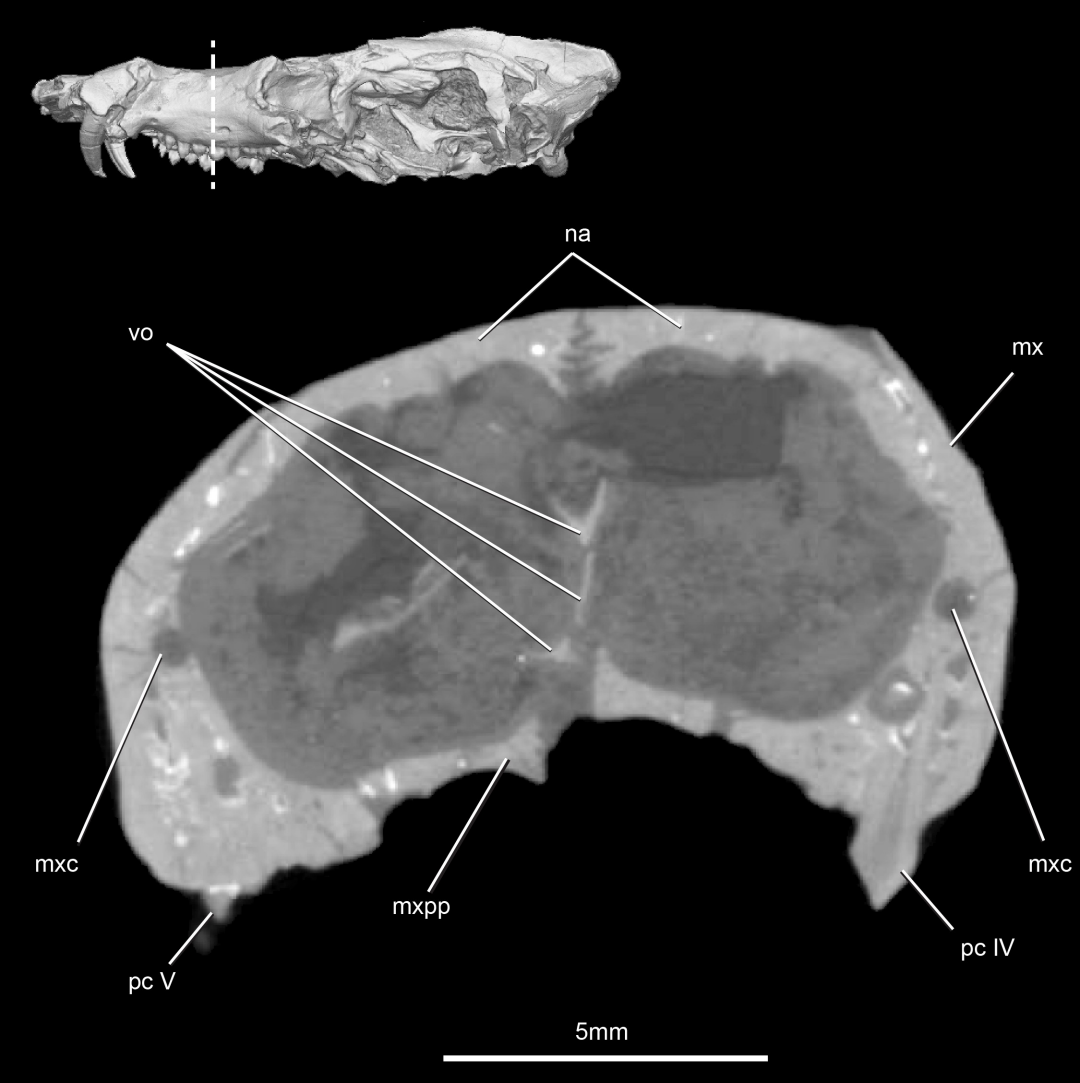
*Pseudotherium argentinus*, the tall plesiomorphic vomer. Line through *Pseudotherium* rostrum indicates position of cross sectional slice. Abbreviations: mx, maxilla; mxc, maxillary canal; mxpp, maxillary palatal process; na, nasal; pc IV, postcanine tooth IV; pc V, postcanine tooth V; rpc IV, replacement postcanine tooth IV; vo, vomer.

Additionally, the vomer in both *Pseudotherium* and *Brasilitherium* is plesiomorphic in being Y-shaped with a long vertical stem that is at least half the height of the nasopharyngeal cavity (Fig 31), restricting olfactory space to the dorsal half of the nasopharyngeal chamber [70]. This is the same condition found in *Thrinaxodon* [47,100]. The groove in the top of the Y-shaped vomer supported the cartilaginous internasal septum [72], which partially ossifies in Mammalia to become the mesethmoid [52]. In mammals, the mesethmoid is tall the ‘stem’ of the vomer is reduced or absent. The mammalian vomer is now V-shaped, and the ossified mesethmoid rises above it nearly the entire height of the nasopharyngeal passage [7,52].

CT scans of two tritylodont specimens reportedly preserve slight ossification of the rear part of the nasal capsule [11]. However, a lack of contrast between bone and matrix in these specimens leaves interpretation of the CT data equivocal. No similar structures have been observed in CT scans of taxa more closely related to mammals, including *Morganucodon* and *Hadrocodium* [2] and our unpublished datasets for other tritylodonts. Moreover, if not simply artifacts, a strict interpretation based on phylogenetic analysis (below) resolves these structures as synapomorphies that link *Pseudotherium* and *Brasilitherium*, to the exclusion of all other taxa of interest to this analysis. The evidence that these small bones represent enhancement of the olfactory system is enticing, but not decisive (see [72]) and underscores the desirability of CT scanning more of these small Mammaliamorph specimens.

*Pseudotherium* further resembles *Brasilitherium* in that the promontorium is only partially covered by the parasphenoid ala (= basisphenoid wing; [53]). Both *Pseudotherium* and *Brasilitherium* have a crest between the promontorium and the trough on the prootic, and both taxa have a distinct and gracile crista interfenestralis. A median keel runs anteriorly from the basioccipital to the basisphenoid and along the ventral surface of the parasphenoid rostrum. Where the parasphenoid alae converge at the carotid foramina, the median keel deepens and forms a small crest (Fig 24). Such a crest has only been described in *Brasilitherium* and was thought to be autapomorphic for that taxon [22].

*Brasilitherium* and *Pseudotherium* also have a ventral process on the basisphenoid, a feature of the basisphenoid that is also described for *Brasilodon* [17]. The process in *Brasilodon* continues posteriorly as a low crest bordered by two longitudinal depressions. *Pseudotherium* also has a ventral process of the basisphenoid that continues as a crest that is bordered by depressions. *Brasilitherium* and *Pseudotherium* are the only known taxa to retain prefrontal and postorbital bones, while lacking a complete postorbital arch. Both taxa have nine postcanine teeth with reduced crowns with indistinct cusps. Finally, the thin, fragile C-shaped ossifications in the nasal capsule in *Pseudotherium* and *Brasilitherium* may be ambiguous in terms of their identity and function, but their position and form are similar. Given this mosaic of features, there is reason to continue to test whether or not some or all of the taxa variously assigned to ‘Brasilodontidae’ indeed form a clade, and whether any of these taxa lie inside or just outside of Mammaliamorpha.

## Conclusions

The discovery of *Pseudotherium argentinus* underscores the diversity of small cynodonts in the mid- to Late Triassic and highlights the acquisition of growing numbers of mammalian features as a distinctive feature of this radiation. Although the phylogenetic position of *Pseudotherium* is not fully resolved, it shares with the other ‘taxa of interest’ a number of novelties that link it closely to the origin and early diversification of Mammaliamorpha. Current evidence suggests that the evolution of endothermy, lactation, parental care, prolonged activity, and the beginnings of encephalization were the products of this segment of history, and that it played out in miniaturized cynodonts [5,7,10,11]. The current uncertainty on phylogenetic relationships among those taxa that have been referred to as tritheledontids and brasilodontids is based in part on incompleteness, and also on differing strategies for sampling taxa for analysis. It seems clear that resolving this phylogenetic ambiguity will more precisely elucidate the sequence of events culminating in the origin of Mammalia, and that CT may be a key technology in providing character evidence for this phylogeny.

The taxa of interest described above retain relatively much smaller brains than found in *Morganucodon* and other mammaliaforms. However, *Pseudotherium* and *Brasilitherium* provide evidence of a modest pulse in encephalization that preceded the much more marked encephalization pulse expressed in the last common ancestor of Mammaliaformes [2] (Wallace et al., in prep). In this respect *Pseudotherium* is further from Mammaliaformes than other Late Triassic and Early Jurassic fossils such as *Adelobasileus* and *Sinoconodon* that present a more advanced degrees of encephalization. Aspects of the nose, palate, dentition, the circumorbital configuration, and in elongation of the cochlea also take on a new measure of resemblance to mammals.

We find a weak signal that diagnoses a monophyletic tritheledontidae that includes *Pachygenelus* and *Riograndia*. A weak signal also links *Pseudotherium* and *Brasilitherium*, and with less confidence *Brasilodon*, in a monophyletic Brasilodontidae. While the available evidence seems favors excluding *Therioherpeton*, *Pachygenelus*, and *Riograndia* from Mammaliamorpha, it is less clear whether *Pseudotherium* and *Brasilitherium* lie just within or just outside of that clade. The slight increase in encephalization in *Pseudotherium* and *Brasilitherium* compared to tritylodonts and tritheledonts may indicate that brasilidontids lie within Mammaliamorpha, where they would represent the most basal members of the mammaliaform stem. As the specimens upon which these taxa are based are CT scanned, and as the scans are made available to the larger community, we expect improved phylogenetic resolution of this radiation of small cynodonts.

## Supporting information

S1 Appendix Character list. (DOCX)

S2 Appendix Character matrix. (DOCX)

S3 Appendix Completeness table. Completeness of taxa analyzed.
(DOCX)

S4 Appendix PAUP* output. (DOCX)
