# Supplementary material for: First record of a basal mammaliamorph from the early Late Triassic Ischigualasto Formation of Argentina
Source: PLoS One. 2019 Aug 7;14(8):e0218791. doi: 10.1371/journal.pone.0218791 (PMC6685608; doi:10.1371/journal.pone.0218791)
Supplement: S1 Appendix — (DOCX) [file pone.0218791.s001.docx]

**Phylogenetic Analysis**

The present phylogenetic analysis uses the Liu and Olsen [1] morphological character matrix for Eucynodontia, with some modifications from Soares et al. [2] , and Martinelli et al. [3,4]. Such modifications include the recognition of *Brasilodon* and *Brasilitherium* as distinct taxa; the scorings for these taxa follow the scorings of Soares et al. [2], with the exception of Character 13 pertaining to the prefrontal. Character state changes suggested by Soares et al. [2] were used because the authors’ justification for the character state changes were supported by the literature. The only exception was to the scoring of *Prozostrodon* for characters 13 and 14, which changed the character state from presence of prefrontal and postorbital by Liu and Olsen [1] to absence. Character 37, describing the relative length of the palatine in the secondary palate, was the only character change made by Soares et al. [2], that was not accepted since the change did not impact the scoring of any taxa. Most changes to the scorings of taxa by Martinelli et al. [3,4] were adopted in this analysis, except for those that came into conflict with the character changes made in the present study. For example, *Brasilodon* was rescored by Martinelli et al. [3] for character 11 to a state that has been removed in this study; therefore, that change was not accepted.

1. **Changes in character definition**

**Character 11.** Lateral expansion of braincase in parietal region: absent (0); well-developed (1).

The braincase was described as lacking any lateral expansion (0), having a moderately developed lateral expansion (1), or being well-developed (2) and had the option of being analyzed as an ordered character. State 0 would reflect the plesiomorphic, tubular brain of most eucynodonts, while State 2 would reflect the unambiguously encephalized cranium of *Moranucodon* and mammals. *Therioherpeton*, *Riograndia*, *Pachygenelus*, and *Brasilodon* were scored State 1 [1], with the implication that they exhibit an intermediate morphology in a trend toward increased encephalization. Later, brasilodontids were scored as having a well-developed, laterally expanded braincase [2], likely based on a *Brasilitherium* endocast study [5], the results of which are discussed in the anatomical description of *Pseudotherium*. *Morganucodon* has an unambiguously wide braincase relative to other eucynodonts, along with other cranial morphologies expected to accompany an enlarged brain including a low and lateral orbitosphenoid and floored cavum epiptericum (possibly the result of the lateral flange being pushed ventrally by the expanding brain in development). The comparably well-encephalized braincase of brasilodontids is dubious, and they lack those accompanying cranial morphologies. No definition has been provided for a plesiomorphic braincase width versus a moderate, or well-developed, laterally expanded braincase; discrepancies in identifying the brasilodontid braincase as moderately laterally expanded or well-developed suggests one is needed. After comparing figures of tritylodontids, tritheledonts, and brasilodontids, whose braincases appear comparable in width relative to snout width, there is no reason to have character states beyond a narrow braincase (0), and a well-developed, laterally expanded braincase (1), where non-mammaliaforms exhibit the primitive condition, and mammaliaforms exhibit the derived condition.

**Character 62.** Paroccipital process: undifferentiated (0); differentiated (1).

The paroccipital process character has been simplified from a three-state character to a two-state character. Morphological character matrices have described the paroccipital process as either undifferentiated, or differentiated into an anterior process and a bulbous posterior process (as is commonly described for tritylodontids), or a quadrate and mastoid process (scored for *Morganucodon*). For this analysis, this character was reduced to two states, describing a differentiated process and an undifferentiated process. In its original publication, the character was written as a two-state character, with states undifferentiated (0), or differentiated into a quadrate and mastoid process (1) [6]. The character has persisted in all subsequent phylogenetic analyses of eucynodonts. Eventually the character was modified to a more general absence (0) or presence (1) of bifurcation [7–9], but still remained as two states. In both the original publication, and in subsequent publications, other characters were written to describe the shape of the anterior and posterior processes of the bifurcated paroccipital process. A third character state was recently added to include the shape of the bifurcated processes within the one character [10]. The character states for the paroccipital process included undifferentiated (0), differentiated in quadrate and mastoid processes (1), or differentiated into anterior and posterior processes (2). The polarity of the character states has changed in subsequent publications (e.g., [1,11]), and the character state describing the anterior-posterior bifurcation has even been embellished to specify a “bulbous” anterior process [1], no doubt elucidating the intent to distinguish between the bifurcated paroccipital process of the tritylodontids from the bifurcated paroccipital process of mammaliaforms. For this analysis, the character was reduced to the original two-state condition for two reasons. First, the distinction between the anterior and posterior processes of a bifurcated paroccipital process in tritylodontids vs. the quadrate and mastoid processes in mammaliaforms is not adequately defined. Further, brasilodontids do have a bifurcated paroccipital process, but a bulbous anterior process does not describe the condition in brasilodontids, nor does the quadrate and mastoid processes seem to be applicable. Second, the quadrate and mastoid process state seems to only apply to taxa within Mammaliaformes. Because this analysis is investigating the relationship of non-mammaliaform eucynodonts, such a distinction is not relevant. Therefore, reducing the paroccipital process character to its original two-state condition is more conservative and reasonable for the scope of this project.

**Character 81.** Shape of squamosal articulated surface for mandible: small and

medially or anteromedially facing facet (0); wide, ventrally directed glenoid cavity

(1).

Originally, Character 81 was a three-state character statement and State 0 read “absent.” However, this state is not independent of Character 79, which describes the variation of craniomandibular joint composition. A quadrate/articular jaw joint (State 0 of Character 79) necessitates the absence of a mandibular articular surface on the squamosal. Further, Character 81 is a transformational character. “Absent” is not a shape and including it in a transformational character is a logical shortcoming [12]. Contingency coding, scoring inapplicable for taxa lacking the character, is recommended in this situation.

1. **Changes in scoring of taxa**

Character state changes from latest iteration of character matrix [4] for *Prozostrodon*, *Brasilodon*, and *Brasilitherium*

Character 13: *Prozostrodon* changes from 1 to 0 based on [13].

*Brasilitherium* changes from 1 to 0 based on [14].

Character 14: *Prozostrodon* changes from 2 to 1 based on [13].

Character 62: *Brasilodon* changes from 0 to 1 based on figs. 3, 5b, and 6 of [15].

*Brasilitherium* changes from ? to 2 based on CT images published in [5].

**Character List**

The following morphological character list was used in the present phylogenetic analysis of *Pseudotherium argentinus*. It uses the character list provided by Liu and Olsen [1], with changes to characters 96, 106, and 107 as suggested by Soares et al. [2]. Changes to characters 11 and 62 were made for the present analysis. Italicized acronyms indicate differences in how the character is written between present study and previous author(s). Acronyms and numbers refer to authors and their published character numbers: R, [6]; W, [16]; WH, [17]; LL, [18]; L, [7]; LC, [19]; M, [20]; H, [21]; LCS, [8]; B, [15]; S, [22]; MA, [23], BO, [9]; SH, [24]; A, [10]; LO, [1]; SMO, [2]. “#” before the character indicates that character is ordered in some analyses.

*Rostrum*

1. #Premaxillary extranasal process: absent or with very little exposure (0); large but

not contacting nasal (1); contacting nasal (2). [*R2*, *W36*, L82, M14, A0]

2. Septomaxilla facial process: long, far beyond the posterior border of the external

nares (0); short, almost limited in the external nares (1). [*S1, A1*, LO2]

3. #Snout in relation to the temporal region (to the posterior border, not the parietal

crest): longer (0); subequal (1); shorter (2). [*A10*, LO3]

4. Paracanine fossa in relation to the upper canine: anteromedial (0), medial or

posteromedial (1), anterior (2), paracanine fossa absent (3). [A13]

5. Premaxilla forms posterior border of the incisive foramen: absent (0), present (1).

[M19, H1, B21, BO27, MA24, *A12,* LO5]

6. Maxillary platform lateral to the teeth series: absent (0); present (1). [M15, H77,

BO15, *A22*, LO6]

7. Maxilla: excluded from (0), or participates in (1) border of subtemporal fenestra.

[R15, W14, *L62*, M16, A20]

*Skull roof*

8. Profile of skull roof: nearly flat (0); remarkably concave (the parietal crest is

higher than the extension of anterior surface) (1); convex (the parietal crest is

lower than the extension of anterior surface) (2). [*S7, A64*, LO8]

9. Parietal foramen: present (0); absent (1). [R8, W12, LL34, L64, M31, H7, B24,

BO34, MA28, A6]

10. Interparietal (postparietal) in adult: separate bone in adult (0); fused with other

bones (1). [R21, W15, *LL36*, M34]

11. #Lateral expansion of braincase in parietal region: absent (0); well-developed (1).

[*L67, M33*, *LO11*]

12. Parietal crest posteriorly extending close to or reach the posteriomost position of

the occipital crest: absent (0); present (1). [LO12]

*Orbital region*

13. Prefrontal: present (0); absent (1). [R4, W1, M28, H3, B22, BO30, MA25, A3]

14. #Postorbital bar and postorbital: present (0); postorbital present but not forming

postorbital bar (1); both absent (2). [*R7, W2, LL33, L55, M29, H5, B23, B40,*

*BO31, BO32, MA 50, A5*, LO14]

15. #Palatine: do not meet the frontal (0); meets frontal but two elements without

significant contribution to medial orbit wall (1); meets frontal and two elements

with significant contribution to medial orbit wall (2). [*R6, R31, W17, W37, L56,*

*L60, M24, M30, H23, B29, BO46, MA38, A62*, LO15]

16. Sphenopalatine foramen: absent (0); present (1). [*L57, M26*, LO16]

*Zygomatic arch*

17. Zygomatic arch dorsoventral height relative to skull length: moderately deep

(10~18%) (0); very deep (>18%) (1); slender (2) (<10%). [*R16, W40, L54, M39,*

*H18, S5, BO40, MA33, A68*, LO17]

18. The anteroventral corner of the zygomatic arch: lie at the same level as (0); or

remarkably higher than (1) the postcanine line. [LO18]

19. Infraorbital process: absent (0); suborbital angulation between maxilla and jugal

(1); descending process of the jugal (2). [*M18*, *H21*, *H41*, *A25*, *B38*, *BO29*, *BO44*,

*MA36*, *MA46*, A69, LO19]

20. #Zygomatic arch dorsal extent: below middle of orbit (0); above middle of orbit

but still level within orbit (1); beyond the upper border of the orbit (2). [*H19*, LO20]

21. Posterior extension of jugal along zygomatic arch: extending back near

quadratojugal notch of squamosal (0), extending back near squamosal glenoid (1), reduced and receding from glenoid (2). [*L28*, LO21]

22. The posteroventral process of jugal: low (0); high, forming more than half height of zygomatic arch (1). [*H20*, *BO43, A70*, LO22]

23. The width of temporal fossa: reach greatest near middle (0); same throughout or little change (1); strongly increase backward, the posterior width much bigger than the anterior width (2). [*H39*, *BO42, MA44*, A73, LO23]

24. Squamosal groove for external auditory meatus: without or with an incipient

depression (0); deep (1). [*M55*, *H22*, *B28*, *S18*, *BO45*, *MA37*, *A72*, LO24]

25. Posterior extension of the squamosal dorsal to the squamosal sulcus in zygomatic

arch: incipient (0); well developed (1) [*A71*, LO25]

26. The notch separating lambdoidal crest from zygomatic arch: shallow (0); deep, “V”-shape

(1). [*H43, S17, BO55, A74*, LO26]

*Palatal complex*

27. Palatine: excluded from subtemporal border of orbit (0); participates in

subtemporal border by displacing pterygoid posteriorly (1). [L58]

28. Vomer exposure in incisive foramen (at anterior ends of maxillae on palate):

present (0); absent (1). [*M21*, LO28]

29. Vomer: with (0) or without (1) vertical septum extending posterior to level of

secondary palate. [SH63]

30. Ectopterygoid: does not contact maxilla (0); contacts maxilla (1); absent (2).

[*R32*, *H9, S15, A19*, LO30]

31. Interpterygoid vacuity in adults between pterygoid flanges: present (0); absent (1).

[M27, H10, B25, BO35, MA29, A24]

32. Secondary palatal plate on maxilla reaches midline: absent (0); present (1). [H12,

S11, *A15*, LO32]

33. Secondary palatal plate on palatine reaches midline: absent (0); present (1). [H13,

S12, *A15*, LO33]

34. Posterior extent of osseous secondary palate: far from (0), close to or beyond (1)

rear upper postcanine row. [R30, W16, *L68*, M23, LCS40, *H14, B26*, *BO36*,

*MA30, A17*, LO34]

35. #The posterior end of secondary osseous palate relative to anterior border of orbit:

anterior (0); about equal level (1); posterior (2). [H15, B27, BO38]

36. Osseous palate extension: 45% of skull length or less (0); more than 45% of skull

length (1). [A16]

37. Contribution of palatine to osseous secondary palate: short (less than 1/3) (0);

long (greater than 1/3) (1) [M22, *H40*, *B37*, *BO53*, *MA45, A18*, LO37]

38. Middle of pterygoid: smooth (0); a boss (1); a distinct median crest (2). [*LL12,*

*L71*, *A25*, LO38]

39. The nasopharyngeal roof posterior to the transverse process of pterygoid: narrow,

deep, ventrally forms a keel (0); wide, flat, the narrowest place greater than half

the width of the transverse process (1). [LO39]

40. Quadrate ramus of pterygoid: present (0); absent (1). [R38, W47, *LC10*, M40,

H30, B34, BO52, S20, MA43, A29]

41. Quadrate articulation with quadrate ramus of epipterygoid: absent (0); present (1).

[*LC11*, M53, *A30*, LO41]

*Basicranium, and lateral wall of the braincase*

42. Frontal-epipterygoid contact: present (0), absent (1). [*R39*, *W48*, *L61*, H35,

S24, A63]

43. Epipterygoid ascending process at level of trigeminal foramen: greatly expanded

(0); moderately expanded (1). [H32, B35, A66]

44. The anterior part of the basisphenoid: narrow (0); wide, and the width greater than

half the width of the transverse process (1). [*L69, LCS44*, LO44]

45. Parasphenoid ala: at the same level as the basicranium (0); ventrally expanded

below the basicranium (1). [*H17*, *BO39*, *MA32,* A28, LO45]

46. #Basisphenoid wing (parasphenoid ala): long, border the fenestra vestibuli (0);

slightly reduced and excluded from oval window, overlap the entire prootic

cochlear housing (1); greater reduced and overlapping a part of the pars cochlearis

(cochlear housing) (2); basisphenoid does not overlap the petrosal pars cochlearis

(3). [*R40, W49*, L74*, M41, M49, LCS37, A27*, LO46]

47. #Overlap of the basioccipital to the pars cochlearis: entire cochlear housing (0);

the medial side of the promontorium (1); no overlapping (2). [LCS 38]

48. Internal carotid foramina in basisphenoid: present (0); absent (1). [*R42*, *W50*,

*WH23*, LL14, L72, M45, H26, B31, BO48, MA40, A26]

49. Prootic and opisthotic: separated (0); fused at early ontogenetic stage to form

petrosal (=periotic) (1). [R51, W5, WH29, L34, BO56, A36]

50. Promontorium (Pars cochlearis of petrosal): absent (0); present (1). [R52, W6,

*LL1*, *L35,* LCS9, *BO57*, A34]

51. Internal auditory meatus: open (0); walled (1). [*R53, W7, WH12, L39, M47, H36,*

*B36,* A37]

52. #The trigeminal ganglion (semilular ganglion): open ventrally (0); partial prootic

floor (1); complete prootic floor (2). [W54, A33]

53. Lateral trough floor anterior to the tympanic aperture of the prootic canal and/or

the primary facial foramen: absent (0); present (1). [*R49, LL6, L43, M44,* LCS 15]

54. Vascular foramen in the posterior part of the lateral flange (Foramen “X” of

(Rougier et al., 1992) (p205)): absent (0); present (1). [*LL30*, *L53, M43*, LCS29]

55. Foramen and passage of prootic sinus on lateral trough: absent (0); present (1).

[*R50, W28, LL3, L45, MA49, BO58, A35*, LO55]

56. Route of the venous drainage exiting from the back of the cavum epiptericum:

only lateral flange vascular groove (0); absent (1); lateral flange vascular canal

present (foramina on lateral surface) (2). [W53, WH22, *H27*, LO56]

57. #Maxillary and mandibular branch (V2+3) of the trigeminal nerve exit: via single

foramen between prootic and epipterygoid (0); via two foramina between prootic

and epipterygoid (1); via separate foramina, some enclosed by anterior lamina of

prootic (petrosal) (2). [*L50, M48, H28, B33 BO51, S27, MA42, A65*, LO57]

58. Pterygoparoccipital foramen: squamosal does not contribute to enclosure of

foramen (0); squamosal contributes to enclosure of foramen (1); open (2). [*LL23*,

*L51*]

59. Vertical component of lateral flange of prootic (“L-shaped” and forming a vertical

wall to pterygoparoccipital foramen): absent (0); present (1). [*L52*, LCS25]

60. Anterior part of paroccipital process: the lateral aspect covered by the squamosal

(0); exposed due to dorsally withdrawn of the squamosal (1). [*L47*, LCS22]

61. Hyoid (stapedial) muscle fossa on the paroccipital process: absent (0); present (1).

[R55, W56, WH35, LL7, L40, M59, LCS32, MA48, BO61, *A38*, LO61]

62. Paroccipital process: undifferentiated (0); differentiated (1). [*R56, W18, L46, L47, M50, LCS21, LCS30, BO66*, *A43*, *LO62*]

63. Separation of fenestra rotunda and jugular foramen: confluent (0); completely and

widely separated (1). [R60, W29, *LL10*, *L42*, M46, *HK42*, LCS33, *B39*, *BO60*,

A40]

64. Articulation of the paroccipital process with the quadrate: absent (0); present (1).

[*R19, W41, M52, H29, A32*, LO64]

*Occipital region*

65. Paroccipital process in the base of the posttemporal fossa: absent (0); present (1).

[*H24, A44*, LO65]

66. Tabular: present (0), absent (1). [R22, LL19, L80, *LCS 47*, LO66]

67. The relationship of hypoglossal foramen (condylar foramen) with the jugular

foramen: confluent or sharing a depression (0); at least one foramen completely

separated from the jugular foramen (1). [*LL11, L75, M51, LCS39, BO65*]

68. Shape of the occipital condyles (in lateral view): bulbous (0); ovoid to cylindrical

(1). [*LL15, L77,* LCS51]

*Craniomandibular joint*

69. #Rotation of dorsal plate relative to trochlear axis on quadrate: less than 10 degree

(0); about 45 degrees (1); around 90 degrees (2); parallel to trochlear axis (3).

[L30, LC1]

70. Curvature of the contact facet on the posterior side of the dorsal plate of quadrate:

flat or convex (0); concave (1). [*L29, LC2, M56*, LO70]

71. Size of the lateral trochlear condyle relative to the medial trochlear condyle on

quadrate: the lateral condyle larger than the medial condyle (0); the medial

condyle equal or larger than the lateral condyle (1). [*LC3*, LO71]

72. Shape of the trochlear of quadrate: cylindrical (0); trough-shaped (1). [LC4]

73. #Lateral margin of the dorsal plate of quadrate: straight (0); flaring posteriorly

(1); flaring and rotated posteromedially (2). [LC5]

74. #Medial margin of the dorsal plate of quadrte: straight (0); flaring anteriorly (1);

flaring and rotated anterolaterally (2). [LC6]

75. Dorsal margin of dorsal plate of quadrate: retains pointed angle (0); has rounded

margin (1) [L31, LC7]

76. #Lateral notch and neck of quadrate (separation of the lateral margin of the

contact facet from the trochlear): the lateral notch is absent or poorly developed

(0); lateral notch developed, separating the lateral margin of the contact facet

from the lateral end of the trochlear (1); lateral notch is broader and separation of

the lateral margin of contact facet for the trochlear is wider, the lateral margin is

shifted medially (2); development of the neck with raise the contact facet away

from the trochlear (3). [*L32*, LC8]

77. Articulation of the quadrate with the squamosal: via concave recess in the

squamosal (0); covered dorsally by the squamosal (1); little or no contact with the

squamosal (2). [*WH7*, *LC12,* M54, *H31, A60*, LO77]

78. Articulation of the quadrate with the stapes: via a broad recess on the medial

margin and the median end of the trochlear (0); the stapedial contact restricted to

the medial end of the trochlear (1); via a projection from the medial margin of the

dorsal plate (2); via a medial vertical ridge in the neck (3); via a projection from

the neck of the quadrate (4). [*R20, W42*, *L33*, LC14]

79. Craniomandibular articulation; quadrate/articular (0); main quadrate/articular,

secondary surangular/squamosal (1); incipient dentary/squamosal (2); main

dentary/squamosal (3). [*R66*, *R67*, *W9*, *W60, L23*, *L24*, *M60*, *H25*, *LCS 70*, *B30*,

*S19*, *BO26*, *MA39*, *A58*, LO79]

80. Craniomandibular articulation: lies around the same height (0), much lower (1) or

remarkably higher (2) than the postcanine line. [*L25, A59*, LO80]

81. Shape of squamosal articulation surface for mandible: small and

medially or anteromedially facing facet (0); wide, ventrally directed glenoid cavity

(1). [*L26*, *B19*, *BO37*, *MA22*, *A57*, LO81]

*Mandible*

82. Dentary symphysis: unfused (0); fused (1). [R68, W10, L19, LCS56, H44, B17,

S34, BO21, MA21, A61]

83. #Lateral ridge of the dentary: absent (0); incipient (1); moderatly developed (2);

strongly projected (3). [A47]

84. Angle of the dentary: close to the position of postorbital bar (0); close to the jaw

joint (1). [*A54*, LO84]

85. Position of dentary-surangular dorsal contact relative to postorbital bar and jaw

joint: around midway (0); closer to jaw joint (1). [*H48, A55*, LO85]

86. Mediolateral thickening of the anterior margin of the coronoid process: absent (0);

present (1). [M66, H50, A51]

87. Splenial: large and deep, reaches ventral border of the dentary (0); reduced to thin

splint covering dentary groove (1). [M64]

88. #Postdentary bones: large, with tall surangular (0); angular, surangular, and

prearticular reduced in height and lying in dentary groove (1); further reduced to

single gracile rod in postdentary trough (2). [R74, W59, M65, H49]

89. Reflected lamina of angular posterior extent relative to distance from angle of

dentary to jaw joint: greater than 1/2 the distance (0); less than l/2 the distance (1).

[H51]

90. #Reflected lamina of angular shape: spoon-shaped plate with slight depressions

(0); hook-like lamina (1); reduced to thin process (2) [*M62*, *H52*, *S44*, A56]

91. Mandibular movement during occlusion: orthal movement during power stroke

(0); posteriorly directed power stroke (1); moderate rotation along the longitudinal

axis in power stroke (2). [*R79*, *W62, L2,* LCS74, *B2, BO2*, LO91]

*Dentition*

92. Postcanine occlusion: lack consistent contact relationship (0); bilateral,

interdigitating occlusion between multiple cusps (1); precise unilateral occlusion

(2) [*R84*, *R86*, *W33*, *L1*, *L14, M8, LCS 73, LCS 81*, *B1*, *BO1*, *MA1, A87*, LO92]

93. Relationships of wear facet to main cusp: wear facet absent (0); simple

longitudinal facet on crown (1); main cusp bears two distinct facets (2); multiple

cusps with each cusp bearing one or two transverse and crescentic facets (3).

[*L17, B16, MA19, BO20*, LO93]

94. Upper incisors number: five or more (0); four (1); three or less (2). [*R81, W63,*

*L5, M1, H53, B3*, S45, BO3, MA3, A76]

95. Lower incisor number: four or more (0); three (1); two or less (2). [L5, M2, H54,

B4, S46, BO4, MA4, A77]

96. Incisor size: all of similar size (0); some incisors large (1). [H56, *B5, B6*, *BO5*, *MA5*,

*MA6, MA7,* A78, LO96, SMO96]

97. Incisor cutting margins: smoothly ridged (0); serrated (1); denticulated (2). [*H55,*

*A79*, LO97]

98. Distinct upper incisor/canine diastema: present (0); absent (1). [*A81*, LO98]

99. Upper canine: large (0); reduced in size (<10% of skull length) (1); absent (2).

[*L6*, H57, A83]

100. Lower canine: large (0); reduced in size (1); absent (2). [L6, H58, A84]

101. Canine serrations: absent (0); present (1). [H59, A8]

102. Upper postcanine morphology: sectorial without or with incipient cingulum

broadening the crown (0); sectorial with a well-developed lingual cingulum (1);

bucco-lingually expanded (2). [*L13*, *M5*, *M9*, *H60*, *H62*, *A7*, *S51*, *S55, B10, BO8,*

*A89*, LO102]

103. #Anteriormost one-cusped tooth: present till adult (0); present only in juvenile

(1); absent (2). [LO103]

104. #Posteriormost gomphodont postcanine(s) in adults: absent (0); absent in juvenile

but present in adult (1); present from juvenile (2). [H80]

105. Posterior postcanines with strongly curved main cusp: absent (0); present (1).

[*S52*, A90]

106. Upper postcanine roots: single (0); constricted root, with longitudinal groove (1); divided into two longitudinal aligned roots (2); multiple roots (more than two) (3). [*R88, W65, W66, L9, M6, LCS77, B8, BO6, MA9*, *A95*, *LO106, SMO106*]

107. Lower postcanine roots: single (0); constricted root, with longitudinal groove (1); divided (2). [*R88*, W65, *L9*, M7, *B8, BO6*, *MA9, A94*, *LO107, SMO107*]

108. Buccal (external) cingulum on sectorial upper postcanines: absent (0); present (1).

[*R85, H61, B9, BO7, MA10, A91*, LO108]

109. Number of upper cusps in transverse row: one (0); two (1); three or more (2).

[H63, A9*2*]

110. Position of upper transverse cusp row on crown: midcrown (almost to posterior

margin) (0); on anterior half of crown (1); at posterior margin (no posterior

cingulum) (2). [H64]

111. Central cusp of upper transverse row: absent (0), midway between buccal and

lingual cusps (1); closer to lingual cusp (2). [H65]

112. Arrangement of main cups of upper postcanines: in single longitudinal row (0);

multiple cusps in multiple rows (1). [L13, LCS78]

113. Interlocking of lower postcanines: absent (0); distal cuspule ‘d’ of anterior molar

fits into embayment between cusp ‘b’ and cusp ‘e’ of the succeeding molar (1).

[L11, B14, BO18]

114. Number of lower cusps in transverse row: two (0), three or more (1). [*H73*, LO114]

115. Lingual cingulum on lower postcanine: present (0); vestigial or absent (1) [*L12*,

*LCS80, B11*, *B12*, *BO9*, *BO10*, *S56, A93*, LO115]

116. Lower posterior basin: absent (0); present (1). [H75]

117. Axis of posterior part of maxillary tooth row: directed lateral to subtemporal fossa

(0); directed toward center of fossa (1); directed toward medial rim of the fossa

and diverged (2); directed toward medial rim of the fossa and parallel (3). [*R80*,

*M12, H78*, *B13*, *MA17*, *MA20*, *BO14*, *BO16*, *BO17, A86*, LO117]

118. Upper tooth series posterior extension: below the orbit and anterior to the

subtemporal fenestra (0); anterior to the orbit (1); behind the anterior border of the

subtemporal fenestra (2). [*H79, A75*, LO118]

119. Postcanine replacement pattern: alternating (0); delayed (1); at most single

replacement for one position (2); sequential addition of postcanines, no

replacement (3). [*L7, H81, LCS89, B7*, LO119]

*Postcranial skeleton*

120. Vertebral centra: amphicoelous (0); platycoelous (1). [R108, H101, B51, BO78,

MO61]

121. Axis centrum: cylindrical (0) or depressed (1). [R98]

122. Dens: absent or vestigial (0) or strongly developed (1) [R99]

123. Posterior thoracic vertebrate (or middle of the dorsal vertebrate): neural spines

slightly inclined or nearly vertical (0) or strongly inclined (1). [*R102*, LO123]

124. Anapophysis: absent (0); present (1). [LO124]

125. Expanded costal plates on dorsal ribs: absent (0); present (1). [H82]

126. Lumbar costal plates with ridge overlapping preceding rib: absent (0); present (1).

[H83]

127. #Acromion process: absent (0); weak to moderate (1); strongly developed and

close to level of glenoid (2). [R115, H85]

128. Scapular constriction below the acromion process: absent (0); present (1). [H86]

129. Scapular elongation between the acromion and glenoid: absent (0); present (1).

[H87, *B41, BO68*, *MO51*, LO129]

130. Procoracoid in glenoid: present (0); barely present or absent (1). [R116, H88,

B42, BO 71, MO52]

131. Procoracoid contact with scapula: greater than coracoid contact (0); equal to or

less than coracoid contact (1). [H89, B43, BO72, MO53]

132. Humeral ectepicondylar foramen: present (0); absent (1). [R124, H90, B44,

BO73, MO54]

133. Ulnar olecranon process: unossified or poorly ossified (0); well ossified (1). [*R128,*

*H91, B45, MO55*, LO133]

134. Manual digit III phalanx number: four (0); three (1). [H92]

135. Manual digit IV phalanx number: four (0); three (1). [H93]

136. Dorsal profile of ilium: strongly convex (0); flat to concave (1). [R130, H96, B48,

BO75]

137. Length of anterior process of ilium anterior to acetabulum (relative to diameter

of acetabulum): less than 1.5 (0); greater than 1.5 (1). [H94, B46, BO74,

MO56]

138. Lateral surface of iliac blade: concave or nearly flat (0); convex (1); a longitudinal

ridge divides it into dorsal and ventral moieties (1). [*R131*, LO138]

139. Posterior iliac spine: robust and extends beyond acetabulum (0); reduced to small

nub that lies entirely anterior to acetabulum (1). [R132, *R133*, LO139]

140. Cotyloid (acetabular) notch: lies between the ischial and iliac part of the

acetabulum, mainly on ilium (0); between acetabular facet and pubic process of the

ischium, on ischium (1). [*R134*, LO140]

141. The diameter of the obturator foramen greater than that of the acetabulum: absent

(0); present (1). [*R139*, LO141]

142. Femoral head: rounded and predominately in plane of shaft (0); subspherical and

inflected dorsally (1). [R141]

143. Greater trochanter separated from femoral head by distinct notch: absent (0);

present (1). [R143, H98, B49, BO76, MO59]

144. Lesser trochanter position: on ventromedial surface of femoral shaft (0); on

medial surface of femoral shaft (1). [R144, H100, B50, BO77, MO60]

145. Lesser trochanter location near the level of the femoral head: absent (0); present

(1). [BO80, MO63]

**Literature Cited**

1. Liu J, Olsen P. The Phylogenetic Relationships of Eucynodontia (Amniota: Synapsida). J Mamm Evol. 2010 Sep;17(3):151–76.

2. Soares MB, Martinelli AG, Oliveira TVD. A new prozostrodontian cynodont (Therapsida) from the Late Triassic Riograndia Assemblage Zone (Santa Maria Supersequence) of Southern Brazil. An Acad Bras Ciênc. 2014 Dec;86(4):1673–91.

3. Martinelli AG, Soares MB, Schwanke C. Two New Cynodonts (Therapsida) from the Middle-Early Late Triassic of Brazil and Comments on South American Probainognathians. Viriot L, editor. PLOS ONE. 2016 Oct 5;11(10):e0162945.

4. Martinelli AG, Eltink E, Da-Rosa ÁAS, Langer MC. A new cynodont from the Santa Maria formation, south Brazil, improves Late Triassic probainognathian diversity. Angielczyk K, editor. Pap Palaeontol. 2017 Aug;3(3):401–23.

5. Rodrigues PG, Ruf I, Schultz CL. Digital reconstruction of the otic region and inner ear of the non-mammalian cynodont *Brasilitherium riograndensis* (Late Triassic, Brazil) and its relevance to the evolution of the mammalian ear. J Mamm Evol. 2013 Dec;20(4):291–307.

6. Timothy Rowe. Definition, Diagnosis, and Origin of Mammalia. J Vertebr Paleontol. 1988;8(3):241–64.

7. Luo Z-X. Sister-group relationships of mammals and transformations of diagnostic mammalian characters. In: Fraser NC, Sues H-D, editors. In the Shadow of the Dinosaurs: Early Mesozoic Tetrapods. New York, NY: Cambridge University Press; 1994. p. 98–128.

8. Luo Z-X, Crompton AW, Sun A-L. A New mammaliaform from the Early Jurassic and evolution of mammalian characteristics. Science. 2001 May 25;292(5521):1535–40.

9. Bonaparte JF, Martinelli AG, Schultz CL. New information on Brasilodon and Brasilitherium (Cynodontia, Probainognathia) from the late Triassic of southern Brazil. Rev Bras Paleontol. 2005;8:25–46.

10. Abdala F. Redescription of *Platycraniellus elegans* (Therapsida, Cynodontia) from the Lower Triassic of South Africa, and the cladistic relationships of eutheriodonts. Palaeontology. 2007 May;50(3):591–618.

11. Ruta M, Botha-Brink J, Mitchell SA, Benton MJ. The radiation of cynodonts and the ground plan of mammalian morphological diversity. Proc R Soc B Biol Sci. 2013 Aug 28;280(1769):20131865–20131865.

12. Sereno PC. Logical basis for morphological characters in phylogenetics. Cladistics. 2007 Sep 4;0(0):070907095847001-???

13. Bonaparte JF, Barberena MC. On two advanced carnivorous cynodonts from the Late Triassic of southern Brazil. Bull Mus Comp Zool. 2001;156:37–48.

14. Ruf I, Maier W, Rodrigues PG, Schultz CL. Nasal anatomy of the non-mammaliaform cynodont *Brasilitherium riograndensis* (Eucynodontia, Therapsida) reveals new insight into mammalian evolution: nasal anatomy of *Brasilitherium*. Anat Rec. 2014 Nov;297(11):2018–30.

15. Bonaparte JF, Martinelli AG, Schultz CL, Rubert R. The sister group of mammals: small cynodonts from the Late Triassic of southern Brazil. Rev Bras Paleontol. 2003;5:5–27.

16. John R. Wible. Origin of Mammalia: The Craniodental Evidence Reexamined. J Vertebr Paleontol. 1991;11(1):1–28.

17. Wible JR, Hopson JA. Basicranial evidence for early mammal phylogeny. In: Szalay FS, Novacek MJ, McKenna MC, editors. Mammal Phylogeny: Mesozoic Differenctiation, Multituberculates, Monotremes, Early Therians, and Marsupials. New York, NY: Springer-Verlag; 1993. p. 42–62.

18. Lucas SG, Luo Z-X. *Adelobasileus* from the Upper Triassic of West Texas: the oldest mammal. J Vertebr Paleontol. 1993;13(3):309–34.

19. Luo Z-X, Crompton AW. Transformation of the quadrate (incus) through the transition from non-mammalian cynodonts to mammals. J Vertebr Paleontol. 1994 Jan;14(3):341–74.

20. Martinez RN, May CL, Forster CA. A new carnivorous cynodont from the Ischigualasto Formation (Late Triassic, Argentina), with comments on eucynodont phylogeny. J Vertebr Paleontol. 1996;16(2):271–84.

21. Hopson JA, Kitching J. Probainognathian cynodont from South Africa. Bull Am Mus Comp Zool. 2001;156:5–35.

22. Sidor CA, Smith RMH. A new galesaurid (Therapsida: Cynodontia) from the Lower Triassic of South Africa. Palaeontology. 2004 May;47(3):535–56.

23. Martinelli AG, Bonaparte JF, Schultz CL, Rubert R. A new tritheledontid (Therapsida, Eucynodontia) from the Late Triassic of Rio Grande do Sul (Brazil) and its phylogenetic relationships among carnivorous non-mammalian eucynodonts. Ameghiniana. 2005;42:191–208.

24. Sidor CA, Hancox PJ. *Elliotherium kersteni*, a new tritheledontid from the Lower Elliot Foramtion (Upper Triassic) of South Africa. J Paleontol. 2006;80(2):10.
