# Supplementary material for: First record of a basal mammaliamorph from the early Late Triassic Ischigualasto Formation of Argentina
Source: PLoS One. 2019 Aug 7;14(8):e0218791. doi: 10.1371/journal.pone.0218791 (PMC6685608; doi:10.1371/journal.pone.0218791)
Supplement: S2 Appendix — (DOCX) [file pone.0218791.s002.docx]

S2 Appendix. Character matrix.

Distribution of the character-states for the characters listed in Appendix 1.A I among 34 taxa considered in this analysis. A=0&1, B=1&2, a=0/1, b=1/2. ?=unknown,

dash=inapplicable

*Procynosuchus delaharpeae* 0000000100 0000000000 0000000000 0000000000 0000000000 0000000000 0001A00000 0000000000 0000000100 000000010 010000000- -00-000000 0000000-00 000000000 00000

*Galesaurus* *planiceps* 0100000000 0000000010 0000100001 1000000000 0000000000 0000000000 0000000000 0000000100 0010000000 0001100100 000010000- -00-100000 0001100??? ?00??0??00 00000

*Thrinaxodon liorhinus*  01A0000000 0000000000 0000000000 1110000000 0000000000 0000000000 0000000000 0010010100 0010000011 0001100000 010000000- -00-100000 0001100-00 000000000 ?0000

*Platycraniellus elegans* 0110000000 0000000011 0000000001 0110000000 ??00000000 ?000000000 0000000000 101??10100 0?1010?0?? 0001?0?100 000000000- -0?-0?000? ?????????? ?????????? ?????

*Cynognathus crateronotus*  0000000000 0000001021 0021100000 1110000101 1100010100 ?000000000 0000100000 1110010110 1120101112 0001101000 100010000- -00-100100 0000111000 0001100000 00000

*Diademodon tetragonus*  A000000000 0000001022 0121110001 1110000001 1100010100 000000A000 0000000000 0010010111 1120101112 1131101000 1200100010 0100?00010 0001111000 0001100000 00000

*Trirachodon berryi* 11100100A0 0000001121 0111110100 1110100201 1100010100 0000021000 0000000011 0111020110 1110101112 1131101000 1211100020 110100101? ??01111000 000????000 00000

*Sinognathus gracilis* 1020?10010 0000??1101 0011110?1? 1?1010?001 1000010100 ???????000 00?000?011 0111020110 11101011?? 1131200000 02?0?00?20 1100?0201? ?????????? ?????????? ?????

*Langbergia modisei* 0010000000 0000000121 0111110100 1110100001 10000101?0 ?000?21??0 000000001? 0?11????10 1110101112 1131101000 1200100020 110100101? ????1????? ?????????? ?????

*Pascualgnathus polanskii*  1020?10010 0000001122 012111011? 1110100001 ??000101?0 ???0?2?000 00???0?0?? ????????A0 ?1201011?? 1132100000 02bb000-11 0100-12010 ???1101??0 000??10000 00000

*Luangwa drysdalli* ??00?1000? 0000010121 001111???? 1110?00001 ???0010100 0000?2??00 000???0??? ????????10 11201011?? 1131101000 12bb000-20 2100-12010 ???1101100 00???11000 00000

*Massetognathus pascuali*  0111110010 0000001101 0111110112 1110200001 0000010100 0000121100 0000000011 0111020110 11B01011?? 1131102111 0211000-22 2100-12010 0000101101 000??11000 00000

*Exaeretodon argentinus*  0011111010 0000111121 0121110112 11101A0001 0?00010100 0100021100 00000000?1 01????0110 11211011?? 1132100101 021b000-12 0100-12210 0000001101 0011111000 00000

*Scalenodon angustifrons* ??10?1?000 0000??1101 012111???? ?1101?0??1 ??00010100 ?000021100 0000?000?? 0?????0?a0 ?12??011?? 1131101000 12bb000-20 2100-1201? ?????????? ?????????? ?????

*“Scalenodon” hirschoni* ???0010??? ??001?11?? ???1110112 11101?0?0? 0??00????? ???????1?0 00?0?????1 ??1?0?0?a? ?1???011?? 1132210010 02bb000-22 2100-1201? ?????????? ?????????? ?????

*Chiniquodon theotonicus* 1110101010 0000101011 000001011B 1111211001 1000010100 0000010000 00000000?1 ?01???1110 1120101112 0001100000 000010000- -00-100000 ??00001101 0001110000 ?0000

*Lumkuia fuzzi* ??10001010 00000?0000 0000010?12 0110100101 0100010010 ?000000100 0000000000 0010000110 1120100012 0001100000 000010000- -00-10000? ????001??? ?????????0 ?????

*Ectenion lunensis* 001??00210 0000200000 0000000?1a 1110000201 1100010100 ?100010100 0000000011 0011021110 11001011?? 0001100000 1a0010000- -00-?00000 ???1??110? ?0?????0?? ?????

*Probainognathus jenseni* 0110100210 0000100101 1000010112 1111101001 1100010000 0000110000 0000000011 0011021110 2100101112 0001100000 000000000- -00-101100 00?000110? ??0??11000 ?0000

*Prozostrodon brasiliensis*  21?010?2?? ??0121?1?? ???????112 ?1111?1??? ?????????? ?????????? ?????????? ?????????? ?0301111?? 0010000000 111000000- -00-001000 ???000???? ?0???11111 10000

*Therioherpeton cargnini* ?????0111? 01122?2100 ??0??????2 ?11111???? ?????????? ?????????? ?????????? ?????????? ?????????? 00???????? ?0?000?00- -00-?00000 ??1?00???? ?0???11111 10011

*Riograndia guaibaensis* 2013101211 011221?10? 1000001112 0111111011 0001020000 ?000010200 0010001121 102?13???0 00301111?? 0012110011 00b000000- -00-10100? ?????????? ?????????? ?????

*Pachygenelus monus* 2013101211 0112212100 1000000112 0111211011 0001020000 1000010200 00101??121 1022131320 2030111112 0012210010 001000010- -00-002001 ???0001111 101??11111 11111

*Pseudotherium argentinus* ?010?00111 01011??00? ???0?00?12 01111112?0 000103?011 1000?21211 ?11??110?? ????????00 0????????? ?01???00?? 000001?10- ?00-??10?? ?????????? ?????????? ?????

*Botucaraitherium belarminoi* ?????0???? ?????????? ?????????? ?????????? ?????????? ?????????? ?????????? ?????????? ?0???11??? ?01??????0 01?001110- -01-00??0? ?????????? ?????????? ?????

*Brasilodon quardangularis* a000?01211 0112212000 1000?00?12 0111111211 0001021001 ?0?112220? 0111101121 1022131320 ?0301111?? 0011100001 011001110- -01-001aa? ?????0111? ?11??????? ?????

*Brasilitherium riograndensis* 0000?01211 0112212000 1000000?12 0111110211 0001021011 ?01112220? ?111?01121 1022131320 10301111?? 0010110001 011001110- -01-001a1? ?????????? ?11??????? ?1111

*Tritylodon longaevus* 102-111111 0112211102 0011110112 1110211211 0000110110 1101021211 1101101031 1022031202 00311111?? 1132210-22 -222-21-2- 1100-03221 ??????210? ?????1???? ?1111

*Oligokyphus major* b??-1111?1 0112???102 010110?1?2 ?1??21???? ????110?10 110102?211 1101100031 0022031202 00311111?? 1132110-22 -222-21-2- 1100-03221 11100?2101 111??11211 11111

*Bienotherium yunnanense* 102-111111 01122111?2 01?1110112 1110211211 0000110110 110?02?011 11?110?031 ??22131?02 00311111?? 1132110-22 -222-21-2- 1100-03221 ??????210? ?11??????1 11111

*Kayentatherium wellesi*  102-11111? 0112211102 0111111112 0110211211 0000110110 1101021211 11?110?031 1022131202 0031111112 1132110-22 -222-21-2- 1100-0322? 1110002101 1?1111121? ?1111

*Adelobasileus cromptoni* ???????011 11?2?????? ?????????? 0??????211 0001021011 ?211122100 00101110?? ?????????? ?????????? ?????????? ?????????? ?????????? ?????????? ?????????? ?????

*Sinoconodon rigneyi* 0002?01011 1112212000 1000?01?12 1111211211 0011031011 ?211122210 11101010?? ??????0?30 20301112?? 2001000001 001001110- -00-10101? ?????????? ?????????? ?????

*Morganucodon oehleri* 0?02?01011 1112212000 2?00001111 1111211211 0011032011 1211122201 1111111121 1022132430 2030111212 2221000001 011001110- -01-001011 1111001111 111??11211 11111
