# Supplementary material for: First record of a basal mammaliamorph from the early Late Triassic Ischigualasto Formation of Argentina
Source: PLoS One. 2019 Aug 7;14(8):e0218791. doi: 10.1371/journal.pone.0218791 (PMC6685608; doi:10.1371/journal.pone.0218791)
Supplement: S3 Appendix — Completeness of taxa analyzed. (DOCX) [file pone.0218791.s003.docx]

S3 Appendix. Completeness of taxa analyzed.

1. *Morganucodon oehleri* missing 7/145 = 95 % complete
2. *Sinoconodon rigneyi* missing 46/145 = 68 % complete
3. *Adelobasileus cromptoni* missing 111/145 = 23% complete
4. *Kayentatherium wellesi* missing 13/145 = 91% complete
5. *Bienotherium yunnanense* missing 32/145 = 78% complete
6. *Oligokyphus major* missing 33/145 = 77% complete
7. *Tritylodon longaevus* missing 25/145 = 83% complete
8. *Brasilitherium riograndensis* missing 31/145 = 79% complete
9. *Brasilodon quardangularis* missing 32/145 = 78% complete
10. *Botucaraitherium belarminoi* missing 125/145 = 14% complete
11. *Pseudotherium argentinus* missing 75/145 = 48% complete
12. *Pachygenelus monus*  missing 10/145 = 93% complete
13. *Riograndia guaibaensis* missing 38/145 = 74% complete
14. *Therioherpeton cargnini* missing 94/145 = 35% complete
15. *Prozostrodon brasiliensis* missing 78/145 = 46 % complete
16. *Probainognathus jenseni* missing 10/145 = 93% complete
17. *Ecteninion lunensis* missing 29/145 = 80% complete
18. *Lumkuia fuzzi* missing 30/145 = 79 % complete
19. *Chiniquodon theotonicus* missing 11/145 = 92% complete
20. *“Scalenodon” hirschoni* missing 78/145 = 46% complete
21. *Scalenodon angustifrons* missing 59/145 = 59% complete
22. *Exaeretodon argentinus* missing 10/145 = 93% complete
23. *Massetognathus pascuali* missing 6/145 = 96% complete
24. *Luangwa drysdalli* missing 39/145 = 73% complete
25. *Pascualgnathus polanskii* missing 34/145 = 77% complete
26. *Langbergia modisei* missing 36/145 = 75 % complete
27. *Sinognathus gracilis* missing 48/145 = 67 % complete
28. *Trirachodon berryi* missing 7/145 = 95% complete
29. *Diademodon tetragonus* missing 1/145 = 99% complete
30. *Cynognathus crateronotus* missing 4/145 = 97% complete
31. *Platycraniellus elegans* missing 40/145 = 72% complete
32. *Thrinaxodon liorhinus* missing 4/145 = 97% complete
33. *Galesaurus* *planiceps* missing 12/145 = 92% complete
34. *Procynosuchus delaharpeae* missing 4/145 = 97% complete

Average = 75% complete
