# Supplementary material for: First record of a basal mammaliamorph from the early Late Triassic Ischigualasto Formation of Argentina
Source: PLoS One. 2019 Aug 7;14(8):e0218791. doi: 10.1371/journal.pone.0218791 (PMC6685608; doi:10.1371/journal.pone.0218791)
Supplement: S4 Appendix — (DOCX) [file pone.0218791.s004.docx]

**Appendix S3**

P A U P *

Version 4.0b10 for 32-bit Microsoft Windows

Thu Jul 06 13:05:28 2017

-----------------------------NOTICE-----------------------------

This is a beta-test version. Please report any crashes,

apparent calculation errors, or other anomalous results.

There are no restrictions on publication of results obtained

with this version, but you should check the WWW site

frequently for bug announcements and/or updated versions.

See the README file on the distribution media for details.

----------------------------------------------------------------

Heuristic search settings:

Optimality criterion = parsimony

Character-status summary:

Of 145 total characters:

All characters are of type 'unord'

All characters have equal weight

All characters are parsimony-informative

Gaps are treated as "missing"

Multistate taxa interpreted as uncertainty

Starting tree(s) obtained via stepwise addition

Addition sequence: random

Number of replicates = 1000

Starting seed = 1080186551

Number of trees held at each step during stepwise addition = 1

Branch-swapping algorithm: tree-bisection-reconnection (TBR)

Steepest descent option not in effect

Initial 'MaxTrees' setting = 100

Branches collapsed (creating polytomies) if maximum branch length is zero

'MulTrees' option in effect

Topological constraints not enforced

Trees are unrooted

Heuristic search completed

Total number of rearrangements tried = 37498754

Score of best tree(s) found = 444

Number of trees retained = 8

Time used = 9.36 sec

Tree-island profile:

First Last First Times

Island Size tree tree Score replicate hit

----------------------------------------------------------------------

1 8 1 8 444 1 964

2 14 - - 447 673 1

3 6 - - 447 267 1

4 5 - - 447 58 7*

5 3 - - 447 143 1

6 2 - - 447 136 5*

7 1 - - 447 22 19*

8 0 - - 447 345 2*

Note(s):

* Multiple hits on islands of unsaved trees may in fact represent

different islands

8 trees saved to file "C:\Users\Dell Laptop\Documents\PhD\Phylogenetic

analyses\Brasilodontid analysis\Jan 11 2017\Character81edit.tre"

Strict consensus of 8 trees:

/------------------------------------------------------------ Procynosuchus

|

+------------------------------------------------------------ Galesaurus

|

| /-------------------------------------------------------- Thrinaxodon

| |

| +-------------------------------------------------------- Platycraniellus

| |

| | /---------------------------- Cynognathus

| | |

| | | /------------------------ Diademodon

| | | |

| | /-------------------+ | /---------------- Trirachodon

| | | | | |

| | | | | | /------------ Sinognathus

| | | | | /---+ |

| | | \---+ | | | /-------- Pascualgnathus

| | | | | | | |

| | | | | \---+ +-------- Luangwa

| | | | | | |

| | | | | | | /---- Massetognathus

| | | | | \---+---+

| | | \---+ | \---- Exaeretodon

| | | | |

| | | | +-------- Scalenodon

| | | | |

| | | | \-------- "Scalenodon"

\---+ | |

| | \-------------------- Langbergia

| |

| | /---------------------------------------- Chiniquodon

| /---+ |

| | | | /------------------------------------ Probainognathus

| | | /---+ |

| | | | | | /-------------------------------- Prozostrodon

| | | | \---+ |

| | | | | | /---------------------------- Therioherpeton

| | | | | | |

| | | | \---+ | /---- Riograndia

| | | | | | /-------------------+

| | | | | | | \---- Pachygenelus

| | | | \---+ |

| | | | | | /---------------- Pseudotherium

| | | | | | |

| | | | | | | /------------ Oligokyphus

| | | | \---+ /---+ |

| | | | | | | | /---- Tritylodon

| | | | | | \---+ /---+

| | | | | | | | \---- Bienotherium

\---+ \---+ | | \---+

| | \---+ \-------- Kayentatherium

| | |

| | | /---------------- Botucaraitherium

| | | |

| | | | /---- Brasilodon

| | \---+ /-------+

| | | | \---- Brasilitherium

| | | |

| | \---+ /-------- Adelobasileus

| | | |

| | \---+ /---- Sinoconodon

| | \---+

| | \---- Morganucodon

| |

| \-------------------------------------------- Ectenion

|

\---------------------------------------------------- Lumkuia

Consensus tree(s) written to treefile: C:\Users\Dell

Laptop\Documents\PhD\Phylogenetic analyses\Brasilodontid analysis\Jan 11

2017\character81edit_sc.tre

Tree description:

Unrooted tree(s) rooted using outgroup method

Optimality criterion = parsimony

Character-status summary:

Of 145 total characters:

All characters are of type 'unord'

All characters have equal weight

All characters are parsimony-informative

Gaps are treated as "missing"

Multistate taxa interpreted as uncertainty

Character-state optimization: Accelerated transformation (ACCTRAN)

Tree number 1 (rooted using default outgroup)

Tree length = 444

Consistency index (CI) = 0.4707

Homoplasy index (HI) = 0.5293

Retention index (RI) = 0.7812

Rescaled consistency index (RC) = 0.3677

/------------------------------------------------------------ Procynosuchus

|

+------------------------------------------------------------ Galesaurus

|

| /----------------------------------------------------- Thrinaxodon

| |

| | /---------------------------------- Cynognathus

| | |

| | | /------------------------------ Diademodon

| | | |

| | /---------44 | /----------------------- Trirachodon

| | | | | |

| | | | | | /------------------- Sinognathus

| | | | | /-41 |

| | | \--43 | | | /--------------- Pascualgnathus

| | | | | \--40 |

| | | | | | | /---- Luangwa

| | | | | \--39 /-----35

| | | | | | | \---- Scalenodon

| | | | | | |

| | | \--42 \--38 /---- Massetognathus

| | | | | /--36

| | | | | | \---- Exaeretodon

66 | | | \-37

| | | | \-------- "Scalenodon"

| | | |

| /-64 | \-------------------------- Langbergia

| | | |

| | | | /-------------------------------------- Chiniquodon

| | | /--62 |

| | | | | | /---------------------------------- Probainognathus

| | | | | /-60 |

| | | | | | | | /------------------------------ Prozostrodon

| | | | | | \--59 |

| | | | | | | | /-------------------------- Therioherpeton

| | | | | | | | |

| | | | | | \--58 | /---- Riograndia

| | | | | | | | /-----------------45

| | | | | | | | | \---- Pachygenelus

| | | | | | \--57 |

| | | | | | | | /--------------- Pseudotherium

| | | | | | | | |

| | | | | | | | | /----------- Oligokyphus

| | | | | | \-56 /--49 |

| | | | | | | | | | /---- Tritylodon

| | | | | | | | \--48 /--46

\--65 | | | | | | | | \---- Bienotherium

| \--63 \--61 | | \-47

| | | \--55 \-------- Kayentatherium

| | | |

| | | | /--------------- Botucaraitherium

| | | | |

| | | | | /---- Brasilodon

| | | \--54 /-----50

| | | | | \---- Brasilitherium

| | | | |

| | | \--53 /-------- Adelobasileus

| | | | |

| | | \-52 /---- Sinoconodon

| | | \--51

| | | \---- Morganucodon

| | |

| | \----------------------------------------- Ectenion

| |

| \------------------------------------------------- Lumkuia

|

\-------------------------------------------------------- Platycraniellus

Apomorphy lists:

Branch Character Steps CI Change

------------------------------------------------------------------------------

node_66 --> Procynosuchus 2 (Septomaxilla ) 1 0.143 1 ==> 0

8 (Profile of sk) 1 0.400 0 ==> 1

64 (Articulation) 1 0.250 0 ==> 1

78 (Articulation) 1 1.000 1 ==> 0

83 (#Lateral rid) 1 0.429 1 ==> 0

94 (Upper inciso) 1 0.250 1 ==> 0

95 (Lower inciso) 1 0.286 1 ==> 0

102 (Upper postc) 1 0.286 0 ==> 1

124 (Anapophysis) 1 0.200 1 ==> 0

node_66 --> Galesaurus 19 (Infraorbital) 1 0.286 0 --> 1

25 (Posterior ex) 1 0.250 1 ==> 0

30 (Ectopterygoi) 1 0.286 0 --> 1

31 (Interpterygo) 1 0.167 0 --> 1

105 (Posterior p) 1 0.250 0 ==> 1

115 (Lingual cin) 1 0.167 0 --> 1

125 (Expanded co) 1 0.250 0 --> 1

node_66 --> node_65 3 (#Snout in rel) 1 0.250 0 ==> 1

32 (Secondary pa) 1 1.000 0 ==> 1

33 (Secondary pa) 1 1.000 0 ==> 1

73 (#Lateral mar) 1 1.000 0 ==> 1

76 (#Lateral not) 1 0.600 0 ==> 1

85 (Position of ) 1 0.500 0 --> 1

89 (Reflected la) 1 1.000 0 --> 1

90 (#Reflected l) 1 1.000 0 --> 1

node_65 --> node_64 31 (Interpterygo) 1 0.167 0 --> 1

98 (Distinct upp) 1 0.500 1 ==> 0

115 (Lingual cin) 1 0.167 0 --> 1

node_64 --> Thrinaxodon 85 (Position of ) 1 0.500 1 --> 0

102 (Upper postc) 1 0.286 0 ==> 1

125 (Expanded co) 1 0.250 0 --> 1

node_64 --> node_63 2 (Septomaxilla ) 1 0.143 1 --> 0

9 (Parietal fora) 1 0.250 0 --> 1

26 (The notch se) 1 0.200 0 --> 1

29 (Vomer) 1 0.333 0 --> 1

40 (Quadrate ram) 1 0.500 0 ==> 1

42 (Frontal-epip) 1 0.200 0 ==> 1

46 (#Basisphenoi) 1 0.600 0 ==> 1

79 (Craniomandib) 1 0.750 0 ==> 1

82 (Dentary symp) 1 0.500 0 ==> 1

83 (#Lateral rid) 1 0.429 1 ==> 2

90 (#Reflected l) 1 1.000 1 --> 2

105 (Posterior p) 1 0.250 0 ==> 1

127 (#Acromion p) 1 1.000 0 ==> 1

134 (Manual digi) 1 1.000 0 --> 1

135 (Manual digi) 1 1.000 0 --> 1

node_63 --> node_62 20 (#Zygomatic a) 1 0.286 0 --> 1

41 (Quadrate art) 1 0.333 0 ==> 1

48 (Internal car) 1 0.333 0 ==> 1

87 (Splenial) 1 1.000 0 ==> 1

88 (#Postdentary) 1 1.000 0 ==> 1

101 (Canine serr) 1 0.200 0 --> 1

node_62 --> node_44 3 (#Snout in rel) 1 0.250 1 --> 0

9 (Parietal fora) 1 0.250 1 --> 0

17 (Zygomatic ar) 1 0.250 2 ==> 0

19 (Infraorbital) 1 0.286 0 ==> 2

23 (The width of) 1 0.333 0 ==> 2

24 (Squamosal gr) 1 0.500 0 ==> 1

25 (Posterior ex) 1 0.250 1 ==> 0

29 (Vomer) 1 0.333 1 --> 0

72 (Shape of the) 1 0.500 0 --> 1

97 (Incisor cutt) 1 0.500 0 ==> 1

125 (Expanded co) 1 0.250 0 --> 1

126 (Lumbar cost) 1 0.500 0 ==> 1

node_44 --> Cynognathus 26 (The notch se) 1 0.200 1 --> 0

38 (Middle of pt) 1 0.400 0 ==> 1

65 (Paroccipital) 1 0.333 0 ==> 1

71 (Size of the ) 1 0.250 0 ==> 1

118 (Upper tooth) 1 0.500 0 ==> 1

124 (Anapophysis) 1 0.200 1 ==> 0

node_44 --> node_43 22 (The posterov) 1 0.200 0 ==> 1

57 (#Maxillary a) 1 0.667 0 --> 1

91 (Mandibular m) 1 0.667 0 ==> 1

92 (Postcanine o) 1 0.667 0 ==> 1

93 (Relationship) 1 0.600 0 ==> 3

102 (Upper postc) 1 0.286 0 ==> 2

109 (Number of u) 1 0.400 0 --> 1

112 (Arrangement) 1 0.500 0 ==> 1

115 (Lingual cin) 1 0.167 1 --> 0

119 (Postcanine ) 1 0.667 0 ==> 1

node_43 --> Diademodon 20 (#Zygomatic a) 1 0.286 1 ==> 2

30 (Ectopterygoi) 1 0.286 0 ==> 1

72 (Shape of the) 1 0.500 1 --> 0

80 (Craniomandib) 1 1.000 0 ==> 1

111 (Central cus) 1 0.500 1 ==> 0

node_43 --> node_42 3 (#Snout in rel) 1 0.250 0 --> 1

18 (The anterove) 1 0.250 0 ==> 1

23 (The width of) 1 0.333 2 ==> 1

28 (Vomer exposu) 1 0.500 0 ==> 1

35 (#The posteri) 1 0.250 0 ==> 1

42 (Frontal-epip) 1 0.200 1 --> 0

56 (Route of the) 1 0.667 0 ==> 2

69 (#Rotation of) 1 0.750 0 ==> 1

70 (Curvature of) 1 0.500 0 --> 1

74 (#Medial marg) 1 0.667 0 ==> 1

76 (#Lateral not) 1 0.600 1 --> 2

83 (#Lateral rid) 1 0.429 2 ==> 1

109 (Number of u) 1 0.400 1 --> 2

114 (Number of l) 1 0.500 0 --> 1

117 (Axis of pos) 1 0.500 0 ==> 1

136 (Dorsal prof) 1 0.500 0 --> 1

node_42 --> node_41 1 (#Premaxillary) 1 0.333 0 ==> 1

6 (Maxillary pla) 1 0.500 0 ==> 1

9 (Parietal fora) 1 0.250 0 --> 1

103 (#Anteriormo) 1 0.500 0 ==> 1

104 (#Posteriorm) 1 0.667 0 --> 1

node_41 --> Trirachodon 2 (Septomaxilla ) 1 0.143 0 ==> 1

38 (Middle of pt) 1 0.400 0 ==> 2

42 (Frontal-epip) 1 0.200 0 --> 1

node_41 --> node_40 3 (#Snout in rel) 1 0.250 1 --> 2

29 (Vomer) 1 0.333 0 ==> 1

30 (Ectopterygoi) 1 0.286 0 --> 2

97 (Incisor cutt) 1 0.500 1 ==> 0

101 (Canine serr) 1 0.200 1 ==> 0

105 (Posterior p) 1 0.250 1 --> 0

114 (Number of l) 1 0.500 1 --> 0

117 (Axis of pos) 1 0.500 1 ==> 2

126 (Lumbar cost) 1 0.500 1 --> 0

128 (Scapular co) 1 0.500 0 --> 1

node_40 --> Sinognathus 19 (Infraorbital) 1 0.286 2 ==> 0

22 (The posterov) 1 0.200 1 ==> 0

95 (Lower inciso) 1 0.286 1 ==> 2

104 (#Posteriorm) 1 0.667 1 --> 0

node_40 --> node_39 23 (The width of) 1 0.333 1 --> 2

41 (Quadrate art) 1 0.333 1 --> 0

83 (#Lateral rid) 1 0.429 1 ==> 2

94 (Upper inciso) 1 0.250 1 --> 2

111 (Central cus) 1 0.500 1 --> 0

116 (Lower poste) 1 1.000 0 ==> 1

node_39 --> Pascualgnathus 20 (#Zygomatic a) 1 0.286 1 ==> 2

109 (Number of u) 1 0.400 2 ==> 1

110 (Position of) 1 1.000 0 ==> 1

node_39 --> node_38 1 (#Premaxillary) 1 0.333 1 --> 0

3 (#Snout in rel) 1 0.250 2 --> 1

16 (Sphenopalati) 1 0.333 0 --> 1

58 (Pterygoparoc) 1 0.333 0 ==> 1

111 (Central cus) 1 0.500 0 --> 2

137 (Length of a) 1 0.500 0 ==> 1

node_38 --> node_35 9 (Parietal fora) 1 0.250 1 ==> 0

94 (Upper inciso) 1 0.250 2 --> 1

97 (Incisor cutt) 1 0.500 0 ==> 1

101 (Canine serr) 1 0.200 0 ==> 1

node_35 --> Luangwa 3 (#Snout in rel) 1 0.250 1 ==> 0

17 (Zygomatic ar) 1 0.250 0 ==> 2

22 (The posterov) 1 0.200 1 ==> 0

23 (The width of) 1 0.333 2 --> 1

node_35 --> Scalenodon 19 (Infraorbital) 1 0.286 2 ==> 0

node_38 --> node_37 15 (#Palatine) 1 0.333 0 --> 1

99 (Upper canine) 1 0.500 0 --> 1

110 (Position of) 1 1.000 0 ==> 2

124 (Anapophysis) 1 0.200 1 --> 0

130 (Procoracoid) 1 0.500 0 --> 1

node_37 --> node_36 4 (Paracanine fo) 1 1.000 0 ==> 1

5 (Premaxilla fo) 1 0.500 0 ==> 1

98 (Distinct upp) 1 0.500 0 ==> 1

100 (Lower canin) 1 0.500 0 ==> 1

node_36 --> Massetognathus 2 (Septomaxilla ) 1 0.143 0 ==> 1

15 (#Palatine) 1 0.333 1 --> 0

16 (Sphenopalati) 1 0.333 1 --> 0

19 (Infraorbital) 1 0.286 2 ==> 0

23 (The width of) 1 0.333 2 --> 1

35 (#The posteri) 1 0.250 1 ==> 2

55 (Foramen and ) 1 0.333 0 ==> 1

94 (Upper inciso) 1 0.250 2 --> 1

97 (Incisor cutt) 1 0.500 0 ==> 2

node_36 --> Exaeretodon 7 (Maxilla) 1 0.200 0 ==> 1

52 (#The trigemi) 1 0.500 0 ==> 1

84 (Angle of the) 1 0.500 0 ==> 1

99 (Upper canine) 1 0.500 1 --> 0

109 (Number of u) 1 0.400 2 ==> 1

111 (Central cus) 1 0.500 2 ==> 0

118 (Upper tooth) 1 0.500 0 ==> 2

125 (Expanded co) 1 0.250 1 ==> 0

133 (Ulna olecra) 1 0.500 0 ==> 1

node_37 --> "Scalenodon" 95 (Lower inciso) 1 0.286 1 ==> 2

node_42 --> Langbergia 17 (Zygomatic ar) 1 0.250 0 ==> 2

node_62 --> node_61 5 (Premaxilla fo) 1 0.500 0 --> 1

8 (Profile of sk) 1 0.400 0 --> 2

15 (#Palatine) 1 0.333 0 --> 1

28 (Vomer exposu) 1 0.500 0 --> 1

56 (Route of the) 1 0.667 0 ==> 1

69 (#Rotation of) 1 0.750 0 ==> 1

70 (Curvature of) 1 0.500 0 ==> 1

74 (#Medial marg) 1 0.667 0 ==> 1

76 (#Lateral not) 1 0.600 1 ==> 2

77 (Articulation) 1 0.667 0 ==> 1

83 (#Lateral rid) 1 0.429 2 --> 0

128 (Scapular co) 1 0.500 0 --> 1

130 (Procoracoid) 1 0.500 0 --> 1

136 (Dorsal prof) 1 0.500 0 --> 1

141 (The diamete) 1 1.000 0 --> 1

node_61 --> node_60 2 (Septomaxilla ) 1 0.143 0 --> 1

7 (Maxilla) 1 0.200 0 --> 1

30 (Ectopterygoi) 1 0.286 0 ==> 2

34 (Posterior ex) 1 0.500 0 ==> 1

35 (#The posteri) 1 0.250 0 --> 1

36 (Osseous pala) 1 0.500 0 --> 1

37 (Contribution) 1 0.500 0 ==> 1

42 (Frontal-epip) 1 0.200 1 --> 0

101 (Canine serr) 1 0.200 1 --> 0

124 (Anapophysis) 1 0.200 1 ==> 0

node_60 --> Chiniquodon 1 (#Premaxillary) 1 0.333 0 ==> 1

8 (Profile of sk) 1 0.400 2 --> 0

17 (Zygomatic ar) 1 0.250 2 ==> 0

19 (Infraorbital) 1 0.286 0 ==> 1

35 (#The posteri) 1 0.250 1 --> 2

83 (#Lateral rid) 1 0.429 0 --> 2

node_60 --> node_59 18 (The anterove) 1 0.250 0 ==> 1

21 (Posterior ex) 1 0.667 0 ==> 1

48 (Internal car) 1 0.333 1 ==> 0

81 (Squamosal ar) 1 0.667 0 --> 1

105 (Posterior p) 1 0.250 1 ==> 0

117 (Axis of pos) 1 0.500 0 ==> 1

123 (Posterior t) 1 1.000 0 --> 1

131 (Procoracoid) 1 1.000 0 --> 1

137 (Length of a) 1 0.500 0 ==> 1

node_59 --> Probainognathus 7 (Maxilla) 1 0.200 1 --> 0

36 (Osseous pala) 1 0.500 1 --> 0

42 (Frontal-epip) 1 0.200 0 --> 1

55 (Foramen and ) 1 0.333 0 ==> 1

81 (Squamosal ar) 1 0.667 1 --> 2

118 (Upper tooth) 1 0.500 0 ==> 1

node_59 --> node_58 1 (#Premaxillary) 1 0.333 0 --> 2

10 (Interparieta) 1 1.000 0 --> 1

12 (Parietal cre) 1 1.000 0 --> 1

15 (#Palatine) 1 0.333 1 --> 2

16 (Sphenopalati) 1 0.333 0 ==> 1

17 (Zygomatic ar) 1 0.250 2 --> 1

20 (#Zygomatic a) 1 0.286 1 --> 0

26 (The notch se) 1 0.200 1 --> 0

31 (Interpterygo) 1 0.167 1 --> 0

39 (The nasophar) 1 1.000 0 --> 1

41 (Quadrate art) 1 0.333 1 --> 0

44 (The anterior) 1 0.500 0 --> 1

46 (#Basisphenoi) 1 0.600 1 --> 2

51 (Internal aud) 1 1.000 0 --> 1

58 (Pterygoparoc) 1 0.333 0 --> 2

63 (Separation o) 1 0.500 0 --> 1

65 (Paroccipital) 1 0.333 0 --> 1

67 (The relation) 1 0.500 0 --> 1

69 (#Rotation of) 1 0.750 1 --> 2

71 (Size of the ) 1 0.250 0 --> 1

73 (#Lateral mar) 1 1.000 1 --> 2

74 (#Medial marg) 1 0.667 1 --> 2

75 (Dorsal margi) 1 0.333 0 --> 1

76 (#Lateral not) 1 0.600 2 --> 3

78 (Articulation) 1 1.000 1 --> 3

79 (Craniomandib) 1 0.750 1 --> 2

82 (Dentary symp) 1 0.500 1 ==> 0

83 (#Lateral rid) 1 0.429 0 --> 3

86 (Mediolateral) 1 1.000 0 ==> 1

93 (Relationship) 1 0.600 0 ==> 1

94 (Upper inciso) 1 0.250 1 --> 0

103 (#Anteriormo) 1 0.500 0 ==> 1

106 (Upper postc) 1 0.750 0 ==> 1

107 (Lower postc) 1 0.500 0 ==> 1

108 (Buccal (ext) 1 1.000 0 ==> 1

115 (Lingual cin) 1 0.167 1 ==> 0

121 (Axis centru) 1 1.000 0 --> 1

122 (Dens) 1 1.000 0 --> 1

129 (Scapular el) 1 0.500 0 --> 1

133 (Ulna olecra) 1 0.500 0 --> 1

138 (Lateral sur) 1 1.000 0 ==> 1

139 (Posterior i) 1 1.000 0 ==> 1

140 (Cotyloid (a) 1 1.000 0 ==> 1

node_58 --> Prozostrodon 95 (Lower inciso) 1 0.286 1 ==> 0

101 (Canine serr) 1 0.200 0 ==> 1

102 (Upper postc) 1 0.286 0 ==> 1

node_58 --> node_57 2 (Septomaxilla ) 1 0.143 1 --> 0

13 (Prefrontal) 1 0.333 0 ==> 1

14 (#Postorbital) 1 1.000 0 ==> 2

94 (Upper inciso) 1 0.250 0 --> 2

96 (Incisor size) 1 0.500 0 --> 1

144 (Lesser troc) 1 1.000 0 ==> 1

145 (Lesser troc) 1 1.000 0 ==> 1

node_57 --> Therioherpeton 117 (Axis of pos) 1 0.500 1 ==> 0

node_57 --> node_56 120 (Vertebral c) 1 1.000 0 ==> 1

142 (Femur head) 1 1.000 0 ==> 1

143 (Greater tro) 1 1.000 0 ==> 1

node_56 --> node_45 4 (Paracanine fo) 1 1.000 0 ==> 3

68 (Shape of the) 1 0.333 0 ==> 1

99 (Upper canine) 1 0.500 0 ==> 1

node_45 --> Riograndia 17 (Zygomatic ar) 1 0.250 1 ==> 2

27 (Palatine) 1 0.333 0 ==> 1

65 (Paroccipital) 1 0.333 1 --> 0

100 (Lower canin) 1 0.500 0 ==> 1

115 (Lingual cin) 1 0.167 0 ==> 1

node_45 --> Pachygenelus 35 (#The posteri) 1 0.250 1 ==> 2

95 (Lower inciso) 1 0.286 1 ==> 2

106 (Upper postc) 1 0.750 1 ==> 0

107 (Lower postc) 1 0.500 1 ==> 0

117 (Axis of pos) 1 0.500 1 ==> 2

node_56 --> node_55 1 (#Premaxillary) 1 0.333 2 --> 0

18 (The anterove) 1 0.250 1 --> 0

25 (Posterior ex) 1 0.250 1 --> 0

38 (Middle of pt) 1 0.400 0 ==> 2

49 (Prootic and ) 1 0.333 0 ==> 1

50 (Promontorium) 1 0.500 0 --> 1

54 (Vascular for) 1 0.500 0 --> 1

56 (Route of the) 1 0.667 1 ==> 2

57 (#Maxillary a) 1 0.667 0 --> 1

62 (Paroccipital) 1 0.500 0 ==> 1

64 (Articulation) 1 0.250 0 --> 1

119 (Postcanine ) 1 0.667 0 --> 1

132 (Humerus ect) 1 1.000 0 ==> 1

138 (Lateral sur) 1 1.000 1 ==> 2

node_55 --> node_49 1 (#Premaxillary) 1 0.333 0 --> 1

8 (Profile of sk) 1 0.400 2 ==> 1

17 (Zygomatic ar) 1 0.250 1 --> 0

20 (#Zygomatic a) 1 0.286 0 --> 2

21 (Posterior ex) 1 0.667 1 --> 0

22 (The posterov) 1 0.200 0 --> 1

46 (#Basisphenoi) 1 0.600 2 --> 1

59 (Vertical com) 1 0.500 0 ==> 1

60 (Anterior par) 1 0.500 0 ==> 1

61 (Hyoid (stape) 1 0.500 0 --> 1

69 (#Rotation of) 1 0.750 2 --> 3

78 (Articulation) 1 1.000 3 --> 2

79 (Craniomandib) 1 0.750 2 ==> 0

84 (Angle of the) 1 0.500 0 --> 1

91 (Mandibular m) 1 0.667 0 --> 1

100 (Lower canin) 1 0.500 0 --> 2

103 (#Anteriormo) 1 0.500 1 --> 0

107 (Lower postc) 1 0.500 1 --> 2

119 (Postcanine ) 1 0.667 1 --> 2

127 (#Acromion p) 1 1.000 1 --> 2

129 (Scapular el) 1 0.500 1 --> 0

node_49 --> Pseudotherium 2 (Septomaxilla ) 1 0.143 0 ==> 1

7 (Maxilla) 1 0.200 1 ==> 0

13 (Prefrontal) 1 0.333 1 ==> 0

14 (#Postorbital) 1 1.000 2 ==> 1

15 (#Palatine) 1 0.333 2 ==> 1

40 (Quadrate ram) 1 0.500 1 ==> 0

46 (#Basisphenoi) 1 0.600 1 --> 3

54 (Vascular for) 1 0.500 1 --> 0

66 (Tabular) 1 0.333 0 ==> 1

node_49 --> node_48 3 (#Snout in rel) 1 0.250 1 --> 2

6 (Maxillary pla) 1 0.500 0 ==> 1

18 (The anterove) 1 0.250 0 --> 1

24 (Squamosal gr) 1 0.500 0 ==> 1

34 (Posterior ex) 1 0.500 1 --> 0

35 (#The posteri) 1 0.250 1 ==> 2

44 (The anterior) 1 0.500 1 --> 0

45 (Parasphenoid) 1 1.000 0 ==> 1

48 (Internal car) 1 0.333 0 --> 1

50 (Promontorium) 1 0.500 1 --> 0

52 (#The trigemi) 1 0.500 0 ==> 1

63 (Separation o) 1 0.500 1 ==> 0

80 (Craniomandib) 1 1.000 0 ==> 2

92 (Postcanine o) 1 0.667 0 ==> 1

93 (Relationship) 1 0.600 1 ==> 3

99 (Upper canine) 1 0.500 0 ==> 2

102 (Upper postc) 1 0.286 0 ==> 2

103 (#Anteriormo) 1 0.500 0 --> 2

104 (#Posteriorm) 1 0.667 0 ==> 2

106 (Upper postc) 1 0.750 1 ==> 3

109 (Number of u) 1 0.400 0 ==> 2

112 (Arrangement) 1 0.500 0 ==> 1

117 (Axis of pos) 1 0.500 1 ==> 3

118 (Upper tooth) 1 0.500 0 ==> 2

node_48 --> Oligokyphus 67 (The relation) 1 0.500 1 ==> 0

71 (Size of the ) 1 0.250 1 ==> 0

75 (Dorsal margi) 1 0.333 1 ==> 0

node_48 --> node_47 23 (The width of) 1 0.333 0 ==> 1

26 (The notch se) 1 0.200 0 ==> 1

node_47 --> node_46 31 (Interpterygo) 1 0.167 0 ==> 1

node_46 --> Tritylodon 22 (The posterov) 1 0.200 1 ==> 0

75 (Dorsal margi) 1 0.333 1 ==> 0

95 (Lower inciso) 1 0.286 1 ==> 2

node_46 --> Bienotherium 58 (Pterygoparoc) 1 0.333 2 ==> 0

node_47 --> Kayentatherium 27 (Palatine) 1 0.333 0 ==> 1

node_55 --> node_54 3 (#Snout in rel) 1 0.250 1 --> 0

47 (#Overlap of ) 1 1.000 0 --> 1

53 (Lateral trou) 1 1.000 0 --> 1

55 (Foramen and ) 1 0.333 0 --> 1

57 (#Maxillary a) 1 0.667 1 --> 2

94 (Upper inciso) 1 0.250 2 --> 1

96 (Incisor size) 1 0.500 1 --> 0

102 (Upper postc) 1 0.286 0 ==> 1

113 (Interlockin) 1 0.500 0 ==> 1

124 (Anapophysis) 1 0.200 0 --> 1

node_54 --> node_53 100 (Lower canin) 1 0.500 0 ==> 1

node_53 --> node_50 68 (Shape of the) 1 0.333 0 ==> 1

81 (Squamosal ar) 1 0.667 1 --> 0

node_50 --> Brasilodon 49 (Prootic and ) 1 0.333 1 ==> 0

node_50 --> Brasilitherium 13 (Prefrontal) 1 0.333 1 ==> 0

17 (Zygomatic ar) 1 0.250 1 ==> 2

37 (Contribution) 1 0.500 1 ==> 0

94 (Upper inciso) 1 0.250 1 ==> 0

node_53 --> node_52 4 (Paracanine fo) 1 1.000 0 --> 2

8 (Profile of sk) 1 0.400 2 ==> 0

11 (#Lateral exp) 1 1.000 0 ==> 1

25 (Posterior ex) 1 0.250 0 --> 1

27 (Palatine) 1 0.333 0 --> 1

35 (#The posteri) 1 0.250 1 --> 2

52 (#The trigemi) 1 0.500 0 ==> 2

64 (Articulation) 1 0.250 1 --> 0

66 (Tabular) 1 0.333 0 --> 1

77 (Articulation) 1 0.667 1 --> 0

78 (Articulation) 1 1.000 3 --> 4

79 (Craniomandib) 1 0.750 2 --> 3

88 (#Postdentary) 1 1.000 1 --> 2

91 (Mandibular m) 1 0.667 0 --> 2

93 (Relationship) 1 0.600 1 --> 0

95 (Lower inciso) 1 0.286 1 --> 0

106 (Upper postc) 1 0.750 1 --> 2

107 (Lower postc) 1 0.500 1 --> 2

node_52 --> Adelobasileus 58 (Pterygoparoc) 1 0.333 2 ==> 1

62 (Paroccipital) 1 0.500 1 ==> 0

node_52 --> node_51 31 (Interpterygo) 1 0.167 0 ==> 1

43 (Epipterygoid) 1 1.000 0 ==> 1

46 (#Basisphenoi) 1 0.600 2 ==> 3

61 (Hyoid (stape) 1 0.500 0 ==> 1

node_51 --> Sinoconodon 59 (Vertical com) 1 0.500 0 ==> 1

66 (Tabular) 1 0.333 1 --> 0

102 (Upper postc) 1 0.286 1 ==> 0

113 (Interlockin) 1 0.500 1 ==> 0

115 (Lingual cin) 1 0.167 0 ==> 1

node_51 --> Morganucodon 21 (Posterior ex) 1 0.667 1 ==> 2

30 (Ectopterygoi) 1 0.286 2 ==> 1

47 (#Overlap of ) 1 1.000 1 ==> 2

60 (Anterior par) 1 0.500 0 ==> 1

64 (Articulation) 1 0.250 0 --> 1

68 (Shape of the) 1 0.333 0 ==> 1

77 (Articulation) 1 0.667 0 --> 2

92 (Postcanine o) 1 0.667 0 ==> 2

93 (Relationship) 1 0.600 0 --> 2

node_61 --> Ectenion 15 (#Palatine) 1 0.333 1 --> 2

20 (#Zygomatic a) 1 0.286 1 --> 0

26 (The notch se) 1 0.200 1 --> 0

38 (Middle of pt) 1 0.400 0 ==> 2

52 (#The trigemi) 1 0.500 0 ==> 1

58 (Pterygoparoc) 1 0.333 0 ==> 1

node_63 --> Lumkuia 7 (Maxilla) 1 0.200 0 ==> 1

30 (Ectopterygoi) 1 0.286 0 ==> 2

31 (Interpterygo) 1 0.167 1 --> 0

35 (#The posteri) 1 0.250 0 ==> 1

38 (Middle of pt) 1 0.400 0 ==> 1

49 (Prootic and ) 1 0.333 0 ==> 1

58 (Pterygoparoc) 1 0.333 0 ==> 1

76 (#Lateral not) 1 0.600 1 ==> 0

node_65 --> Platycraniellus 19 (Infraorbital) 1 0.286 0 --> 1

20 (#Zygomatic a) 1 0.286 0 ==> 1

30 (Ectopterygoi) 1 0.286 0 --> 1

71 (Size of the ) 1 0.250 0 ==> 1

Tree description:

Unrooted tree(s) rooted using outgroup method

Optimality criterion = parsimony

Character-status summary:

Of 145 total characters:

All characters are of type 'unord'

All characters have equal weight

All characters are parsimony-informative

Gaps are treated as "missing"

Multistate taxa interpreted as uncertainty

Character-state optimization: Delayed transformation (DELTRAN)

Tree number 1 (rooted using default outgroup)

Tree length = 444

Consistency index (CI) = 0.4707

Homoplasy index (HI) = 0.5293

Retention index (RI) = 0.7812

Rescaled consistency index (RC) = 0.3677

/------------------------------------------------------------ Procynosuchus

|

+------------------------------------------------------------ Galesaurus

|

| /----------------------------------------------------- Thrinaxodon

| |

| | /---------------------------------- Cynognathus

| | |

| | | /------------------------------ Diademodon

| | | |

| | /---------44 | /----------------------- Trirachodon

| | | | | |

| | | | | | /------------------- Sinognathus

| | | | | /-41 |

| | | \--43 | | | /--------------- Pascualgnathus

| | | | | \--40 |

| | | | | | | /---- Luangwa

| | | | | \--39 /-----35

| | | | | | | \---- Scalenodon

| | | | | | |

| | | \--42 \--38 /---- Massetognathus

| | | | | /--36

| | | | | | \---- Exaeretodon

66 | | | \-37

| | | | \-------- "Scalenodon"

| | | |

| /-64 | \-------------------------- Langbergia

| | | |

| | | | /-------------------------------------- Chiniquodon

| | | /--62 |

| | | | | | /---------------------------------- Probainognathus

| | | | | /-60 |

| | | | | | | | /------------------------------ Prozostrodon

| | | | | | \--59 |

| | | | | | | | /-------------------------- Therioherpeton

| | | | | | | | |

| | | | | | \--58 | /---- Riograndia

| | | | | | | | /-----------------45

| | | | | | | | | \---- Pachygenelus

| | | | | | \--57 |

| | | | | | | | /--------------- Pseudotherium

| | | | | | | | |

| | | | | | | | | /----------- Oligokyphus

| | | | | | \-56 /--49 |

| | | | | | | | | | /---- Tritylodon

| | | | | | | | \--48 /--46

\--65 | | | | | | | | \---- Bienotherium

| \--63 \--61 | | \-47

| | | \--55 \-------- Kayentatherium

| | | |

| | | | /--------------- Botucaraitherium

| | | | |

| | | | | /---- Brasilodon

| | | \--54 /-----50

| | | | | \---- Brasilitherium

| | | | |

| | | \--53 /-------- Adelobasileus

| | | | |

| | | \-52 /---- Sinoconodon

| | | \--51

| | | \---- Morganucodon

| | |

| | \----------------------------------------- Ectenion

| |

| \------------------------------------------------- Lumkuia

|

\-------------------------------------------------------- Platycraniellus

Apomorphy lists:

Branch Character Steps CI Change

------------------------------------------------------------------------------

node_66 --> Procynosuchus 2 (Septomaxilla ) 1 0.143 1 ==> 0

8 (Profile of sk) 1 0.400 0 ==> 1

64 (Articulation) 1 0.250 0 ==> 1

78 (Articulation) 1 1.000 1 ==> 0

83 (#Lateral rid) 1 0.429 1 ==> 0

94 (Upper inciso) 1 0.250 1 ==> 0

95 (Lower inciso) 1 0.286 1 ==> 0

102 (Upper postc) 1 0.286 0 ==> 1

124 (Anapophysis) 1 0.200 1 ==> 0

node_66 --> Galesaurus 19 (Infraorbital) 1 0.286 0 --> 1

25 (Posterior ex) 1 0.250 1 ==> 0

30 (Ectopterygoi) 1 0.286 0 --> 1

31 (Interpterygo) 1 0.167 0 --> 1

105 (Posterior p) 1 0.250 0 ==> 1

115 (Lingual cin) 1 0.167 0 --> 1

125 (Expanded co) 1 0.250 0 --> 1

node_66 --> node_65 3 (#Snout in rel) 1 0.250 0 ==> 1

32 (Secondary pa) 1 1.000 0 ==> 1

33 (Secondary pa) 1 1.000 0 ==> 1

73 (#Lateral mar) 1 1.000 0 ==> 1

76 (#Lateral not) 1 0.600 0 ==> 1

node_65 --> node_64 89 (Reflected la) 1 1.000 0 --> 1

98 (Distinct upp) 1 0.500 1 ==> 0

115 (Lingual cin) 1 0.167 0 --> 1

node_64 --> Thrinaxodon 31 (Interpterygo) 1 0.167 0 --> 1

90 (#Reflected l) 1 1.000 0 --> 1

102 (Upper postc) 1 0.286 0 ==> 1

125 (Expanded co) 1 0.250 0 --> 1

node_64 --> node_63 40 (Quadrate ram) 1 0.500 0 ==> 1

42 (Frontal-epip) 1 0.200 0 ==> 1

46 (#Basisphenoi) 1 0.600 0 ==> 1

79 (Craniomandib) 1 0.750 0 ==> 1

82 (Dentary symp) 1 0.500 0 ==> 1

83 (#Lateral rid) 1 0.429 1 ==> 2

85 (Position of ) 1 0.500 0 --> 1

90 (#Reflected l) 1 1.000 0 --> 2

105 (Posterior p) 1 0.250 0 ==> 1

127 (#Acromion p) 1 1.000 0 ==> 1

node_63 --> node_62 31 (Interpterygo) 1 0.167 0 --> 1

41 (Quadrate art) 1 0.333 0 ==> 1

48 (Internal car) 1 0.333 0 ==> 1

87 (Splenial) 1 1.000 0 ==> 1

88 (#Postdentary) 1 1.000 0 ==> 1

134 (Manual digi) 1 1.000 0 --> 1

135 (Manual digi) 1 1.000 0 --> 1

node_62 --> node_44 2 (Septomaxilla ) 1 0.143 1 --> 0

17 (Zygomatic ar) 1 0.250 2 ==> 0

19 (Infraorbital) 1 0.286 0 ==> 2

20 (#Zygomatic a) 1 0.286 0 --> 1

23 (The width of) 1 0.333 0 ==> 2

24 (Squamosal gr) 1 0.500 0 ==> 1

25 (Posterior ex) 1 0.250 1 ==> 0

97 (Incisor cutt) 1 0.500 0 ==> 1

101 (Canine serr) 1 0.200 0 --> 1

125 (Expanded co) 1 0.250 0 --> 1

126 (Lumbar cost) 1 0.500 0 ==> 1

node_44 --> Cynognathus 3 (#Snout in rel) 1 0.250 1 --> 0

38 (Middle of pt) 1 0.400 0 ==> 1

65 (Paroccipital) 1 0.333 0 ==> 1

71 (Size of the ) 1 0.250 0 ==> 1

72 (Shape of the) 1 0.500 0 --> 1

118 (Upper tooth) 1 0.500 0 ==> 1

124 (Anapophysis) 1 0.200 1 ==> 0

node_44 --> node_43 22 (The posterov) 1 0.200 0 ==> 1

26 (The notch se) 1 0.200 0 --> 1

91 (Mandibular m) 1 0.667 0 ==> 1

92 (Postcanine o) 1 0.667 0 ==> 1

93 (Relationship) 1 0.600 0 ==> 3

102 (Upper postc) 1 0.286 0 ==> 2

112 (Arrangement) 1 0.500 0 ==> 1

119 (Postcanine ) 1 0.667 0 ==> 1

node_43 --> Diademodon 3 (#Snout in rel) 1 0.250 1 --> 0

20 (#Zygomatic a) 1 0.286 1 ==> 2

30 (Ectopterygoi) 1 0.286 0 ==> 1

80 (Craniomandib) 1 1.000 0 ==> 1

109 (Number of u) 1 0.400 0 --> 1

111 (Central cus) 1 0.500 1 ==> 0

node_43 --> node_42 18 (The anterove) 1 0.250 0 ==> 1

23 (The width of) 1 0.333 2 ==> 1

28 (Vomer exposu) 1 0.500 0 ==> 1

35 (#The posteri) 1 0.250 0 ==> 1

56 (Route of the) 1 0.667 0 ==> 2

57 (#Maxillary a) 1 0.667 0 --> 1

69 (#Rotation of) 1 0.750 0 ==> 1

74 (#Medial marg) 1 0.667 0 ==> 1

83 (#Lateral rid) 1 0.429 2 ==> 1

109 (Number of u) 1 0.400 0 --> 2

115 (Lingual cin) 1 0.167 1 --> 0

117 (Axis of pos) 1 0.500 0 ==> 1

node_42 --> node_41 1 (#Premaxillary) 1 0.333 0 ==> 1

6 (Maxillary pla) 1 0.500 0 ==> 1

70 (Curvature of) 1 0.500 0 --> 1

72 (Shape of the) 1 0.500 0 --> 1

76 (#Lateral not) 1 0.600 1 --> 2

103 (#Anteriormo) 1 0.500 0 ==> 1

node_41 --> Trirachodon 2 (Septomaxilla ) 1 0.143 0 ==> 1

38 (Middle of pt) 1 0.400 0 ==> 2

104 (#Posteriorm) 1 0.667 0 --> 1

114 (Number of l) 1 0.500 0 --> 1

node_41 --> node_40 9 (Parietal fora) 1 0.250 0 --> 1

29 (Vomer) 1 0.333 0 ==> 1

42 (Frontal-epip) 1 0.200 1 --> 0

97 (Incisor cutt) 1 0.500 1 ==> 0

101 (Canine serr) 1 0.200 1 ==> 0

117 (Axis of pos) 1 0.500 1 ==> 2

node_40 --> Sinognathus 3 (#Snout in rel) 1 0.250 1 --> 2

19 (Infraorbital) 1 0.286 2 ==> 0

22 (The posterov) 1 0.200 1 ==> 0

95 (Lower inciso) 1 0.286 1 ==> 2

node_40 --> node_39 83 (#Lateral rid) 1 0.429 1 ==> 2

104 (#Posteriorm) 1 0.667 0 --> 1

105 (Posterior p) 1 0.250 1 --> 0

116 (Lower poste) 1 1.000 0 ==> 1

126 (Lumbar cost) 1 0.500 1 --> 0

136 (Dorsal prof) 1 0.500 0 --> 1

node_39 --> Pascualgnathus 3 (#Snout in rel) 1 0.250 1 --> 2

20 (#Zygomatic a) 1 0.286 1 ==> 2

23 (The width of) 1 0.333 1 --> 2

94 (Upper inciso) 1 0.250 1 --> 2

109 (Number of u) 1 0.400 2 ==> 1

110 (Position of) 1 1.000 0 ==> 1

111 (Central cus) 1 0.500 1 --> 0

node_39 --> node_38 58 (Pterygoparoc) 1 0.333 0 ==> 1

111 (Central cus) 1 0.500 1 --> 2

128 (Scapular co) 1 0.500 0 --> 1

137 (Length of a) 1 0.500 0 ==> 1

node_38 --> node_35 9 (Parietal fora) 1 0.250 1 ==> 0

97 (Incisor cutt) 1 0.500 0 ==> 1

101 (Canine serr) 1 0.200 0 ==> 1

node_35 --> Luangwa 3 (#Snout in rel) 1 0.250 1 ==> 0

16 (Sphenopalati) 1 0.333 0 --> 1

17 (Zygomatic ar) 1 0.250 0 ==> 2

22 (The posterov) 1 0.200 1 ==> 0

node_35 --> Scalenodon 19 (Infraorbital) 1 0.286 2 ==> 0

23 (The width of) 1 0.333 1 --> 2

node_38 --> node_37 30 (Ectopterygoi) 1 0.286 0 --> 2

41 (Quadrate art) 1 0.333 1 --> 0

110 (Position of) 1 1.000 0 ==> 2

node_37 --> node_36 1 (#Premaxillary) 1 0.333 1 --> 0

4 (Paracanine fo) 1 1.000 0 ==> 1

5 (Premaxilla fo) 1 0.500 0 ==> 1

98 (Distinct upp) 1 0.500 0 ==> 1

100 (Lower canin) 1 0.500 0 ==> 1

124 (Anapophysis) 1 0.200 1 --> 0

130 (Procoracoid) 1 0.500 0 --> 1

node_36 --> Massetognathus 2 (Septomaxilla ) 1 0.143 0 ==> 1

19 (Infraorbital) 1 0.286 2 ==> 0

35 (#The posteri) 1 0.250 1 ==> 2

55 (Foramen and ) 1 0.333 0 ==> 1

97 (Incisor cutt) 1 0.500 0 ==> 2

99 (Upper canine) 1 0.500 0 --> 1

node_36 --> Exaeretodon 7 (Maxilla) 1 0.200 0 ==> 1

15 (#Palatine) 1 0.333 0 --> 1

16 (Sphenopalati) 1 0.333 0 --> 1

23 (The width of) 1 0.333 1 --> 2

52 (#The trigemi) 1 0.500 0 ==> 1

84 (Angle of the) 1 0.500 0 ==> 1

94 (Upper inciso) 1 0.250 1 --> 2

109 (Number of u) 1 0.400 2 ==> 1

111 (Central cus) 1 0.500 2 ==> 0

118 (Upper tooth) 1 0.500 0 ==> 2

125 (Expanded co) 1 0.250 1 ==> 0

133 (Ulna olecra) 1 0.500 0 ==> 1

node_37 --> "Scalenodon" 15 (#Palatine) 1 0.333 0 --> 1

94 (Upper inciso) 1 0.250 1 --> 2

95 (Lower inciso) 1 0.286 1 ==> 2

99 (Upper canine) 1 0.500 0 --> 1

node_42 --> Langbergia 17 (Zygomatic ar) 1 0.250 0 ==> 2

42 (Frontal-epip) 1 0.200 1 --> 0

114 (Number of l) 1 0.500 0 --> 1

node_62 --> node_61 9 (Parietal fora) 1 0.250 0 --> 1

29 (Vomer) 1 0.333 0 --> 1

56 (Route of the) 1 0.667 0 ==> 1

69 (#Rotation of) 1 0.750 0 ==> 1

70 (Curvature of) 1 0.500 0 ==> 1

74 (#Medial marg) 1 0.667 0 ==> 1

76 (#Lateral not) 1 0.600 1 ==> 2

77 (Articulation) 1 0.667 0 ==> 1

128 (Scapular co) 1 0.500 0 --> 1

node_61 --> node_60 5 (Premaxilla fo) 1 0.500 0 --> 1

15 (#Palatine) 1 0.333 0 --> 1

28 (Vomer exposu) 1 0.500 0 --> 1

30 (Ectopterygoi) 1 0.286 0 ==> 2

34 (Posterior ex) 1 0.500 0 ==> 1

37 (Contribution) 1 0.500 0 ==> 1

124 (Anapophysis) 1 0.200 1 ==> 0

130 (Procoracoid) 1 0.500 0 --> 1

136 (Dorsal prof) 1 0.500 0 --> 1

node_60 --> Chiniquodon 1 (#Premaxillary) 1 0.333 0 ==> 1

7 (Maxilla) 1 0.200 0 --> 1

17 (Zygomatic ar) 1 0.250 2 ==> 0

19 (Infraorbital) 1 0.286 0 ==> 1

20 (#Zygomatic a) 1 0.286 0 --> 1

26 (The notch se) 1 0.200 0 --> 1

35 (#The posteri) 1 0.250 0 --> 2

36 (Osseous pala) 1 0.500 0 --> 1

42 (Frontal-epip) 1 0.200 1 --> 0

node_60 --> node_59 8 (Profile of sk) 1 0.400 0 --> 2

18 (The anterove) 1 0.250 0 ==> 1

21 (Posterior ex) 1 0.667 0 ==> 1

35 (#The posteri) 1 0.250 0 --> 1

48 (Internal car) 1 0.333 1 ==> 0

105 (Posterior p) 1 0.250 1 ==> 0

117 (Axis of pos) 1 0.500 0 ==> 1

137 (Length of a) 1 0.500 0 ==> 1

node_59 --> Probainognathus 20 (#Zygomatic a) 1 0.286 0 --> 1

26 (The notch se) 1 0.200 0 --> 1

55 (Foramen and ) 1 0.333 0 ==> 1

81 (Squamosal ar) 1 0.667 0 --> 2

83 (#Lateral rid) 1 0.429 2 --> 0

118 (Upper tooth) 1 0.500 0 ==> 1

node_59 --> node_58 15 (#Palatine) 1 0.333 1 --> 2

16 (Sphenopalati) 1 0.333 0 ==> 1

82 (Dentary symp) 1 0.500 1 ==> 0

83 (#Lateral rid) 1 0.429 2 --> 3

86 (Mediolateral) 1 1.000 0 ==> 1

93 (Relationship) 1 0.600 0 ==> 1

103 (#Anteriormo) 1 0.500 0 ==> 1

106 (Upper postc) 1 0.750 0 ==> 1

107 (Lower postc) 1 0.500 0 ==> 1

108 (Buccal (ext) 1 1.000 0 ==> 1

115 (Lingual cin) 1 0.167 1 ==> 0

138 (Lateral sur) 1 1.000 0 ==> 1

139 (Posterior i) 1 1.000 0 ==> 1

140 (Cotyloid (a) 1 1.000 0 ==> 1

141 (The diamete) 1 1.000 0 --> 1

node_58 --> Prozostrodon 1 (#Premaxillary) 1 0.333 0 --> 2

94 (Upper inciso) 1 0.250 1 --> 0

95 (Lower inciso) 1 0.286 1 ==> 0

101 (Canine serr) 1 0.200 0 ==> 1

102 (Upper postc) 1 0.286 0 ==> 1

node_58 --> node_57 7 (Maxilla) 1 0.200 0 --> 1

12 (Parietal cre) 1 1.000 0 --> 1

13 (Prefrontal) 1 0.333 0 ==> 1

14 (#Postorbital) 1 1.000 0 ==> 2

17 (Zygomatic ar) 1 0.250 2 --> 1

36 (Osseous pala) 1 0.500 0 --> 1

123 (Posterior t) 1 1.000 0 --> 1

144 (Lesser troc) 1 1.000 0 ==> 1

145 (Lesser troc) 1 1.000 0 ==> 1

node_57 --> Therioherpeton 117 (Axis of pos) 1 0.500 1 ==> 0

node_57 --> node_56 2 (Septomaxilla ) 1 0.143 1 --> 0

10 (Interparieta) 1 1.000 0 --> 1

31 (Interpterygo) 1 0.167 1 --> 0

39 (The nasophar) 1 1.000 0 --> 1

41 (Quadrate art) 1 0.333 1 --> 0

42 (Frontal-epip) 1 0.200 1 --> 0

44 (The anterior) 1 0.500 0 --> 1

51 (Internal aud) 1 1.000 0 --> 1

58 (Pterygoparoc) 1 0.333 0 --> 2

63 (Separation o) 1 0.500 0 --> 1

67 (The relation) 1 0.500 0 --> 1

69 (#Rotation of) 1 0.750 1 --> 2

71 (Size of the ) 1 0.250 0 --> 1

73 (#Lateral mar) 1 1.000 1 --> 2

74 (#Medial marg) 1 0.667 1 --> 2

75 (Dorsal margi) 1 0.333 0 --> 1

76 (#Lateral not) 1 0.600 2 --> 3

78 (Articulation) 1 1.000 1 --> 3

79 (Craniomandib) 1 0.750 1 --> 2

120 (Vertebral c) 1 1.000 0 ==> 1

131 (Procoracoid) 1 1.000 0 --> 1

133 (Ulna olecra) 1 0.500 0 --> 1

142 (Femur head) 1 1.000 0 ==> 1

143 (Greater tro) 1 1.000 0 ==> 1

node_56 --> node_45 1 (#Premaxillary) 1 0.333 0 --> 2

4 (Paracanine fo) 1 1.000 0 ==> 3

46 (#Basisphenoi) 1 0.600 1 --> 2

68 (Shape of the) 1 0.333 0 ==> 1

94 (Upper inciso) 1 0.250 1 --> 2

96 (Incisor size) 1 0.500 0 --> 1

99 (Upper canine) 1 0.500 0 ==> 1

node_45 --> Riograndia 17 (Zygomatic ar) 1 0.250 1 ==> 2

27 (Palatine) 1 0.333 0 ==> 1

100 (Lower canin) 1 0.500 0 ==> 1

115 (Lingual cin) 1 0.167 0 ==> 1

node_45 --> Pachygenelus 35 (#The posteri) 1 0.250 1 ==> 2

65 (Paroccipital) 1 0.333 0 --> 1

81 (Squamosal ar) 1 0.667 0 --> 1

95 (Lower inciso) 1 0.286 1 ==> 2

106 (Upper postc) 1 0.750 1 ==> 0

107 (Lower postc) 1 0.500 1 ==> 0

117 (Axis of pos) 1 0.500 1 ==> 2

129 (Scapular el) 1 0.500 0 --> 1

node_56 --> node_55 38 (Middle of pt) 1 0.400 0 ==> 2

49 (Prootic and ) 1 0.333 0 ==> 1

56 (Route of the) 1 0.667 1 ==> 2

62 (Paroccipital) 1 0.500 0 ==> 1

65 (Paroccipital) 1 0.333 0 --> 1

121 (Axis centru) 1 1.000 0 --> 1

122 (Dens) 1 1.000 0 --> 1

132 (Humerus ect) 1 1.000 0 ==> 1

138 (Lateral sur) 1 1.000 1 ==> 2

node_55 --> node_49 8 (Profile of sk) 1 0.400 2 ==> 1

57 (#Maxillary a) 1 0.667 0 --> 1

59 (Vertical com) 1 0.500 0 ==> 1

60 (Anterior par) 1 0.500 0 ==> 1

79 (Craniomandib) 1 0.750 2 ==> 0

node_49 --> Pseudotherium 2 (Septomaxilla ) 1 0.143 0 ==> 1

7 (Maxilla) 1 0.200 1 ==> 0

13 (Prefrontal) 1 0.333 1 ==> 0

14 (#Postorbital) 1 1.000 2 ==> 1

15 (#Palatine) 1 0.333 2 ==> 1

18 (The anterove) 1 0.250 1 --> 0

40 (Quadrate ram) 1 0.500 1 ==> 0

46 (#Basisphenoi) 1 0.600 1 --> 3

50 (Promontorium) 1 0.500 0 --> 1

66 (Tabular) 1 0.333 0 ==> 1

103 (#Anteriormo) 1 0.500 1 --> 0

node_49 --> node_48 1 (#Premaxillary) 1 0.333 0 --> 1

6 (Maxillary pla) 1 0.500 0 ==> 1

20 (#Zygomatic a) 1 0.286 0 --> 2

21 (Posterior ex) 1 0.667 1 --> 0

22 (The posterov) 1 0.200 0 --> 1

24 (Squamosal gr) 1 0.500 0 ==> 1

25 (Posterior ex) 1 0.250 1 --> 0

35 (#The posteri) 1 0.250 1 ==> 2

45 (Parasphenoid) 1 1.000 0 ==> 1

52 (#The trigemi) 1 0.500 0 ==> 1

54 (Vascular for) 1 0.500 0 --> 1

61 (Hyoid (stape) 1 0.500 0 --> 1

63 (Separation o) 1 0.500 1 ==> 0

64 (Articulation) 1 0.250 0 --> 1

69 (#Rotation of) 1 0.750 2 --> 3

78 (Articulation) 1 1.000 3 --> 2

80 (Craniomandib) 1 1.000 0 ==> 2

84 (Angle of the) 1 0.500 0 --> 1

91 (Mandibular m) 1 0.667 0 --> 1

92 (Postcanine o) 1 0.667 0 ==> 1

93 (Relationship) 1 0.600 1 ==> 3

94 (Upper inciso) 1 0.250 1 --> 2

96 (Incisor size) 1 0.500 0 --> 1

99 (Upper canine) 1 0.500 0 ==> 2

100 (Lower canin) 1 0.500 0 --> 2

102 (Upper postc) 1 0.286 0 ==> 2

103 (#Anteriormo) 1 0.500 1 --> 2

104 (#Posteriorm) 1 0.667 0 ==> 2

106 (Upper postc) 1 0.750 1 ==> 3

107 (Lower postc) 1 0.500 1 --> 2

109 (Number of u) 1 0.400 0 ==> 2

112 (Arrangement) 1 0.500 0 ==> 1

117 (Axis of pos) 1 0.500 1 ==> 3

118 (Upper tooth) 1 0.500 0 ==> 2

119 (Postcanine ) 1 0.667 0 --> 2

127 (#Acromion p) 1 1.000 1 --> 2

node_48 --> Oligokyphus 67 (The relation) 1 0.500 1 ==> 0

71 (Size of the ) 1 0.250 1 ==> 0

75 (Dorsal margi) 1 0.333 1 ==> 0

node_48 --> node_47 3 (#Snout in rel) 1 0.250 1 --> 2

17 (Zygomatic ar) 1 0.250 1 --> 0

23 (The width of) 1 0.333 0 ==> 1

26 (The notch se) 1 0.200 0 ==> 1

34 (Posterior ex) 1 0.500 1 --> 0

44 (The anterior) 1 0.500 1 --> 0

48 (Internal car) 1 0.333 0 --> 1

node_47 --> node_46 31 (Interpterygo) 1 0.167 0 ==> 1

node_46 --> Tritylodon 22 (The posterov) 1 0.200 1 ==> 0

75 (Dorsal margi) 1 0.333 1 ==> 0

95 (Lower inciso) 1 0.286 1 ==> 2

node_46 --> Bienotherium 58 (Pterygoparoc) 1 0.333 2 ==> 0

node_47 --> Kayentatherium 27 (Palatine) 1 0.333 0 ==> 1

node_55 --> node_54 102 (Upper postc) 1 0.286 0 ==> 1

113 (Interlockin) 1 0.500 0 ==> 1

node_54 --> node_53 3 (#Snout in rel) 1 0.250 1 --> 0

18 (The anterove) 1 0.250 1 --> 0

46 (#Basisphenoi) 1 0.600 1 --> 2

47 (#Overlap of ) 1 1.000 0 --> 1

50 (Promontorium) 1 0.500 0 --> 1

53 (Lateral trou) 1 1.000 0 --> 1

54 (Vascular for) 1 0.500 0 --> 1

55 (Foramen and ) 1 0.333 0 --> 1

57 (#Maxillary a) 1 0.667 0 --> 2

100 (Lower canin) 1 0.500 0 ==> 1

119 (Postcanine ) 1 0.667 0 --> 1

129 (Scapular el) 1 0.500 0 --> 1

node_53 --> node_50 64 (Articulation) 1 0.250 0 --> 1

68 (Shape of the) 1 0.333 0 ==> 1

node_50 --> Brasilodon 49 (Prootic and ) 1 0.333 1 ==> 0

node_50 --> Brasilitherium 13 (Prefrontal) 1 0.333 1 ==> 0

17 (Zygomatic ar) 1 0.250 1 ==> 2

25 (Posterior ex) 1 0.250 1 --> 0

37 (Contribution) 1 0.500 1 ==> 0

94 (Upper inciso) 1 0.250 1 ==> 0

node_53 --> node_52 8 (Profile of sk) 1 0.400 2 ==> 0

11 (#Lateral exp) 1 1.000 0 ==> 1

52 (#The trigemi) 1 0.500 0 ==> 2

node_52 --> Adelobasileus 58 (Pterygoparoc) 1 0.333 2 ==> 1

62 (Paroccipital) 1 0.500 1 ==> 0

66 (Tabular) 1 0.333 0 --> 1

node_52 --> node_51 4 (Paracanine fo) 1 1.000 0 --> 2

27 (Palatine) 1 0.333 0 --> 1

31 (Interpterygo) 1 0.167 0 ==> 1

35 (#The posteri) 1 0.250 1 --> 2

43 (Epipterygoid) 1 1.000 0 ==> 1

46 (#Basisphenoi) 1 0.600 2 ==> 3

61 (Hyoid (stape) 1 0.500 0 ==> 1

79 (Craniomandib) 1 0.750 2 --> 3

81 (Squamosal ar) 1 0.667 0 --> 1

88 (#Postdentary) 1 1.000 1 --> 2

91 (Mandibular m) 1 0.667 0 --> 2

95 (Lower inciso) 1 0.286 1 --> 0

106 (Upper postc) 1 0.750 1 --> 2

107 (Lower postc) 1 0.500 1 --> 2

node_51 --> Sinoconodon 59 (Vertical com) 1 0.500 0 ==> 1

77 (Articulation) 1 0.667 1 --> 0

93 (Relationship) 1 0.600 1 --> 0

102 (Upper postc) 1 0.286 1 ==> 0

113 (Interlockin) 1 0.500 1 ==> 0

115 (Lingual cin) 1 0.167 0 ==> 1

node_51 --> Morganucodon 21 (Posterior ex) 1 0.667 1 ==> 2

30 (Ectopterygoi) 1 0.286 2 ==> 1

47 (#Overlap of ) 1 1.000 1 ==> 2

60 (Anterior par) 1 0.500 0 ==> 1

64 (Articulation) 1 0.250 0 --> 1

66 (Tabular) 1 0.333 0 --> 1

68 (Shape of the) 1 0.333 0 ==> 1

77 (Articulation) 1 0.667 1 --> 2

78 (Articulation) 1 1.000 3 --> 4

92 (Postcanine o) 1 0.667 0 ==> 2

93 (Relationship) 1 0.600 1 --> 2

124 (Anapophysis) 1 0.200 0 --> 1

node_61 --> Ectenion 2 (Septomaxilla ) 1 0.143 1 --> 0

8 (Profile of sk) 1 0.400 0 --> 2

15 (#Palatine) 1 0.333 0 --> 2

38 (Middle of pt) 1 0.400 0 ==> 2

52 (#The trigemi) 1 0.500 0 ==> 1

58 (Pterygoparoc) 1 0.333 0 ==> 1

83 (#Lateral rid) 1 0.429 2 --> 0

101 (Canine serr) 1 0.200 0 --> 1

node_63 --> Lumkuia 7 (Maxilla) 1 0.200 0 ==> 1

9 (Parietal fora) 1 0.250 0 --> 1

26 (The notch se) 1 0.200 0 --> 1

29 (Vomer) 1 0.333 0 --> 1

30 (Ectopterygoi) 1 0.286 0 ==> 2

35 (#The posteri) 1 0.250 0 ==> 1

38 (Middle of pt) 1 0.400 0 ==> 1

49 (Prootic and ) 1 0.333 0 ==> 1

58 (Pterygoparoc) 1 0.333 0 ==> 1

76 (#Lateral not) 1 0.600 1 ==> 0

node_65 --> Platycraniellus 19 (Infraorbital) 1 0.286 0 --> 1

20 (#Zygomatic a) 1 0.286 0 ==> 1

30 (Ectopterygoi) 1 0.286 0 --> 1

71 (Size of the ) 1 0.250 0 ==> 1

85 (Position of ) 1 0.500 0 --> 1
